# Chemical Constituents of the Deep‐Sea‐Derived Fungus *Talaromyces wortmannii* and Their Ferroptosis Inhibitory Activity

**DOI:** 10.1002/cbdv.71678

**Published:** 2026-09-15

**Authors:** Ke‐Xin Ma, Zheng‐Biao Zou, Qi‐Qian Wu, Li‐Sheng Li, Ying‐Ni Pan, Xian‐Wen Yang

**Affiliations:** ^1^ School of Traditional Chinese Materia Medica Shenyang Pharmaceutical University Shenyang China; ^2^ Hainan Academy of Medical Sciences Hainan Medical University Haikou China; ^3^ School of Basic Medical Sciences Fujian Medical University Fuzhou China

**Keywords:** ferroptosis inhibitory activity, fungus, deep sea, secondary metabolites, *Talaromyces wortmannii*

## Abstract

Deep‐sea‐derived fungi have emerged as a prolific source of structurally novel and pharmacologically active secondary metabolites, representing an invaluable reservoir of lead compounds for drug discovery and development. In the present study, one new tyramine derivative (**1**) and 28 known compounds (**2–29**) were obtained from *Talaromyces wortmannii* MCCC 3A01136, a fungus isolated from the intestinal contents of a stranded deep‐sea whale (*Mesoplodon densirostris*) in the East China Sea. The structures were elucidated via comprehensive analysis of 1D and 2D NMR data, HRESIMS, NMR computations coupled with DP4+ probability analysis, and ECD calculations. Comazaphilones E (**2**) and C (**6**) significantly inhibited renin‐angiotensin system‐selective lethal 3 (RSL3)‐induced ferroptosis with a half maximal effective concentration (EC_50_) value of 4.52 and 13.73 µM, respectively.

## Graphical abstract

One new tyramine derivative (**1**) and 28 known compounds (**2**–**29**) were isolated from deep‐sea‐derived fungus *Talaromyces wortmannii*. Their structures were elucidated by NMR/MS and computational calculations. Compounds **2** and **6** potently inhibited ferroptosis with EC_5_₀ values of 4.52 and 13.73 µM.

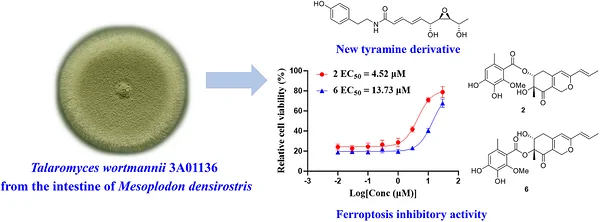

## 1 Introduction

**FIGURE 1:**
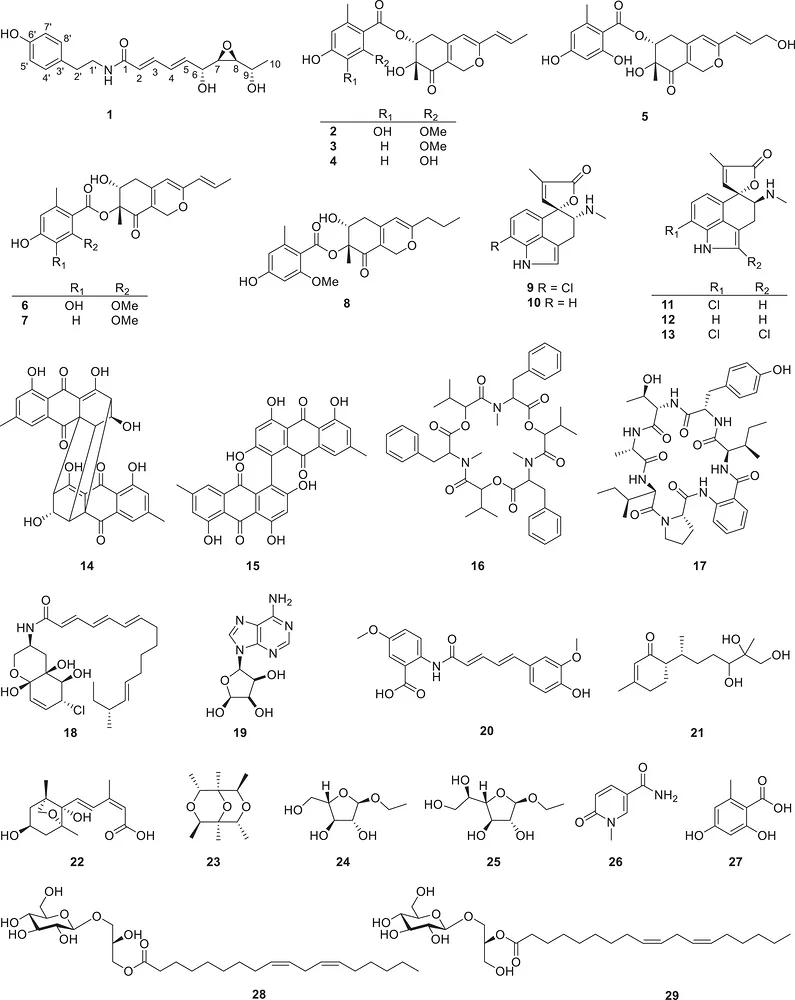
Chemical structures of compounds **1**−**29** of the deep‐sea‐derived *Talaromyces wortmannii*.

The genus *Talaromyces* comprises fungi widely distributed across terrestrial and marine environments, where they frequently associate with plants, animals, and marine organisms such as sponges [1, 2]. Marine‐derived *Talaromyces* strains have attracted considerable interest owing to their prolific production of structurally diverse secondary metabolites [3, 4, 5]. Since 2016, over 500 such metabolites have been reported from axenic cultures of these isolates, with approximately 45% being new compounds [6]. These metabolites span a wide range of chemical classes, including alkaloids, meroterpenoids, isocoumarins, anthraquinones, xanthones, phenalenones, benzofurans, azaphilones, and other polyketides [6, 7, 8, 9]. This impressive chemodiversity is complemented by a broad spectrum of biological activities, such as anti‐inflammatory, antibacterial, antifungal, antitumor, hypolipidemic, and nematocidal effects [3, 6, 10, 11], which positions these fungi as a valuable source of leads for biotechnological and pharmaceutical applications. In our ongoing efforts to discover bioactive metabolites from marine‐derived fungi [12, 13, 14, 15, 16], the fungus *Talaromyces wortmannii* MCCC 3A01136 was isolated from the intestinal contents of a stranded deep‐sea whale (*Mesoplodon densirostris*) in the East China Sea. Subsequent chemical investigation of the EtOAc extract of the fermentation broth yielded one new tyramine derivative, named wortyramine A (**1**), along with 28 known secondary metabolites (**2**–**29**) (Figure 1). Herein, the detailed isolation, structural elucidation, and biological activities of these compounds are reported.

## 2 Results and Discussion

Compound **1** was obtained as a colorless oil. On the basis of its high‐resolution electrospray ionization mass spectrometry (HRESIMS) data (*m/z*: [M + H]^+^ calculated: 334.1649; observed: 334.1642; Figure S7), the molecular formula of **1** was inferred as C_18_H_23_NO_5_, corresponding to eight degrees of unsaturation. The ^1^H NMR (Table 1 and Figure S1) spectrum showed four signals of exchangeable protons that were assigned to three hydroxyl groups [*δ*_H_ 9.17 (1H, s, OH‐6'), 5.31 (1H, d, *J* = 4.4 Hz, OH‐6), 4.81 (1H, d, *J* = 4.2 Hz, OH‐9)] and one amide group [*δ*_H_ 8.06 (1H, d, *J* = 5.6 Hz, NH‐1'], four benzene protons at [*δ*_H_ 6.99 (2H, d, *J* = 8.3 Hz, H‐4', 8'), 6.67 (2H, d, *J* = 8.3 Hz, H‐5', 7')], two *sp* ^2^ ethylene groups [*δ*_H_ 7.03 (1H, dd, *J* = 11.2, 15.2 Hz, H‐3), 6.37 (1H, dd, *J* = 11.1, 15.2 Hz, H‐4), 6.13 (1H, dd, *J* = 5.3, 15.3 Hz, H‐5), 6.01 (1H, d, *J* = 15.1 Hz, H‐2)], four oxygenated methines [*δ*_H_ 4.00 (1H, m, H‐6), 3.58 (1H, m, H‐9), 2.84 (1H, dd, *J* = 3.5, 8.0 Hz, H‐7), 2.78 (1H, dd, *J* = 3.8, 8.0 Hz, H‐8)], two *sp* ^3^ methylene groups [*δ*_H_ 3.29 (2H, q, *J* = 6.8 Hz, H‐1'), 2.61 (2H, t, *J* = 7.5 Hz, H‐2')] and one methyl group at *δ*_H_ 1.19 (3H, d, *J* = 6.2 Hz, H‐10).

**TABLE 1:** ^1^H and ^13^C NMR data of compound **1** in DMSO‐*d*_6_.

NO.	*δ*_C_, Type	*δ*_H_, mult (*J* in Hz)
1	164.9 C	
2	125.4 CH	6.01 d (15.1)
3	138.1 CH	7.03 dd (11.2, 15.2)
4	128.1 CH	6.37 dd (11.1, 15.2)
5	140.6 CH	6.13 dd (5.3, 15.3)
6	67.4 CH	4.00 m
7	58.9 CH	2.84 dd (3.5, 8.0)
8	60.4 CH	2.78 dd (3.8, 8.0)
9	63.1 CH	3.58 m
10	21.2 CH_3_	1.19 d (6.2)
1'	40.6 CH_2_	3.29 q (6.8)
2'	34.3 CH_2_	2.61 t (7.5)
3'	129.5 C	
4',8'	129.4 CH	6.99 d (8.3)
5',7'	115.1 CH	6.67 d (8.3)
6'	155.6 C	
6‐OH		5.31 d (4.4)
9‐OH		4.81 d (4.2)
1'‐NH		8.06 d (5.6)
6'‐OH		9.17 s

Eighteen carbon signals in the ^13^C NMR data (Table 1 and Figure S2) correspond to one amide carbonyl carbon (*δ*_C_ 164.9), one benzene ring (*δ*_C_ 155.6, 129.5, 129.4, 115.1), four olefins (*δ*_C_ 140.6, 138.1, 128.1, 125.4), four methines (*δ*_C_ 67.4, 63.1, 60.4, 58.9), two methylenes (*δ*_C_ 40.6, 34.3) and a methyl group (*δ*_C_ 21.2).

**FIGURE 2:**
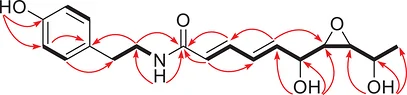
The COSY (bold) and HMBC (red arrows) correlations of **1**.

The planar structure of **1** was established by the 2D NMR data (Figures S3–S6). A tyramine unit was deduced by the ^1^H–^1^H COSY correlations (1'‐NH/H_2_‐1'/H_2_‐2', H‐4'/H‐5') and the HMBC correlations from H_2_‐1' to C‐3', from H‐4' to C‐2', from H‐5' to C‐3', from 1'‐NH to C‐1', and from 6'‐OH to C‐5' and C‐6'. The ^1^H–^1^H COSY correlations of H‐2/H‐3/H‐4/H‐5/H‐6/H‐7/H‐8/H‐9/H_3_‐10 connected a long chain of C‐2/C‐3/C‐4/‐5/C‐6/C‐7/C‐8/C‐9/C‐10 (Figure 2). In the HMBC spectrum, correlations from H‑2 to C‑1/C‑4, from H‑3 to C‑1, H‑5 to C‑3/C‑7, from 6‑OH to C‑5/C‑6/C‑7, and from 9‑OH to C‑8/C‑9/C‑10 allowed unambiguous establishment of the fatty acid chain skeleton. Finally, the fatty acid chain was linked to the tyramine unit through the nitrogen atom, as evidenced by HMBC correlations from H_2_‑1' to C‑1 and from 1'‐NH to C‑1. Accordingly, the planar structure of **1** was determined as depicted.

**FIGURE 3:**
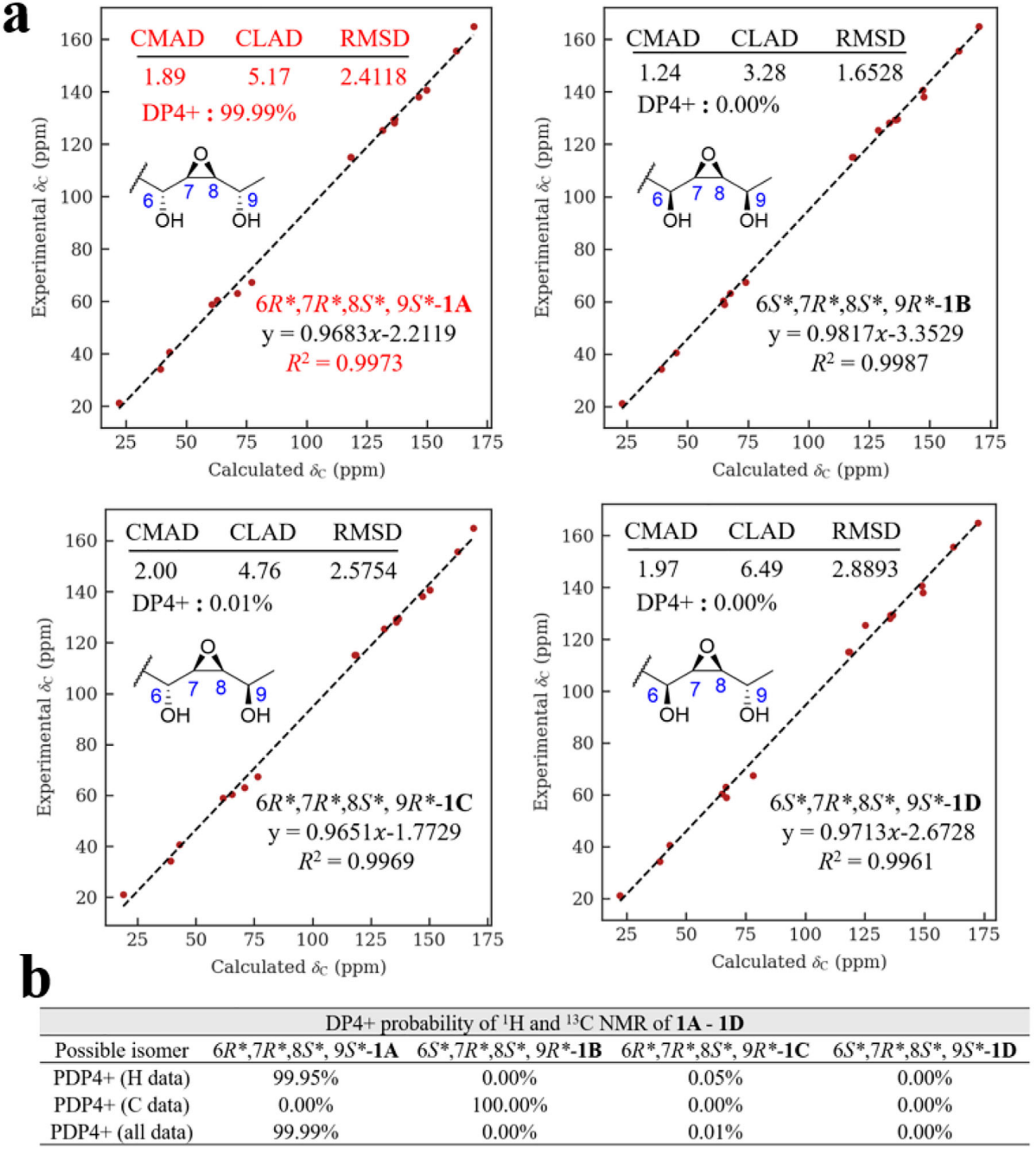
DP4+ probability analysis derived from NMR calculations for compound **1**: displaying the linear correlations between experimental and calculated ^1^ ^3^C NMR chemical shifts for the four possible isomers, along with the corresponding DP4+ probabilities.

The two double bonds in the fatty acid chain were all assigned the *E* geometry on the basis of the coupling constants (^3^ *J*_H‐2/H‐3_ = 15.1 Hz and ^3^ *J*_H‐4/H‐5_ = 15.2 Hz). The mutually coupled signals of H‐7 and H‐8 with a ^3^ *J*_H‐7/H‐8_ value of 8.0 Hz were assigned as a *cis*‐form epoxide moiety (Table 1). In order to determine the stereochemistry at C‑6, C‑7, C‑8, and C‑9 of **1**, theoretical NMR chemical shift calculations were carried out for four possible isomers, namely **1A** (6R*,7R*,8S*,9S*),**1B** (6S*,7R*,8S*,9R*), **1C** (6R*,7R*,8S*,9R*), and **1D** (6S*,7R*,8S*,9S*). These calculations were performed using the GIAO method at the mPW1PW91/6‐311G(2d,p) level in DMSO with the IEFPCM model (Figure 3). As a result, the calculated ^13^C NMR chemical shifts of **1A** were consistent with those of the experimental data, with good linear correlation coefficients (*R* ^2^ = 0.9973), corrected mean absolute deviations (CMAD, 1.89), corrected largest absolute deviations (CLAD, 5.17), and root‐mean‐square deviations (RMSD, 2.4118) (Figure 3a). Further DP4+ analyses based on both ^1^H and ^13^C NMR data indicated that **1A** (6R*,7R*,8S*,9S*) was the most reliable structure with 99.99% probability (Figure 3b).

**FIGURE 4:**
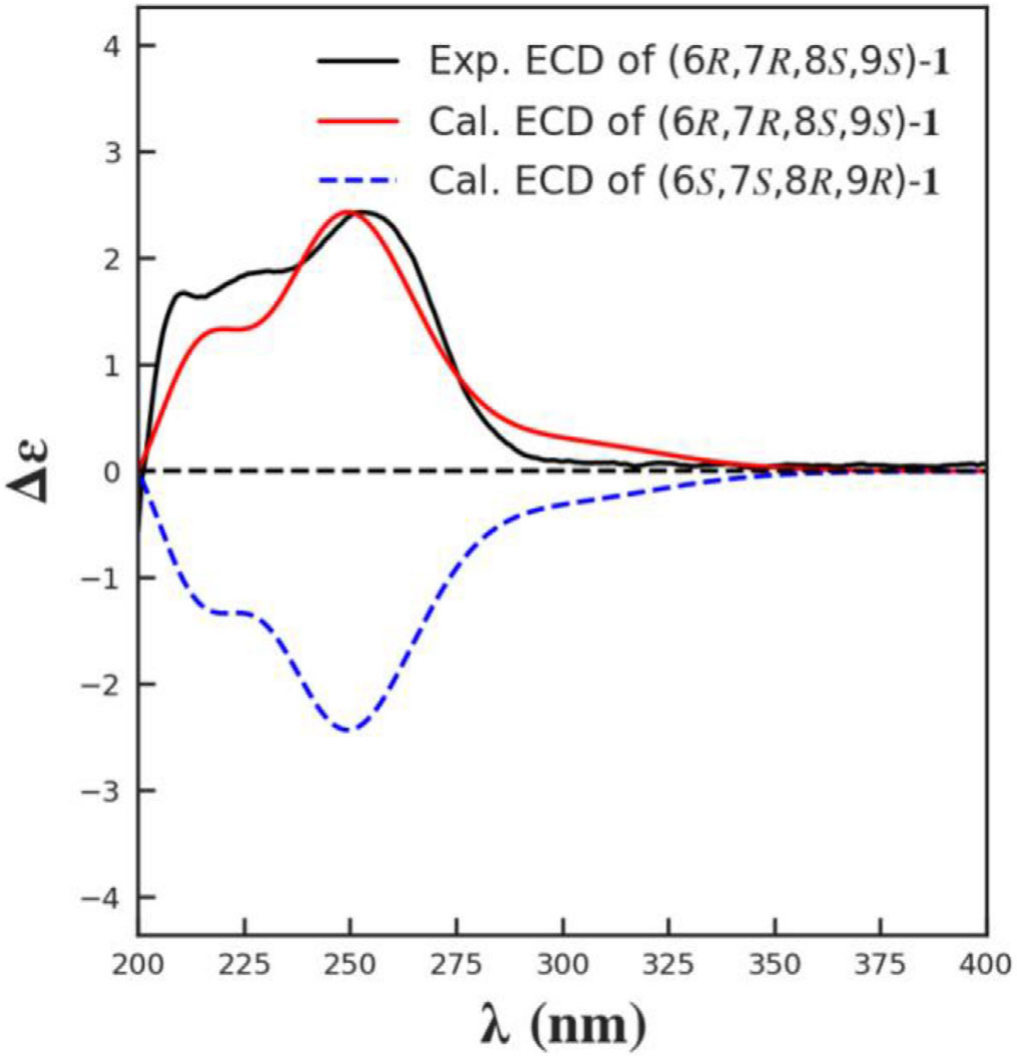
Experimental and calculated ECD spectra of compound **1**.

To further establish the absolute configuration of compound **1**, theoretical ECD calculations were performed for both (6*R*,7*R*,8*S*,9*S*)‐**1** and its enantiomer (6*S*,7*S*,8*R*,9*R*)‐**1** (Figure S93). As shown in Figure 4, the experimental ECD spectrum matched well with that of the (6*R*,7*R*,8*S*,9*S*)‐**1**. Hence, compound **1** was identified as a new tyramine derivative and named wortyramine A.

One new tyramine derivative (**1**) and 28 known compounds (**2**−**29**) were obtained from the extract of *T. wortmannii* MCCC 3A01136, using repeated column chromatography (CC) over silica gel, Sephadex LH‐20, and octadecylsilyl (ODS). By comparing the NMR, MS, and OR data with the references (Figures S9–S92), 28 known compounds were identified as comazaphilone E (**2**) [17], comazaphilone G (**3**) [18], comazaphilone D (**4**) [17], entonaemin A (**5**) [19], comazaphilone C (**6**) [20], kasanosin C (**7**) [21], comazaphilone I (**8**) [22], 8‐chlororugulovasine A (**9**) [23], rugulovasine A (**10**) [23], 8‐chlororugulovasine B (**11**) [23], rugulovasine B (**12**) [23], 2,8‐dichlororugulovasine B (**13**) [23], rugulosin A (**14**) [24], (‐)‐*R*‐skyrin (**15**) [25], beauvericin (**16**) [26], scortide A (**17**) [27], varitatin A (**18**) [28], adenosine (**19**) [29], avenanthramide P (**20**) [30], scorzodivaricin A (**21**) [31], dihydrophaseic acid (**22**) [32], nesterenkoniane (**23**) [33], ethyl *α*‐*L*‐arabinofuranoside (**24**) [34], ethyl *β*‐*D*‐galactofuranoside (**25**) [35], nudifloric acid (**26**) [36], orsellinic acid (**27**) [37], 3‐*α*‐linolenoylglycerol 1‐*O*‐*β*‐D‐galactopyranoside (**28**) [38], and 2‐*α*‐linolenoylglycerol 1‐*O*‐*β*‐D‐galactopyranoside (**29**) [38].

**FIGURE 5:**
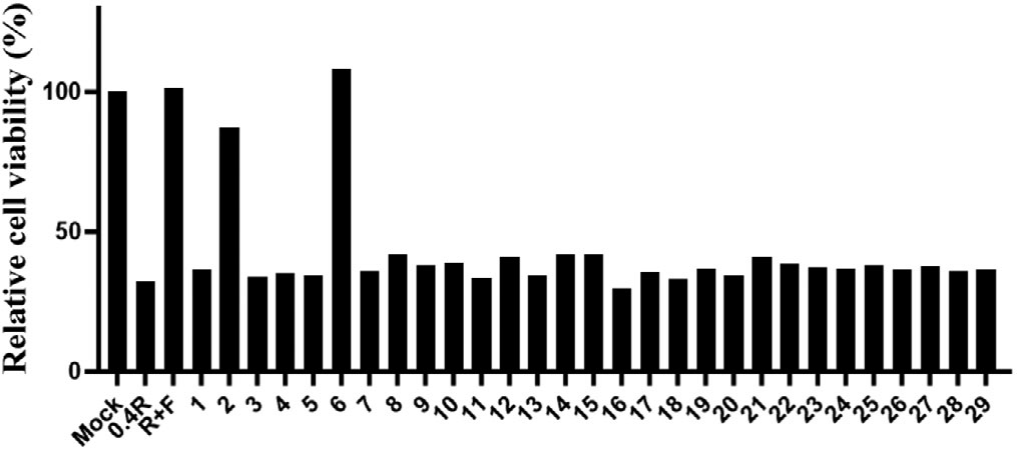
A375 cells were pretreated with the test compounds (10 µM) for 1 h and subsequently exposed to RSL3 (0.4 µM) to induce ferroptosis. Cell viability was determined using the CellTiter‑Glo Luminescent Cell Viability Assay, and the values were normalized to the control (DMSO only). Fer‑1 (10 µM) was employed as a positive control. Data are presented as relative cell viability (%).

Ferroptosis is a regulated form of non‐apoptotic cell death driven by iron‐dependent lipid peroxidation and oxidative damage [39]. This process has been implicated in a broad spectrum of pathophysiological conditions, including aging, neurodegenerative disorders, stroke, and organ ischemia‑reperfusion injury, suggesting that pharmacological inhibition of ferroptosis may offer a promising therapeutic strategy for these diseases [40, 41]. RSL3 is a well‑characterized ferroptosis inducer capable of markedly decreasing A375 cell viability. In contrast, the ferroptosis inhibitor ferrostatin‑1 (Fer‑1), at 10 µM, fully reversed the RSL3‑induced ferroptotic effect in A375 cells. As shown in Figure 5, comazaphilones E (**2**) and C (**6**) at a concentration of 10 µM significantly reversed RSL‐3‐induced cytotoxicity, exhibiting a stronger protective effect than ferrostatin‑1 (Fer‑1).

**FIGURE 6:**
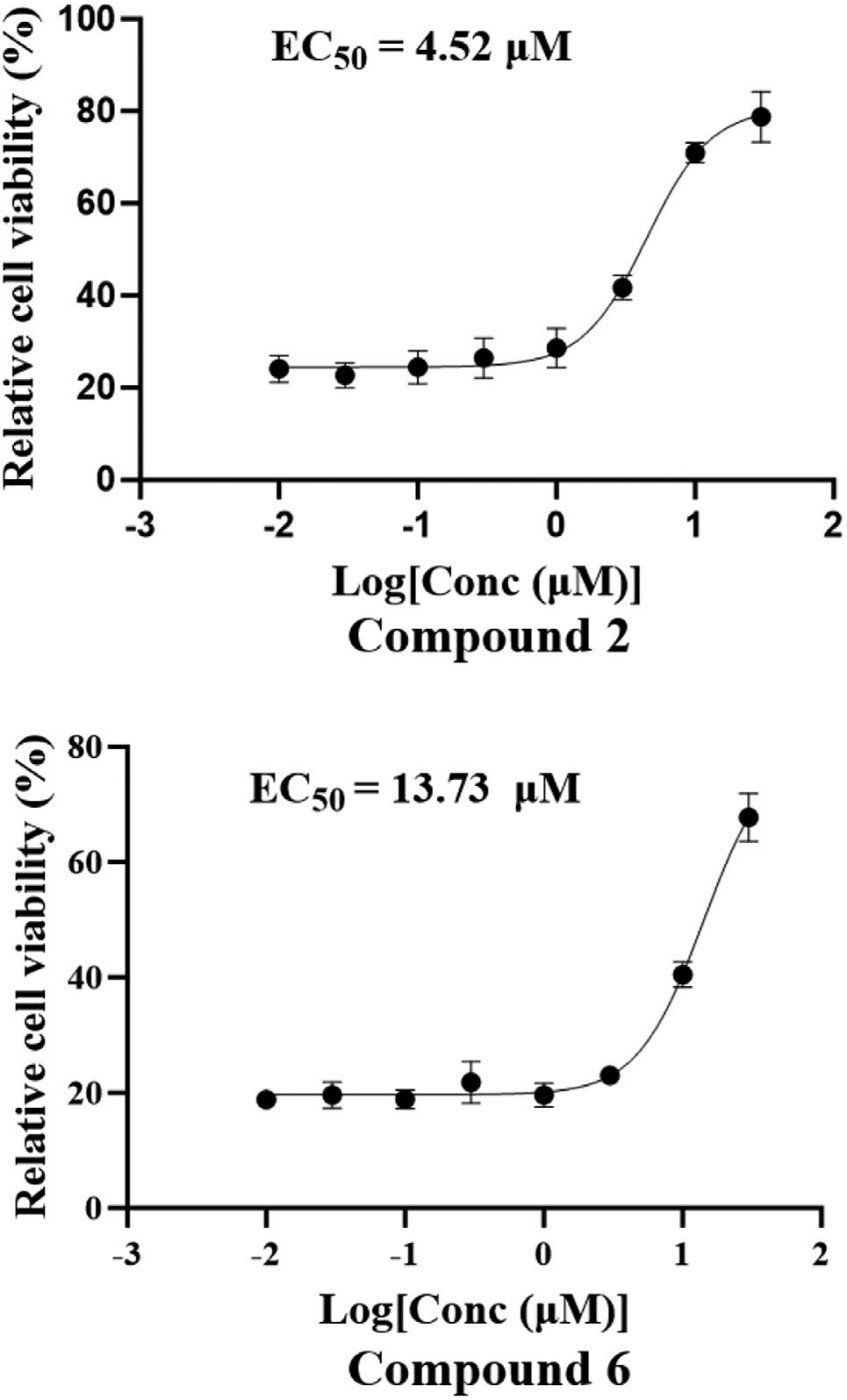
Compounds **2** and **6** inhibited RSL3‐induced ferroptosis in a dose‐dependent manner in A375 cells.

Among the tested compounds, **2** and **6** were the most potent, with half maximal effective concentration (EC_50_) values of 4.52 and 13.73 µM (Figure 6), respectively.

## 3 Conclusions

In summary, one new tyramine derivative (**1**) and 28 known compounds (**2**–**29**) were isolated from the deep‐sea‐derived fungus *Talaromyces wortmannii* MCCC 3A01136. Comazaphilones E (**2**) and C (**6**) exhibited potent inhibition against RSL3‐induced ferroptosis.

## 4 Experimental Section

### 4.1 General Experimental Procedures

Optical rotations were measured on an Anton Paar MCP 100 polarimeter (Anton Paar Trading Co. Ltd., Shanghai, China). NMR spectra were acquired in DMSO‐*d*_6_ and CD_3_OD on an ECZ Luminous 600 MHz spectrometer (JEOL Ltd., Akishima, Tokyo, Japan). The HRESIMS spectra were recorded on a Sciex ZenoTOF 7600 mass spectrometer (SCIEX, Framingham, MA, USA). The data was processed using PeakView software. The medium‐pressure liquid chromatography (MPLC) was performed on a Buchi Sepacore (BÜCHI Labortechnik AG, Flawil, Switzerland). Semi‑preparative HPLC was carried out on an Agilent Technologies 1260 Infinity instrument (Agilent Technologies, San Diego, CA, USA) equipped with an ODS‐C18 column (10 mm × 250 mm, 5 µm). Column chromatography (CC) was conducted on silica gel or Sephadex LH‐20. The thin‐layer chromatography (TLC) plates were visualized under UV light or by spraying with 10% H_2_SO_4_.

### 4.2 Biological Material

*Talaromyces wortmannii* (GenBank accession No. NRRL 58838) was isolated from the intestinal contents of a stranded deep‐sea whale (*Mesoplodon densirostris*) in the East China Sea. The reference strain was obtained from the Marine Culture Collection of China (MCCC) with the accession number 3A01136.

### 4.3 Fermentation, Extraction, and Isolation

The strain was cultured on potato dextrose agar (PDA) at 25 °C for 3 days, then inoculated into seed medium (potato dextrose broth, PDB) and incubated on a rotary shaker at 150 rpm for 3 days. The seed culture was transferred to 85 × 1 L Erlenmeyer flasks, each containing 100 g oats, 1.5% (w/w) sea salt, and 120 mL distilled water, and incubated statically at 25 °C for 25 days. The fermented oat solid medium was extracted three times with ethyl acetate (EtOAc). The combined organic layers were concentrated under reduced pressure to afford the crude extract (245.8 g).

The crude extract was subjected to MPLC over silica gel eluted with CH_2_Cl_2_─MeOH (100:1, 50:1, 30:1, 20:1, 10:1 and 5:1) to afford four fractions (Fr.1–Fr.4) based on TLC analysis. Fraction Fr.1 (12.5 g) was subjected to MPLC on ODS (MeOH─H_2_O, 20→100%) to yield six subfractions (Fr.1‑1–Fr.1‑6). Subfraction Fr.1‑1 (74.7 mg) was purified by semi‑preparative HPLC (MeOH─H_2_O, 45% → 60%, *v* = 3 mL/min) to afford **5** (3.6 mg, *t*_R_ = 21 min). Subfraction Fr.1‑2 (850.0 mg) was subjected to Sephadex LH‑20 column chromatography (MeOH) to afford **13** (94.6 mg). Subfraction Fr.1‑3 (350.5 mg) was subjected to Sephadex LH‑20 column chromatography (MeOH), followed by HPLC (MeOH─H_2_O, 50% → 75%, *v* = 3 mL/min) to afford **3** (3.3 mg, *t*_R_ = 29 min). Subfraction Fr.1‐4 (713.1 mg) was subjected to Sephadex LH‐20 column chromatography (MeOH), followed by HPLC (MeOH─H_2_O, 55% → 80%, *v* = 3 mL/min) to afford **7** (32.1 mg, *t*_R_ = 20 min) and **8** (2.0 mg, *t*_R_ = 22 min). Subfraction Fr.1‐5 (95.5 mg) was subjected to Sephadex LH‐20 column chromatography (MeOH), followed by HPLC (MeOH─H_2_O, 60% → 85%, *v* = 3 mL/min) to afford **4** (14.2 mg, *t*_R_ = 27 min). Subfraction Fr.1‐6 (126.7 mg) was purified by Sephadex LH‐20 column chromatography (MeOH) to afford **15** (12.5 mg).

Fraction Fr.2 (12.6 g) was subjected to MPLC on ODS (MeOH─H_2_O, 10→100%) to yield nine subfractions (Fr.2‑1–Fr.2‑9). Subfraction Fr.2‑1 (161.1 mg) was subjected to Sephadex LH‑20 column chromatography (MeOH), followed by repeated silica gel column chromatography (CH_2_Cl_2_─MeOH, 80:1 → 70:1) to obtain **23** (30.6 mg) and **24** (7.8 mg).

Subfraction Fr.2‑2 (50.4 mg) was purified by semi‑preparative HPLC (MeOH─H_2_O, 82%, isocratic, *v* = 3 mL/min) to afford **18** (13.1 mg, *t*_R_ = 22 min). Subfraction Fr.2‑3 (157.9 mg) was subjected to Sephadex LH‑20 column chromatography (MeOH) to afford **26** (7.5 mg). Subfraction Fr.2‑4 (72.9 mg) was subjected to Sephadex LH‑20 column chromatography (MeOH), followed by repeated silica gel column chromatography (CH_2_Cl_2_─MeOH, 100:1 → 30:1) to afford **9** (7.3 mg) and **27** (7.0 mg). Subfraction Fr.2‑5 (176.2 mg) was subjected to Sephadex LH‑20 column chromatography (MeOH), followed by HPLC (MeOH─H_2_O, 40% → 60%, *v* = 3 mL/min) to afford **21** (1.2 mg, *t*_R_ = 19 min). Subfraction Fr.2‑6 (150 mg) was subjected to Sephadex LH‑20 column chromatography (MeOH), followed by repeated silica gel column chromatography (CH_2_Cl_2_─MeOH, 250:1 → 150:1) to afford **11** (11.3 mg). Subfraction Fr.2‑7 (130.8 mg) was subjected to Sephadex LH‑20 column chromatography (MeOH), followed by HPLC (MeOH─H_2_O, 50% → 75%, *v* = 3 mL/min) to afford **2** (3.5 mg, *t*_R_ = 29 min) and **6** (16.1 mg, *t*_R_ = 27 min). Subfraction Fr.2‑8 (54.7 mg) was subjected to Sephadex LH‑20 column chromatography (MeOH), followed by HPLC (MeOH─H_2_O, 50% → 75%, *v* = 3 mL/min) to afford **17** (9.2 mg, *t*_R_ = 25 min). Subfraction Fr.2‑9 (157.9 mg) was purified by Sephadex LH‑20 column chromatography (MeOH) to afford **16** (2.1 mg).

Fraction Fr.3 (10.5 g) was subjected to MPLC on ODS (MeOH─H_2_O, 5→85%) to yield seven subfractions (Fr.3‑1–Fr.3‑7). Subfraction Fr.3‑1 (28.7 mg) was purified by silica gel column chromatography (CH_2_Cl_2_─MeOH, 20:1 → 8:1) to afford **25** (4.2 mg). Subfraction Fr.3‑2 (60.8 mg) was purified by silica gel column chromatography (CH_2_Cl_2_─MeOH, 30:1 → 10:1) to afford **22** (6.1 mg). Subfraction Fr.3‑3 (434.1 mg) was subjected to Sephadex LH‑20 column chromatography (MeOH), followed by repeated silica gel column chromatography (CH_2_Cl_2_─MeOH, 80:1 → 30:1) to afford **10** (11.9 mg) and **12** (4.8 mg). Subfraction Fr.3‑4 (55.8 mg) was purified by HPLC (MeOH─H_2_O, 20% → 60%, *v* = 3 mL/min) to afford **1** (4.0 mg, *t*_R_ = 23 min). Subfraction Fr.3‑5 (133.7 mg) was subjected to Sephadex LH‑20 column chromatography (MeOH) to afford **20** (12.5 mg). Subfraction Fr.3‑6 (152.2 mg) was subjected to Sephadex LH‑20 column chromatography (MeOH), followed by HPLC (MeOH─H_2_O, 65% → 85%, *v* = 3 mL/min) to afford **14** (9.7 mg, *t*_R_ = 23 min). Subfraction Fr.3‑7 (3.4 g) was subjected to Sephadex LH‑20 column chromatography (MeOH), followed by HPLC (MeOH─H_2_O, 80% → 100%, *v* = 3 mL/min) to afford **28** (60.0 mg, *t*_R_ = 19 min) and **29** (30.7 mg, *t*_R_ = 18 min).

Fraction Fr.4 (8.0 g) was subjected to MPLC on ODS (MeOH–H_2_O, 5→100%), and the resulting fraction was further purified by trituration to afford **19** (22.4 mg).

Wortyramine A (**1**): colourless oil; [*α*]25 D + 54.324 (*c* 0.37, MeOH; Figure S8); UV (MeOH) *λ*_max_ (log *ε*) 205 (1.01), 256 (2.17) nm; ECD (MeOH) *λ*_max_ (Δ*ε*) 253 (*+*0.49) nm; ^1^H (600 MHz) and ^13^C (150 MHz) NMR data, see Table 1; HRESIMS *m/z* 334.1649 [M + H]^+^ (calcd for C_18_H_24_NO_5_, 334.1642).

### 4.4 ECD Calculations

Conformational analyses were performed using Gaussian 09. For each compound, conformers within a 7 kcal/mol energy window were retained, with an RMSD cutoff of 0.2 Å and a maximum of 100 conformers per compound. These initial conformers were first subjected to semiempirical PM6 geometry optimization in Gaussian 09, after which duplicated structures were identified and removed. The remaining conformers were then sequentially optimized at the HF/6‐31G(d) and B3LYP/6‐31G(d) levels, respectively, using the same software. Harmonic vibrational frequency calculations were carried out to verify the stability of the final conformers. For each optimized conformer, the ECD spectrum was computed via time‑dependent density functional theory (TDDFT) at the B3LYP/6‑311G(d,p) level, employing the IEFPCM solvation model with MeOH as the solvent. Finally, both the calculated and experimental ECD curves were generated with Gaussian 09, applying a Gaussian broadening of σ = 0.3 eV and a UV shift of 10 nm.

### 4.5 NMR Calculation

The structures were obtained directly from the preceding ECD calculations. GIAO (Gauge‑Including Atomic Orbitals) NMR computations were carried out at the MPW1PW91/6‑311+G(2d,p) level, with DMSO simulated using the IEFPCM solvation model. The TMS‑referenced chemical shifts were averaged according to Boltzmann populations, and linear regression analysis was subsequently applied to derive the final calculated ^1^ ^3^C NMR data.

### 4.6 DP4+ Probability Analysis

DP4+ analysis was performed using the calculated shielding tensors and experimental chemical shifts, with the Excel template provided by the original authors [42]. Within this template, the calculations were carried out with the mPW1PW91 functional, the 6‑311+G(d,p) basis set, and the PCM solvation model.

### 4.7 Ferroptosis Inhibitory Activity

As reported previously [43], human melanoma A375 cells (RRID: CVCL_0132) were grown in Dulbecco's modified Eagle's medium (DMEM) supplemented with 10% fetal bovine serum, 4 mM L‑glutamine, 100 IU penicillin, and 100 mg/mL streptomycin. The cells were maintained at 37 °C in a humidified atmosphere containing 5% CO_2_. The bioassay was conducted according to a published procedure [44]. Briefly, A375 cells were seeded into 96‑well plates and exposed to the test compounds for 1 h prior to the addition of RSL3 to trigger ferroptosis. Cell viability was evaluated using the CCK‑8 method, with absorbance measured using a MULTISKAN GO microplate reader (Thermo Scientific), or alternatively by means of the CellTiter‑Glo Luminescent Cell Viability Assay kit in accordance with the manufacturer's instructions.

## Author Contributions

**Ke‐Xin Ma**: investigation. **Zheng‐Biao Zou**: validation, writing – original draft, data curation. **Qi‐Qian Wu**: investigation. **Li‐Sheng Li**: investigation. **Ying‐Ni Pan**: Formal analysis. **Xian‐Wen Yang**: supervision, project administration, methodology.

## Conflicts of Interest

The authors declare no conflicts of interest.

## Supporting information




**Supporting File 1**: cbdv71678‐sup‐0001‐SuppMat.pdf.

## Data Availability

The data that support the findings of this study are available in the Supporting Information of this article.
