# Supplementary material for: Chemical Constituents of the Deep‐Sea‐Derived Fungus Talaromyces wortmannii and Their Ferroptosis Inhibitory Activity
Source: Chem Biodivers. 2026 Sep 15;23(9):e71678. doi: 10.1002/cbdv.71678 (PMC13575948; doi:10.1002/cbdv.71678)
Supplement: Supplementary file 1 [file CBDV-23-e71678-s001.pdf]

Content

Figure S1. ^{1}H NMR (600 MHz) spectrum of compound **1** in DMSO-d_6.
Figure S2. ^{13}C NMR (151 MHz) spectrum of compound **1** in DMSO-d_6.
Figure S3. HSQC spectrum of compound **1** in DMSO-d_6.
Figure S4. ^{1}H-^{1}H COSY spectrum of compound **1** in DMSO-d_6.
Figure S5. HMBC spectrum of compound **1** in DMSO-d_6.
Figure S6. NOESY spectrum of compound **1** in DMSO-d_6.
Figure S7. The positive HR-ESI-MS spectrum of compound **1**.
Figure S8. Optical rotation spectrum of compound **1**.
Figure S9. ^{1}H NMR (600 MHz) spectrum of compound **2** in DMSO-d_6.
Figure S10. ^{13}C NMR (151 MHz) spectrum of compound **2** in DMSO-d_6.
Figure S11. The positive HR-ESI-MS spectrum of compound **2**.
Figure S12. ^{1}H NMR (600 MHz) spectrum of compound **3** in DMSO-d_6.
Figure S13. ^{13}C NMR (151 MHz) spectrum of compound **3** in DMSO-d_6.
Figure S14. The positive HR-ESI-MS spectrum of compound **3**.
Figure S15. ^{1}H NMR (600 MHz) spectrum of compound **4** in DMSO-d_6.
Figure S16. ^{13}C NMR (151 MHz) spectrum of compound **4** in DMSO-d_6.
Figure S17. The positive HR-ESI-MS spectrum of compound **4**.
Figure S18. ^{1}H NMR (600 MHz) spectrum of compound **5** in DMSO-d_6.
Figure S19. ^{13}C NMR (151 MHz) spectrum of compound **5** in DMSO-d_6.
Figure S20. The positive HR-ESI-MS spectrum of compound **5**.
Figure S21. ^{1}H NMR (600 MHz) spectrum of compound **6** in DMSO-d_6.
Figure S22. ^{13}C NMR (151 MHz) spectrum of compound **6** in DMSO-d_6.
Figure S23. The positive HR-ESI-MS spectrum of compound **6**.
Figure S24. ^{1}H NMR (600 MHz) spectrum of compound **7** in DMSO-d_6.
Figure S25. ^{13}C NMR (151 MHz) spectrum of compound **7** in DMSO-d_6.
Figure S26. The positive HR-ESI-MS spectrum of compound **7**.
Figure S27. ^{1}H NMR (600 MHz) spectrum of compound **8** in DMSO-d_6.
Figure S28. ^{13}C NMR (151 MHz) spectrum of compound **8** in DMSO-d_6.
Figure S29. The positive HR-ESI-MS spectrum of compound **8**.
Figure S30. ^{1}H NMR (600 MHz) spectrum of compound **9** in DMSO-d_6.
Figure S31. ^{13}C NMR (151 MHz) spectrum of compound **9** in DMSO-d_6.
Figure S32. The positive HR-ESI-MS spectrum of compound **9**.
Figure S33. ^{1}H NMR (600 MHz) spectrum of compound **10** in DMSO-d_6.
Figure S34. ^{13}C NMR (151 MHz) spectrum of compound **10** in DMSO-d_6.
Figure S35. The positive HR-ESI-MS spectrum of compound **10**.
Figure S36. ^{1}H NMR (600 MHz) spectrum of compound **11** in DMSO-d_6.
Figure S37. ^{13}C NMR (151 MHz) spectrum of compound **11** in DMSO-d_6.
Figure S38. The positive HR-ESI-MS spectrum of compound **11**.
Figure S39. ^{1}H NMR (600 MHz) spectrum of compound **12** in DMSO-d_6.
Figure S40. ^{13}C NMR (151 MHz) spectrum of compound **12** in DMSO-d_6.
Figure S41. The positive HR-ESI-MS spectrum of compound **12**.
Figure S42. ^{1}H NMR (600 MHz) spectrum of compound **13** in DMSO-d_6.

Figure S43. ^{13}C NMR (151 MHz) spectrum of compound **13** in DMSO-d_6.
Figure S44. The positive HR-ESI-MS spectrum of compound **13**.
Figure S45. ^{1}H NMR (600 MHz) spectrum of compound **14** in DMSO-d_6.
Figure S46. ^{13}C NMR (151 MHz) spectrum of compound **14** in DMSO-d_6.
Figure S47. The negative HR-ESI-MS spectrum of compound **14**.
Figure S48. ^{1}H NMR (600 MHz) spectrum of compound **15** in DMSO-d_6.
Figure S49. ^{13}C NMR (151 MHz) spectrum of compound **15** in DMSO-d_6.
Figure S50. The negative HR-ESI-MS spectrum of compound **15**.
Figure S51. ^{1}H NMR (600 MHz) spectrum of compound **16** in DMSO-d_6.
Figure S52. ^{13}C NMR (151 MHz) spectrum of compound **16** in DMSO-d_6.
Figure S53. The positive HR-ESI-MS spectrum of compound **16**.
Figure S54. ^{1}H NMR (600 MHz) spectrum of compound **17** in DMSO-d_6.
Figure S55. ^{13}C NMR (151 MHz) spectrum of compound **17** in DMSO-d_6.
Figure S56. The positive HR-ESI-MS spectrum of compound **17**.
Figure S57. ^{1}H NMR (600 MHz) spectrum of compound **18** in DMSO-d_6.
Figure S58. ^{13}C NMR (151 MHz) spectrum of compound **18** in DMSO-d_6.
Figure S59. The positive HR-ESI-MS spectrum of compound **18**.
Figure S60. ^{1}H NMR (600 MHz) spectrum of compound **19** in DMSO-d_6.
Figure S61. ^{13}C NMR (151 MHz) spectrum of compound **19** in DMSO-d_6.
Figure S62. The positive HR-ESI-MS spectrum of compound **19**.
Figure S63. ^{1}H NMR (600 MHz) spectrum of compound **20** in DMSO-d_6.
Figure S64. ^{13}C NMR (151 MHz) spectrum of compound **20** in DMSO-d_6.
Figure S65. The negative HR-ESI-MS spectrum of compound **20**.
Figure S66. ^{1}H NMR (600 MHz) spectrum of compound **21** in DMSO-d_6.
Figure S67. ^{13}C NMR (151 MHz) spectrum of compound **21** in DMSO-d_6.
Figure S68. The positive HR-ESI-MS spectrum of compound **21**.
Figure S69. ^{1}H NMR (600 MHz) spectrum of compound **22** in DMSO-d_6.
Figure S70. ^{13}C NMR (151 MHz) spectrum of compound **22** in DMSO-d_6.
Figure S71. The negative HR-ESI-MS spectrum of compound **22**.
Figure S72. ^{1}H NMR (600 MHz) spectrum of compound **23** in DMSO-d_6.
Figure S73. ^{13}C NMR (151 MHz) spectrum of compound **23** in DMSO-d_6.
Figure S74. The positive HR-ESI-MS spectrum of compound **23**.
Figure S75. ^{1}H NMR (600 MHz) spectrum of compound **24** in DMSO-d_6.
Figure S76. ^{13}C NMR (151 MHz) spectrum of compound **24** in DMSO-d_6.
Figure S77. The positive HR-ESI-MS spectrum of compound **24**.
Figure S78. ^{1}H NMR (600 MHz) spectrum of compound **25** in CD_3OD.
Figure S79. ^{13}C NMR (151 MHz) spectrum of compound **25** in CD_3OD.
Figure S80. The positive HR-ESI-MS spectrum of compound **25**.
Figure S81. ^{1}H NMR (600 MHz) spectrum of compound **26** in DMSO-d_6.
Figure S82. ^{13}C NMR (151 MHz) spectrum of compound **26** in DMSO-d_6.
Figure S83. The positive HR-ESI-MS spectrum of compound **26**.
Figure S84. ^{1}H NMR (600 MHz) spectrum of compound **27** in DMSO-d_6.
Figure S85. ^{13}C NMR (151 MHz) spectrum of compound **27** in DMSO-d_6.
Figure S86. The positive HR-ESI-MS spectrum of compound **27**.

Figure S87. ^{1}H NMR (600 MHz) spectrum of compound **28** in DMSO-d_6.
Figure S88. ^{13}C NMR (151 MHz) spectrum of compound **28** in DMSO-d_6.
Figure S89. The positive HR-ESI-MS spectrum of compound **28**.
Figure S90. ^{1}H NMR (600 MHz) spectrum of compound **29** in DMSO-d_6.
Figure S91. ^{13}C NMR (151 MHz) spectrum of compound **29** in DMSO-d_6.
Figure S92. The positive HR-ESI-MS spectrum of compound **29**.
Figure S93. Gibbs free energy and equilibrium populations of low-energy conformers of **1** in ECD calculations.

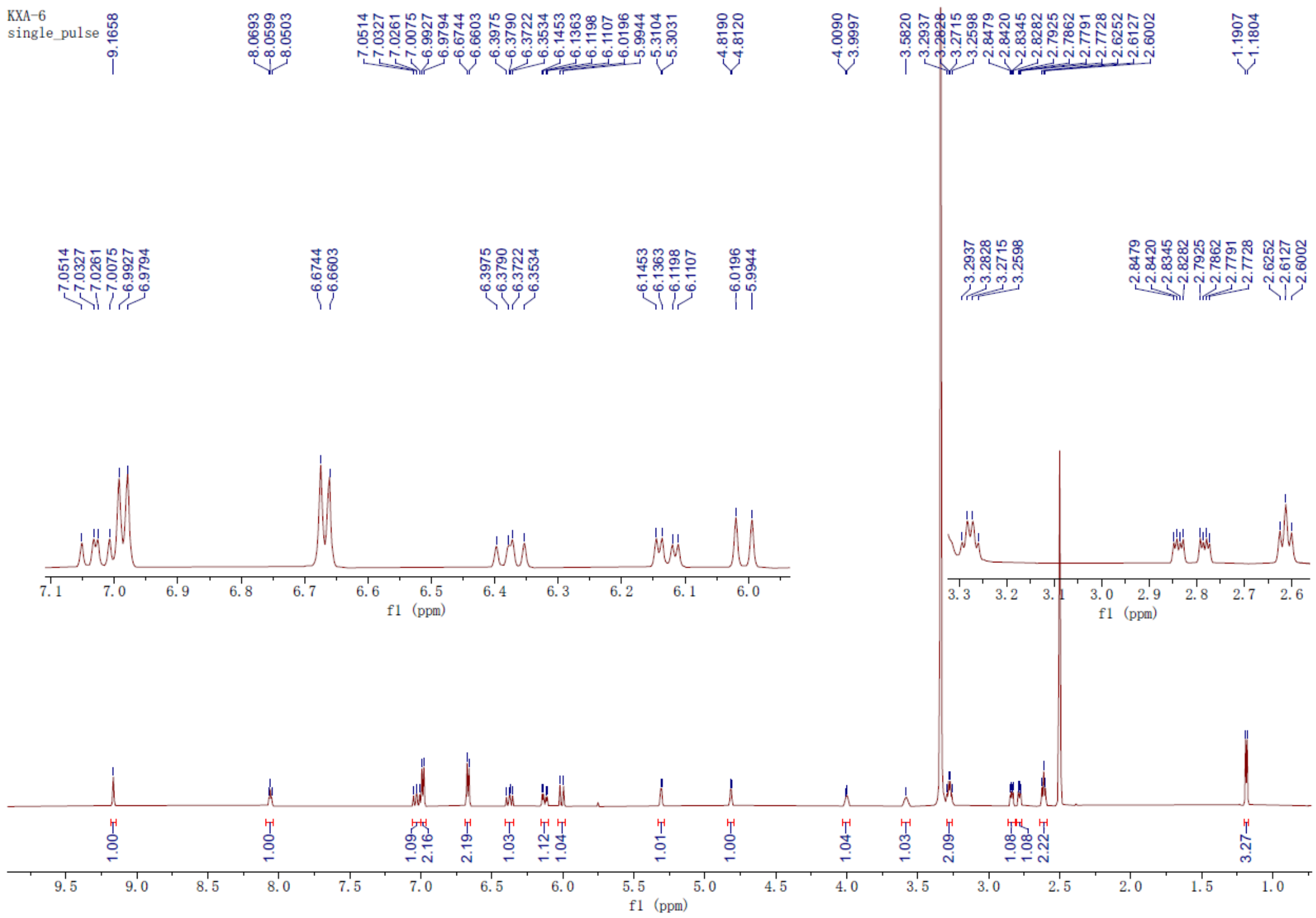

Figure S1. ^{1}H NMR (600 MHz) spectrum of compound **1** in DMSO-d_6.

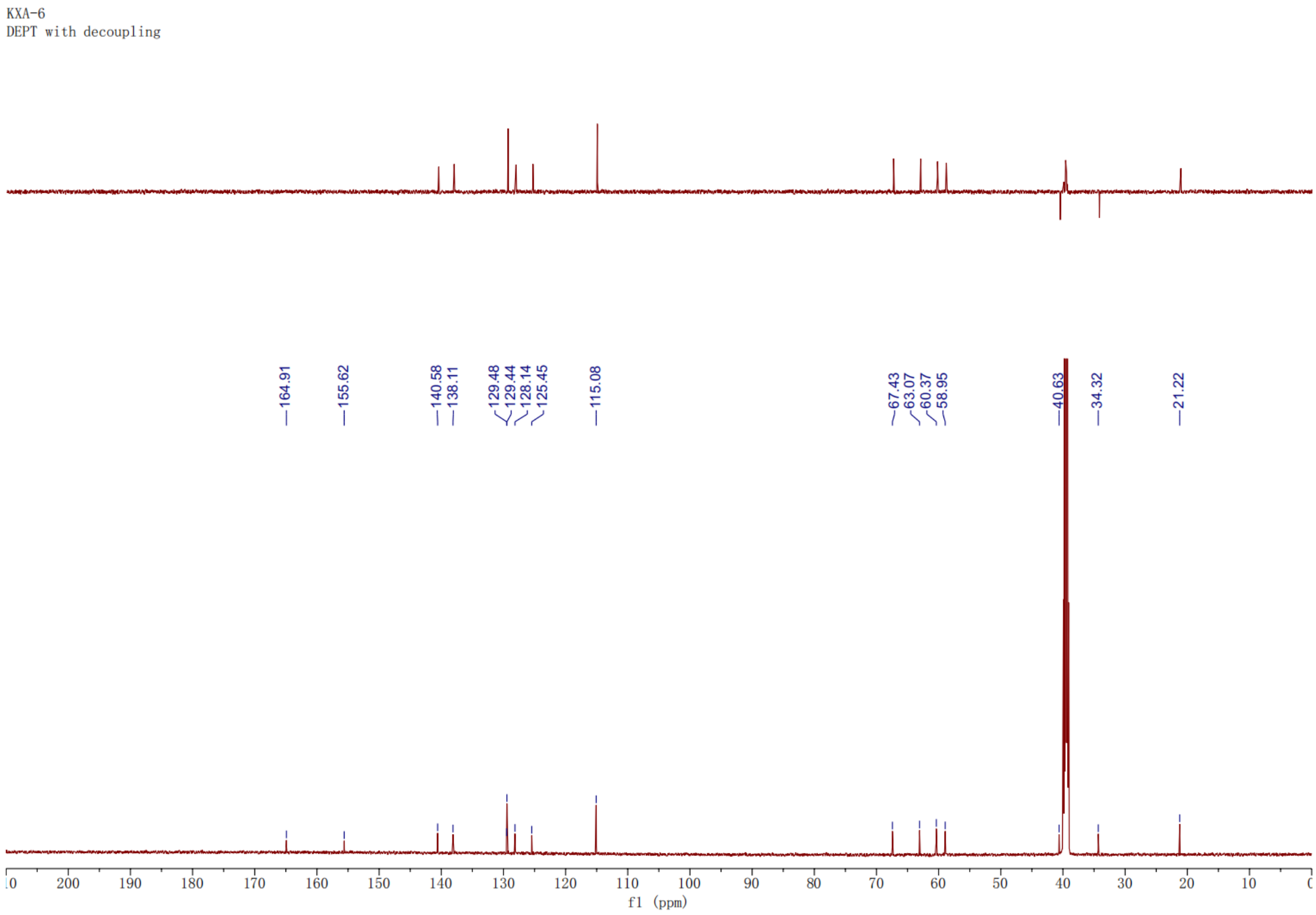

Figure S2. ^{13}C NMR (151 MHz) spectrum of compound **1** in DMSO-d_6.

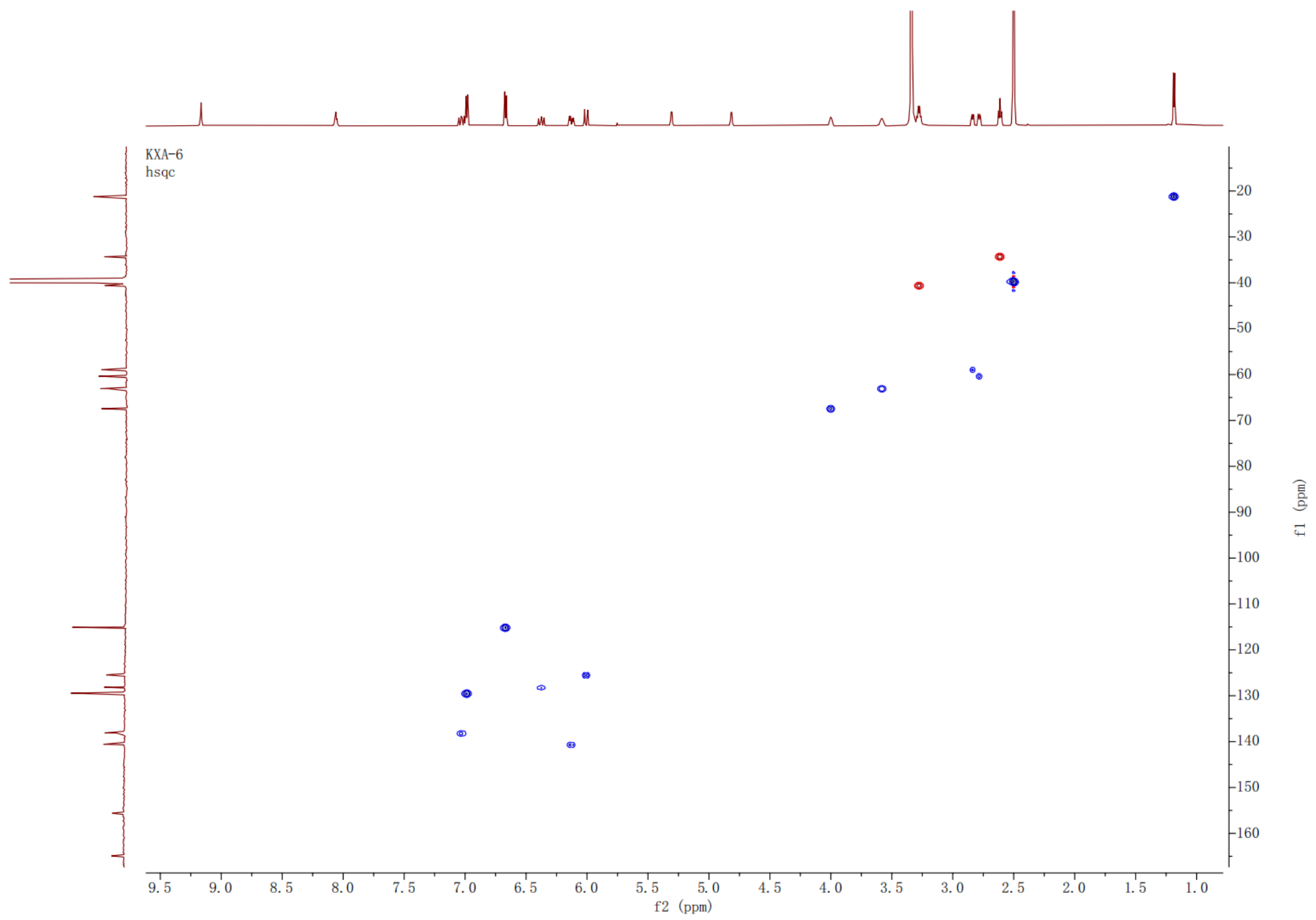

Figure S3. HSQC spectrum of compound **1** in DMSO-*d*6.

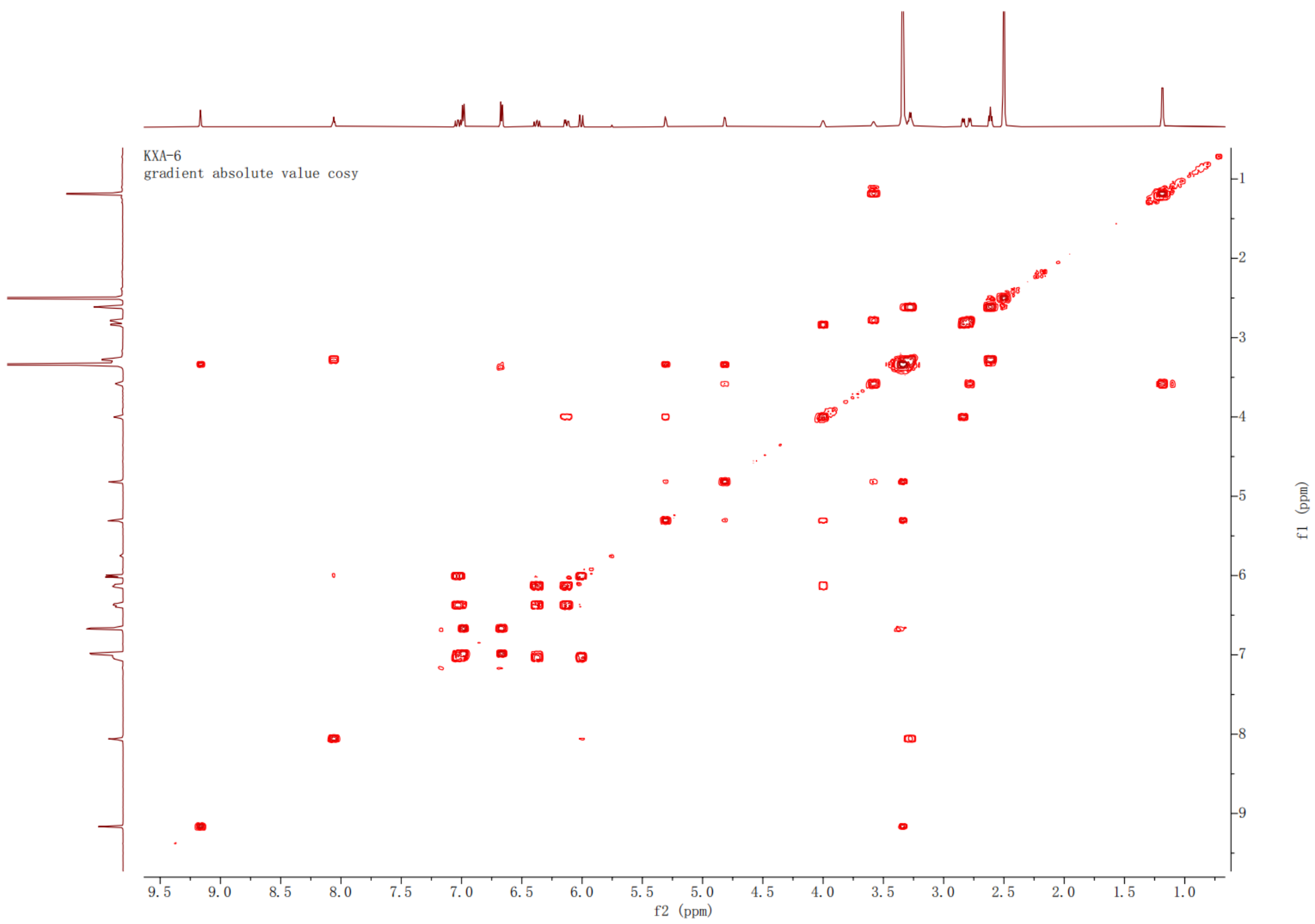

Figure S4. ^{1}H-^{1}H COSY spectrum of compound **1** in DMSO-*d*6.

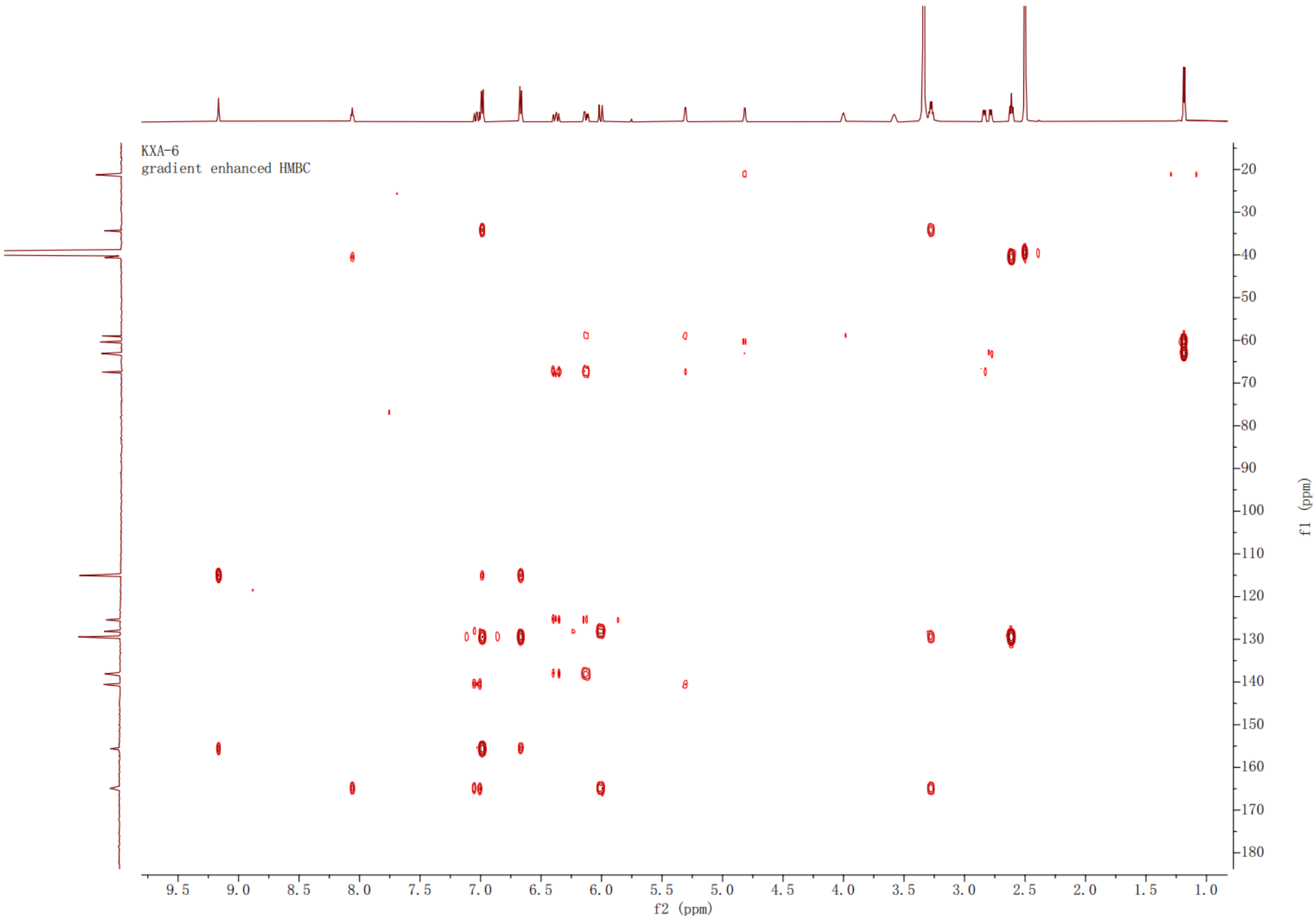

Figure S5. HMBC spectrum of compound **1** in DMSO-d_6.

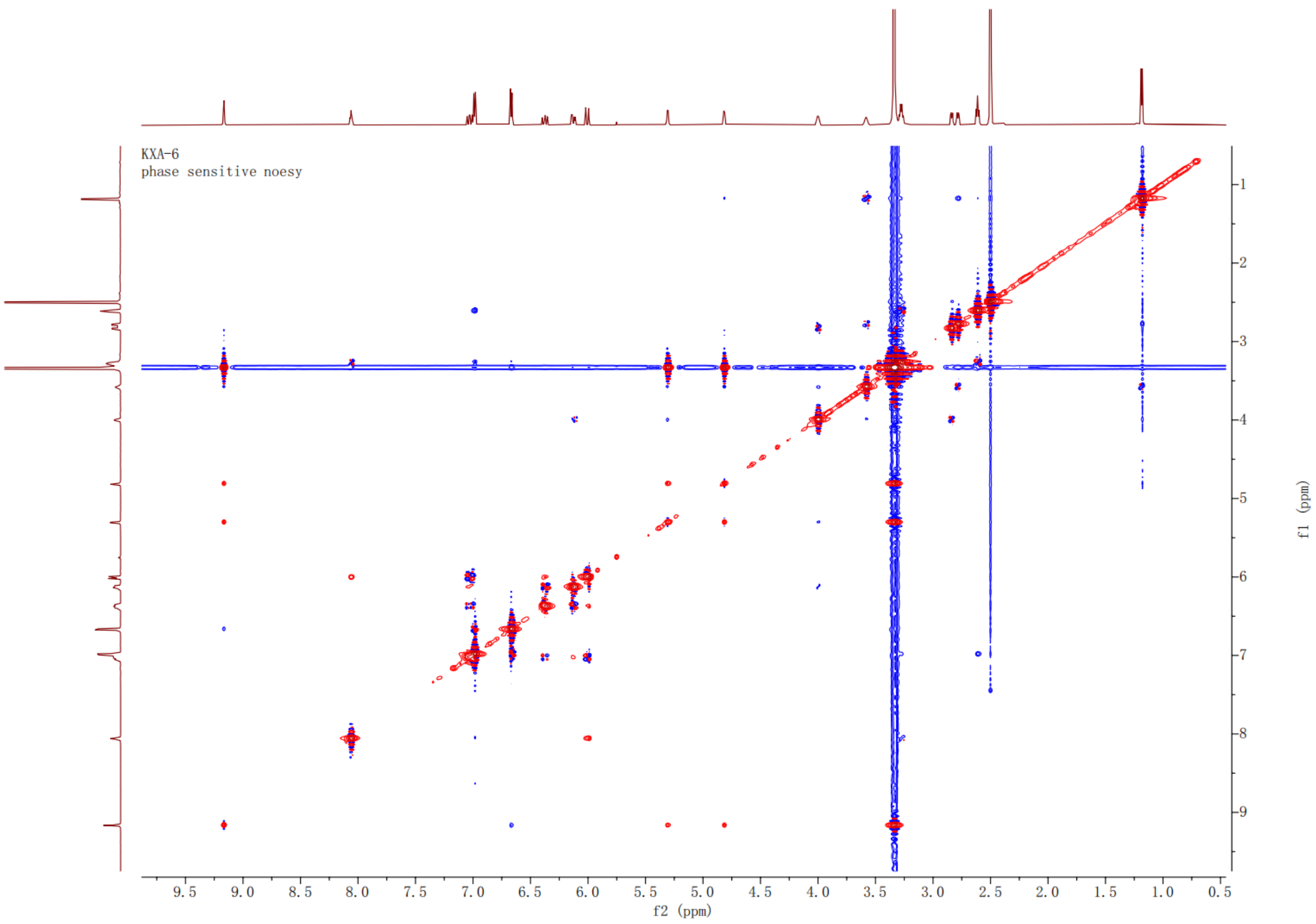

Figure S6. NOESY spectrum of compound **1** in DMSO-d_6.

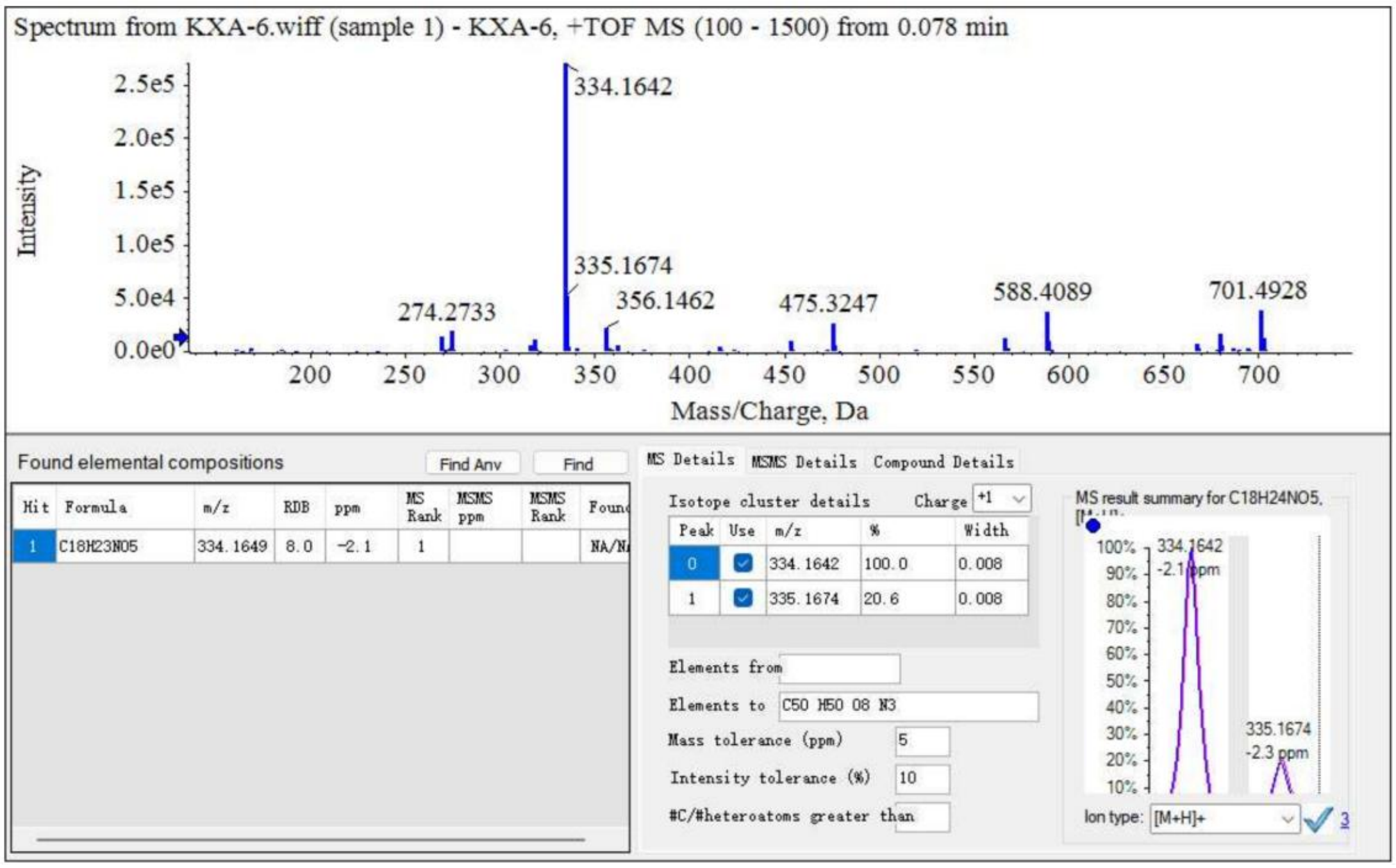

Figure S7. The positive HR-ESI-MS spectrum of compound **1**.

Anton Paar GmbH
Anton Paar Strasse 10
8054 Graz
Austria

Anton Paar Polarimeter - Measurement(s)

MCP 100
Software Version: 1.50.4366.160
Serial Number: 99154608

Unique Id 700

- Sample Name: KXA-6
- Date: 10/22/2025 - 08:05 AM
- Username: Administrator
- Sample State: Ok

- Measurement Mode: Specific Rotation
- Measurement Result: 54.324 °
- Concentration: 0.370 g/100ml
- Optical Rotation: 0.201 °
- Set Temperature: 25.0 °C
- Temperature: 25.0 °C

Figure S8. Optical rotation spectrum of compound **1**.

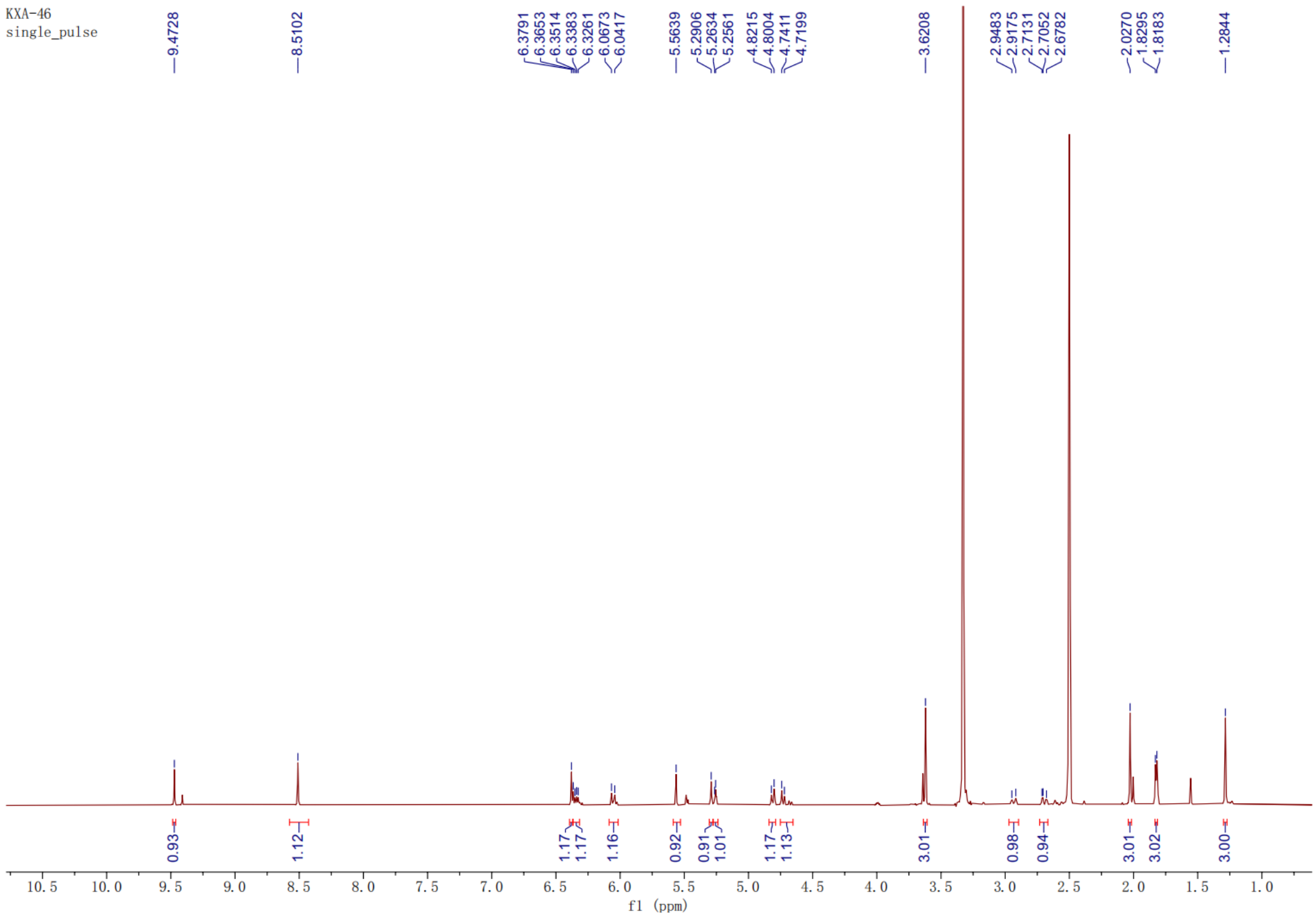

Figure S9. ^{1}H NMR (600 MHz) spectrum of compound **2** in DMSO-*d*6.

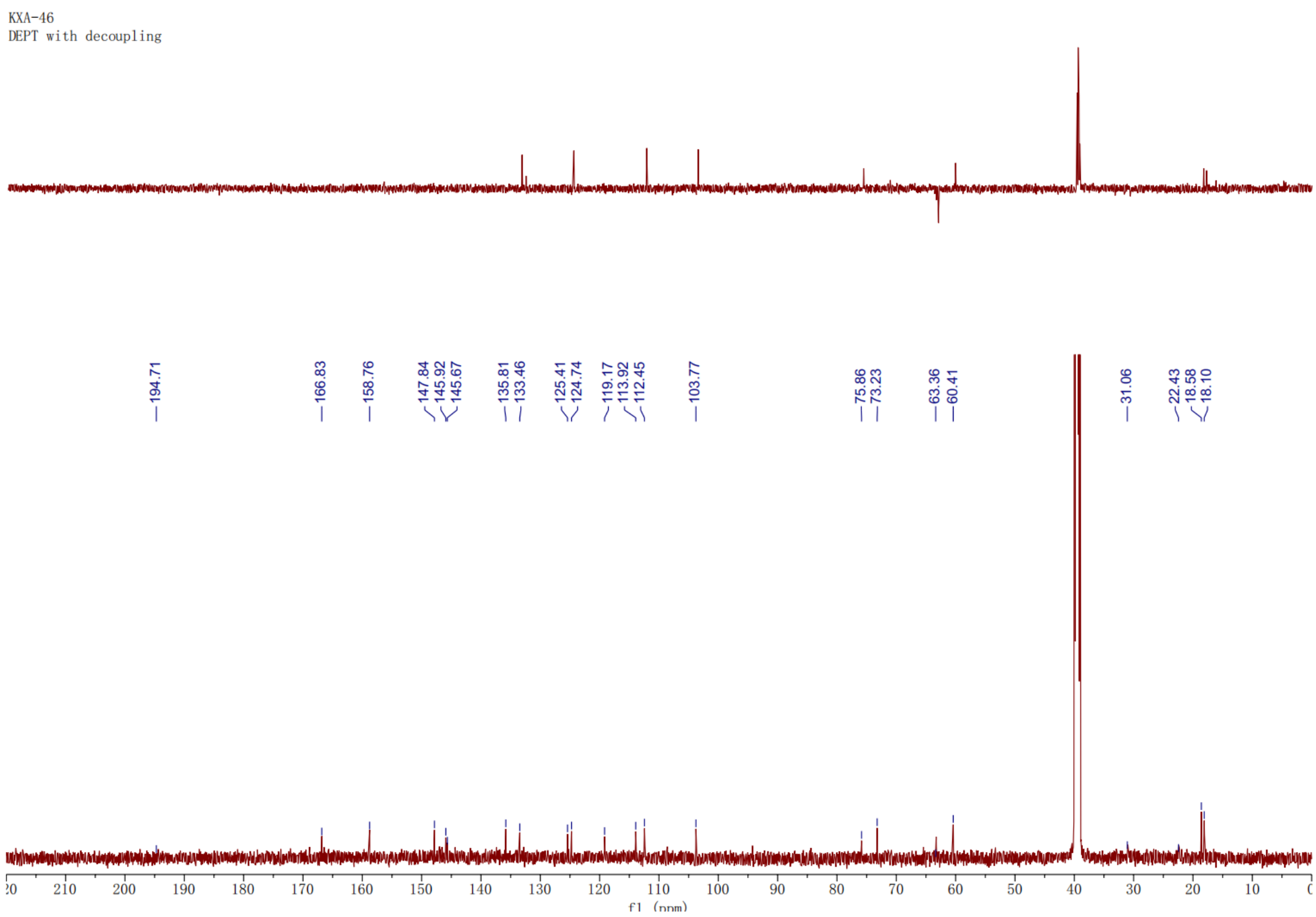

Figure S10. ^{13}C NMR (151 MHz) spectrum of compound **2** in DMSO-*d*6.

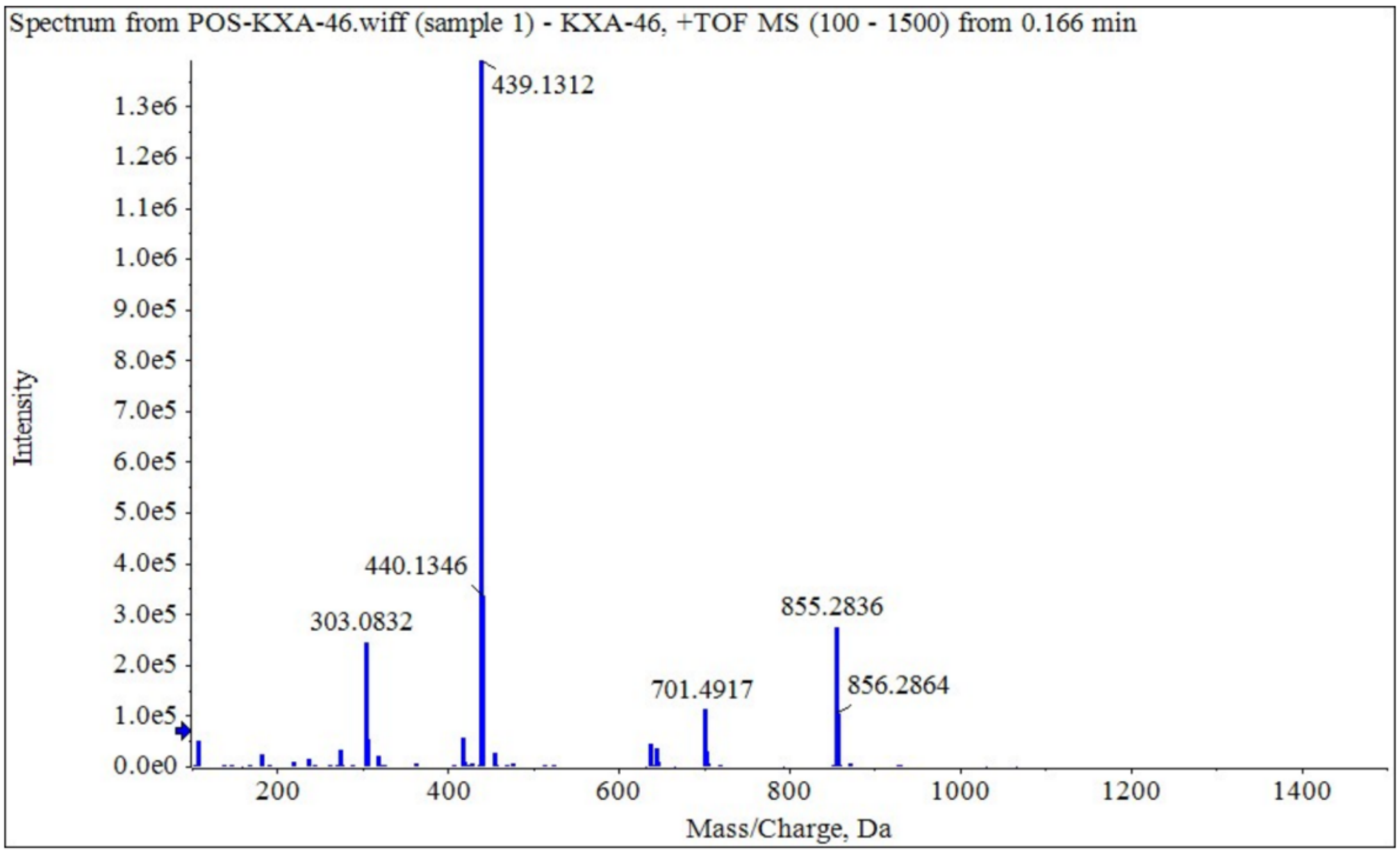

Figure S11. The positive HR-ESI-MS spectrum of compound **2**.

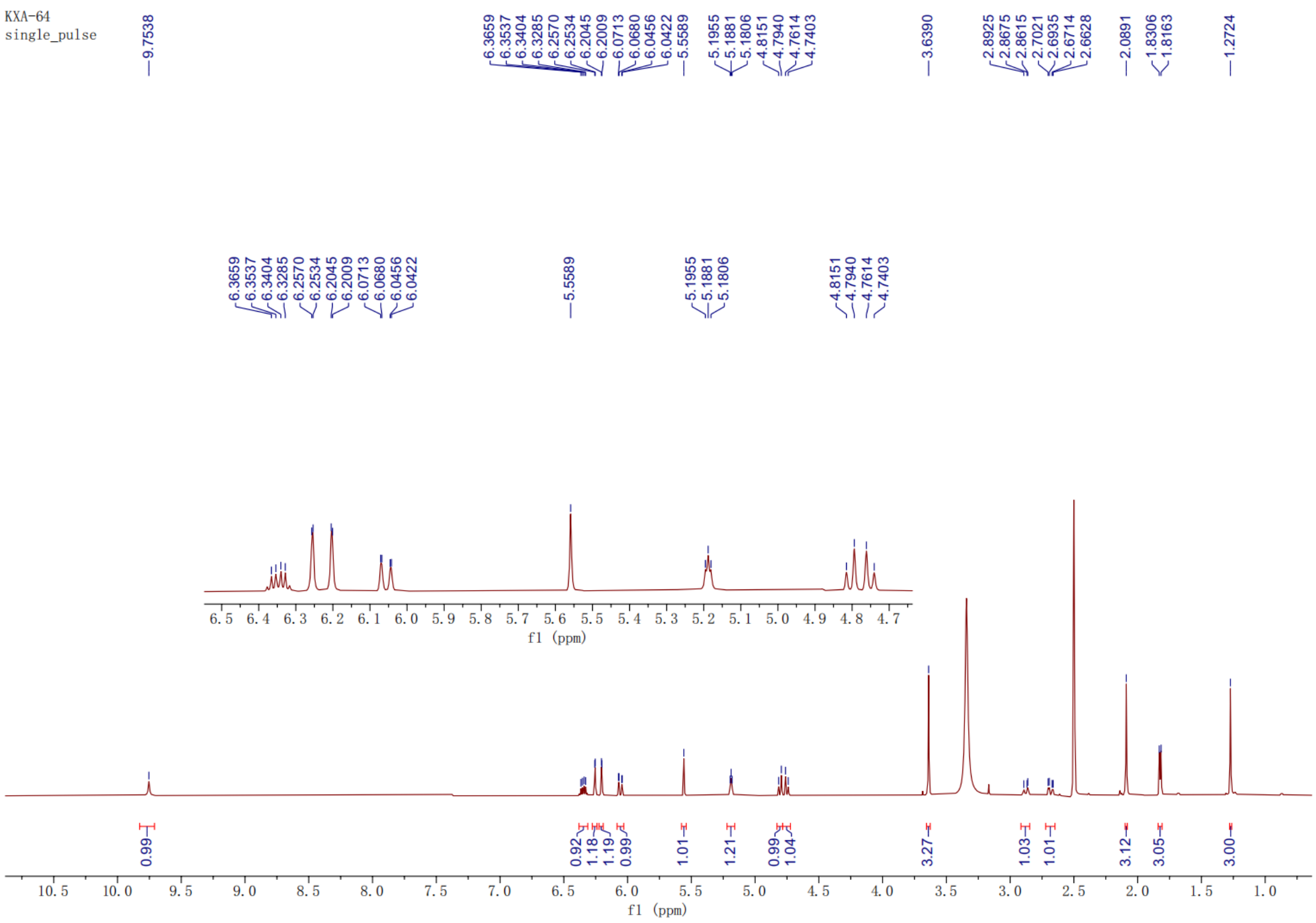

Figure S12. ^{1}H NMR (600 MHz) spectrum of compound **3** in DMSO-d_6.

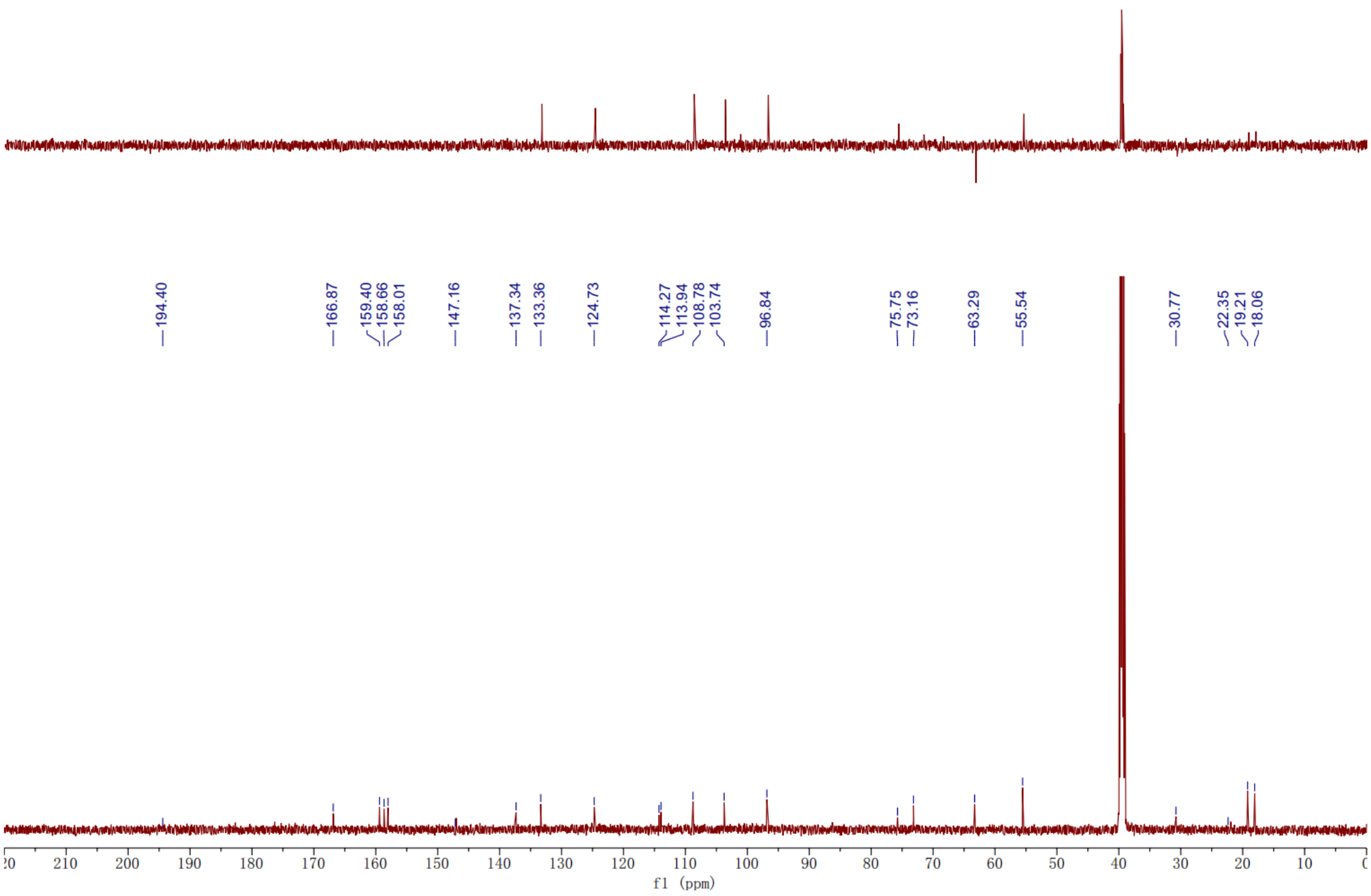

Figure S13. ^{13}C NMR (151 MHz) spectrum of compound **3** in DMSO-d_6.

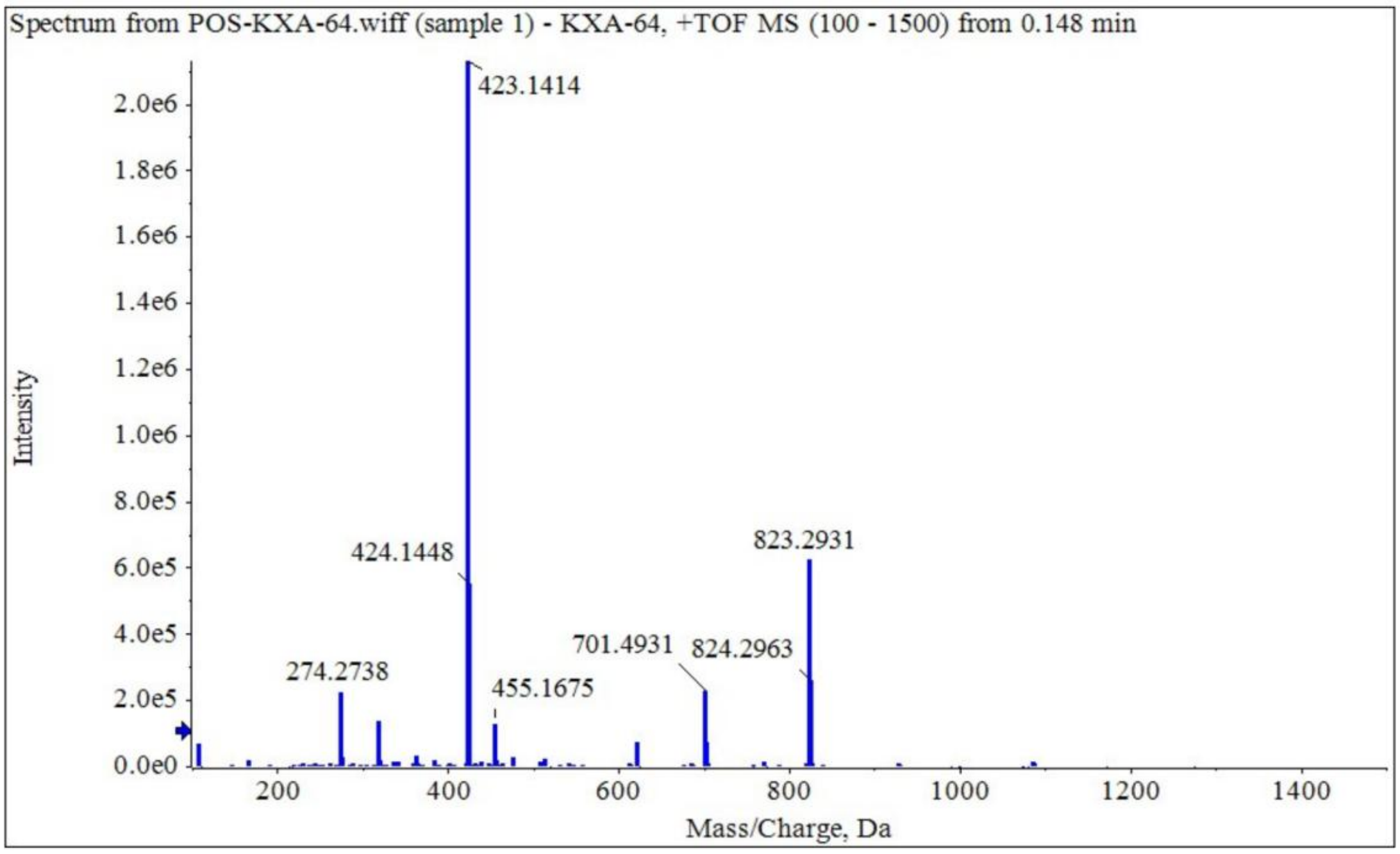

Figure S14. The positive HR-ESI-MS spectrum of compound **3**.

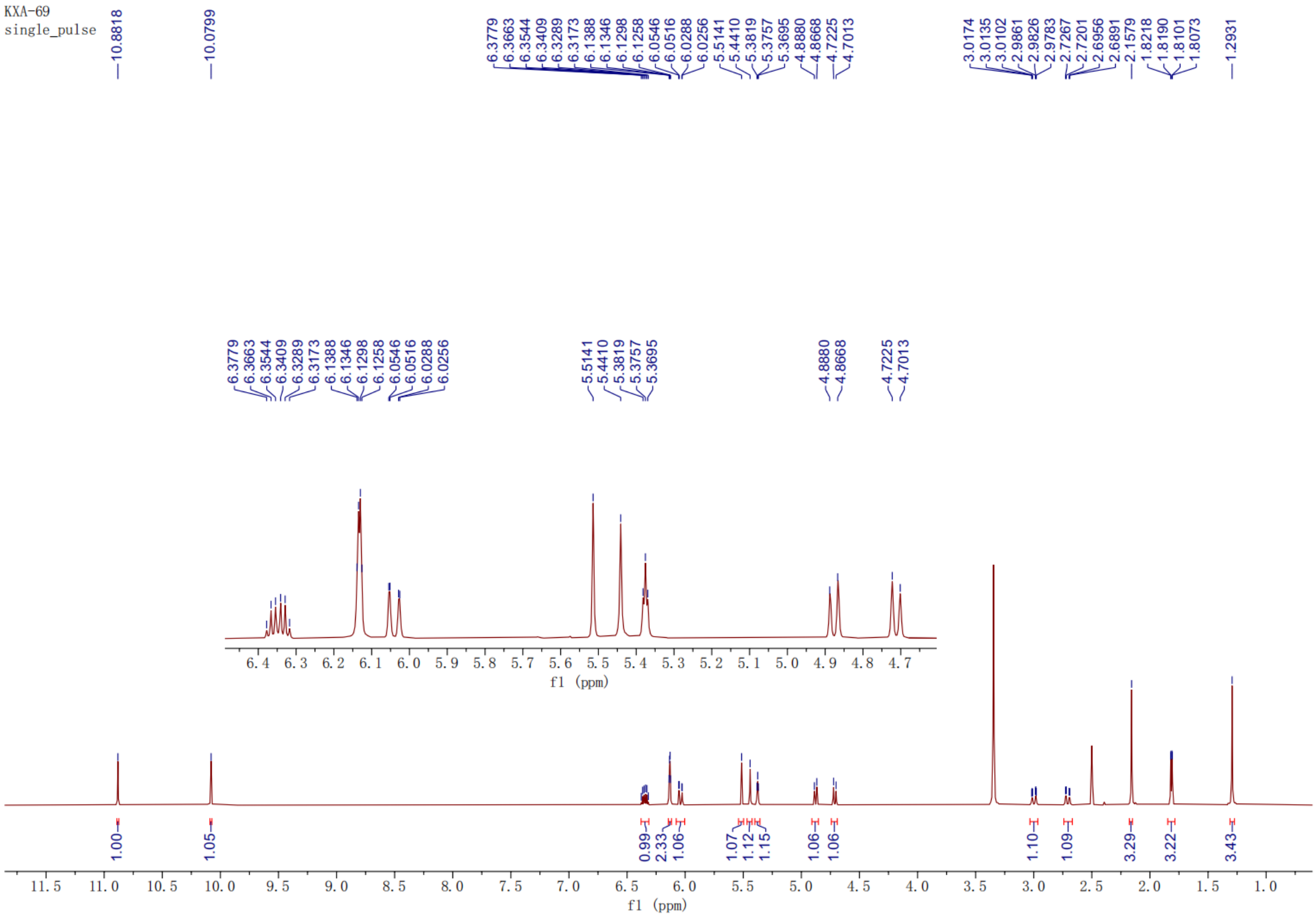

Figure S15. ^{1}H NMR (600 MHz) spectrum of compound **4** in DMSO-d_6.

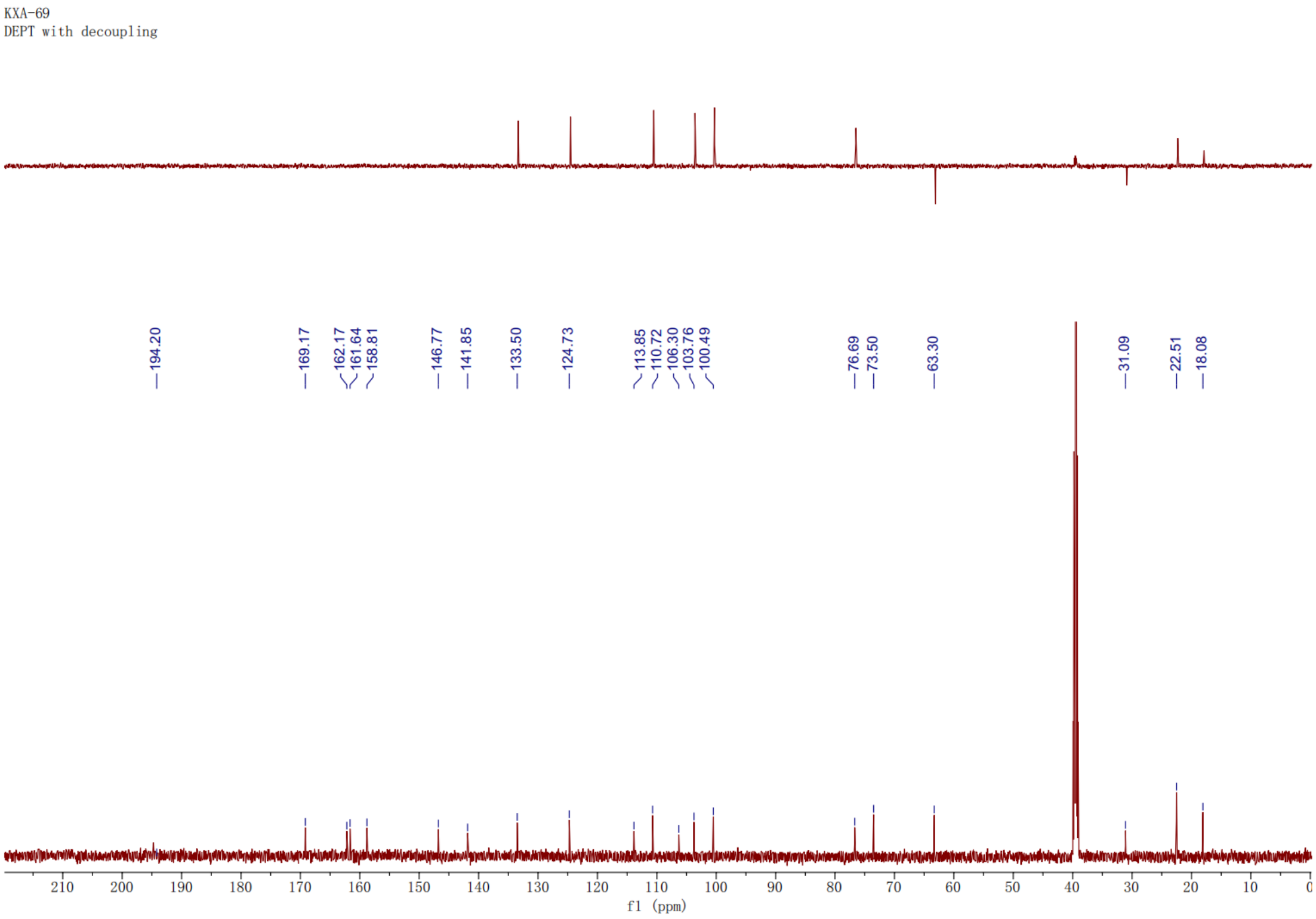

Figure S16. ^{13}C NMR (151 MHz) spectrum of compound **4** in DMSO-d_6.

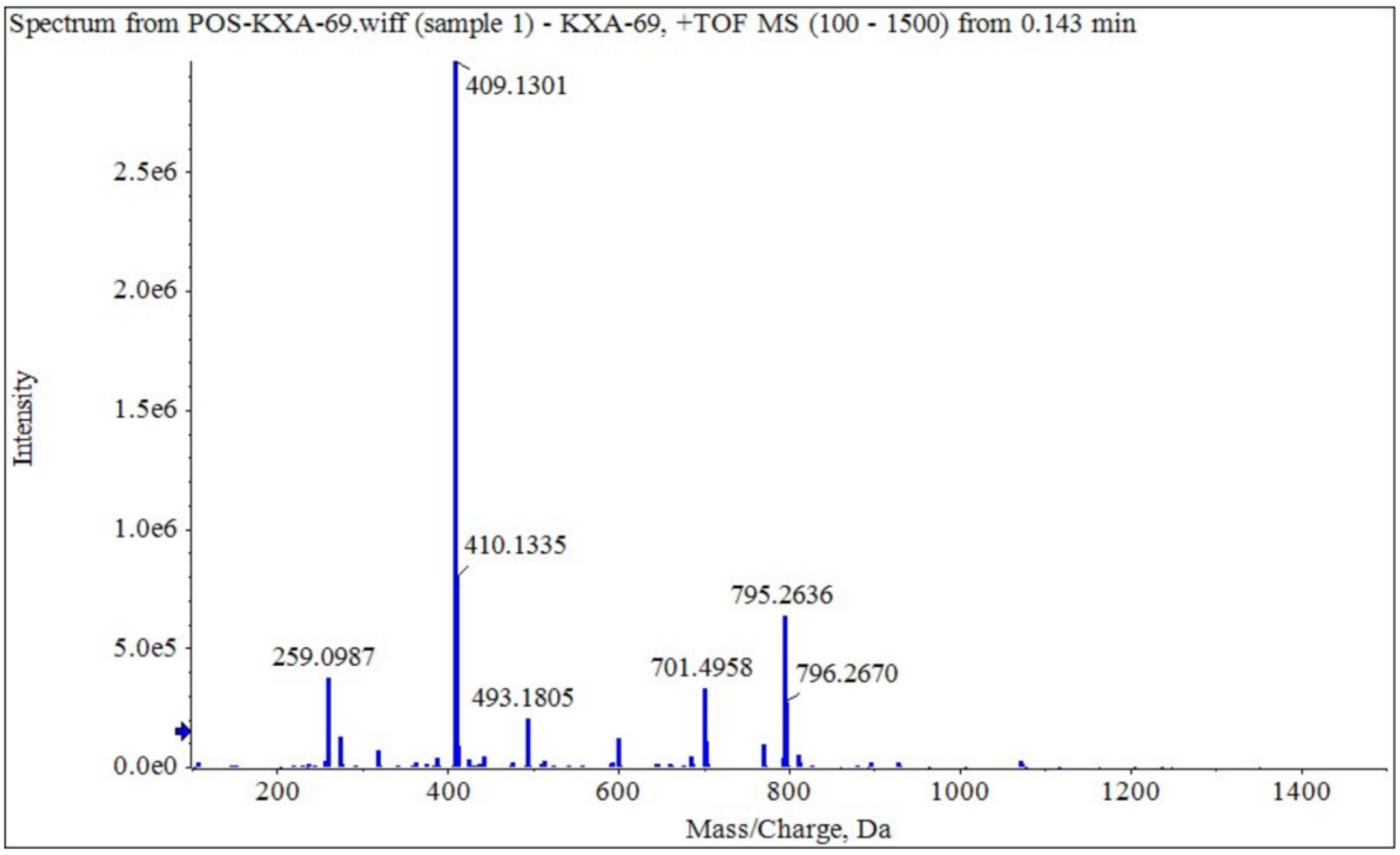

Figure S17. The positive HR-ESI-MS spectrum of compound **4**.

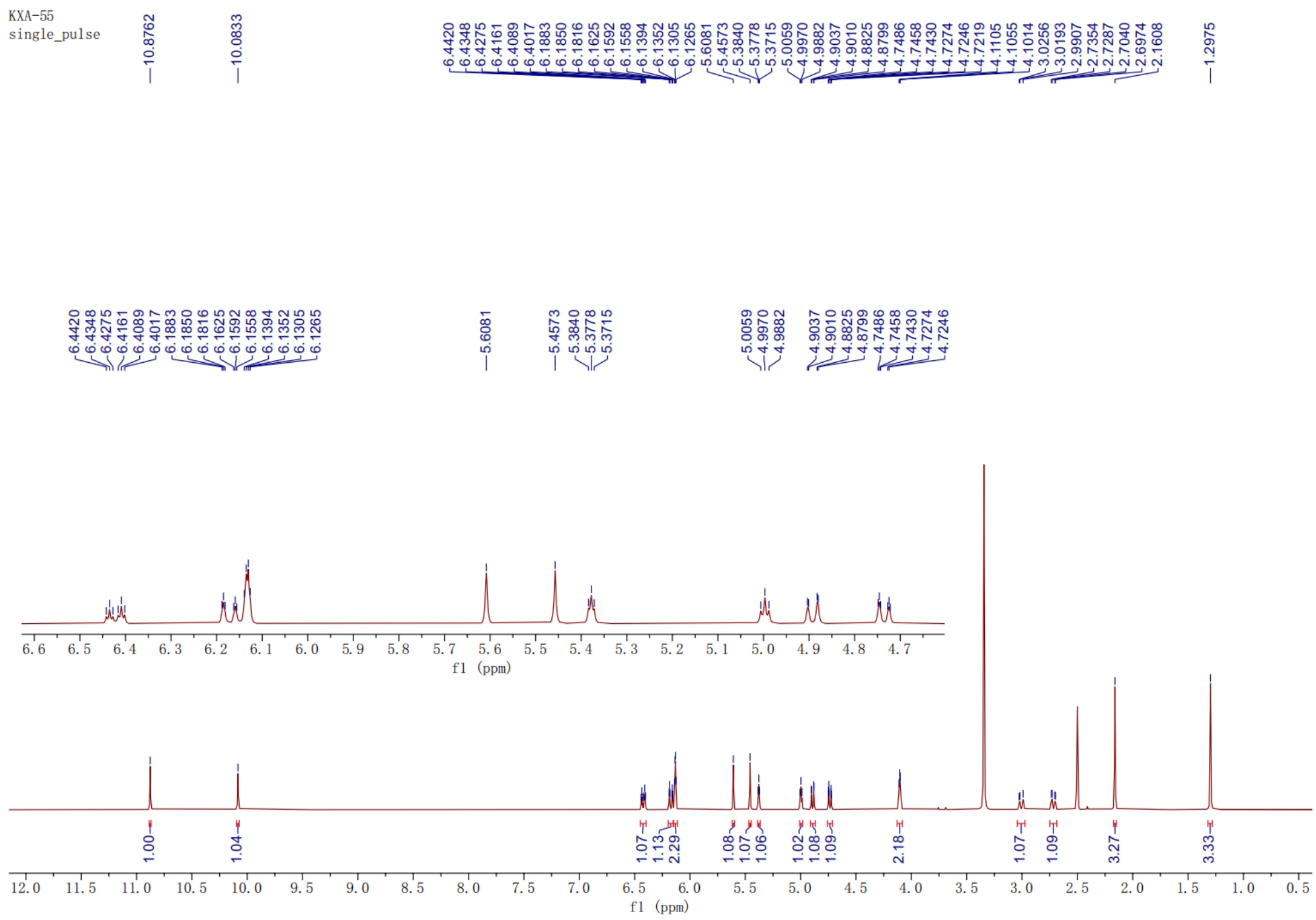

Figure S18. ^{1}H NMR (600 MHz) spectrum of compound **5** in DMSO-d_6.

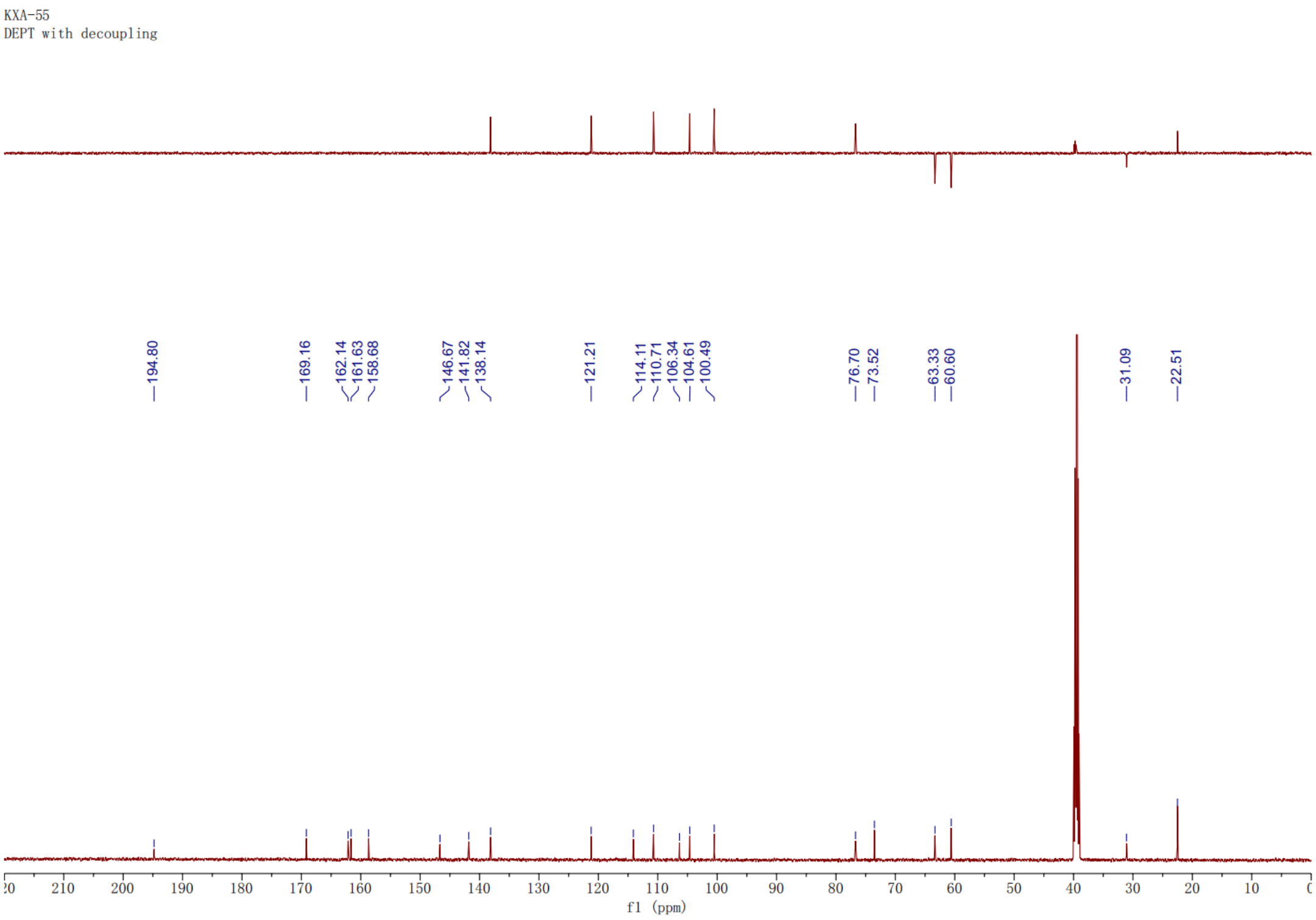

Figure S19. ^{13}C NMR (151 MHz) spectrum of compound **5** in DMSO-d_6.

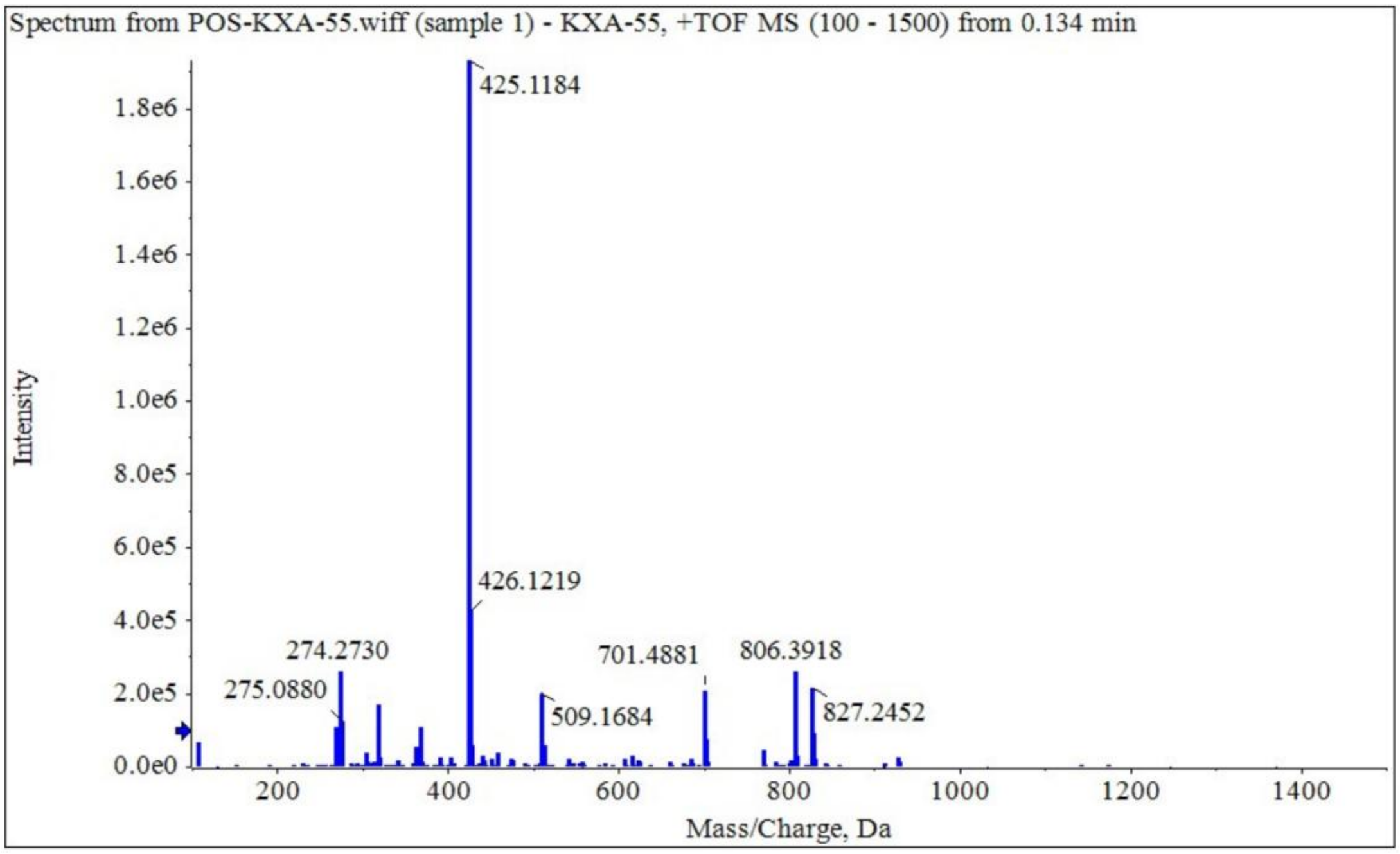

Figure S20. The positive HR-ESI-MS spectrum of compound **5**.

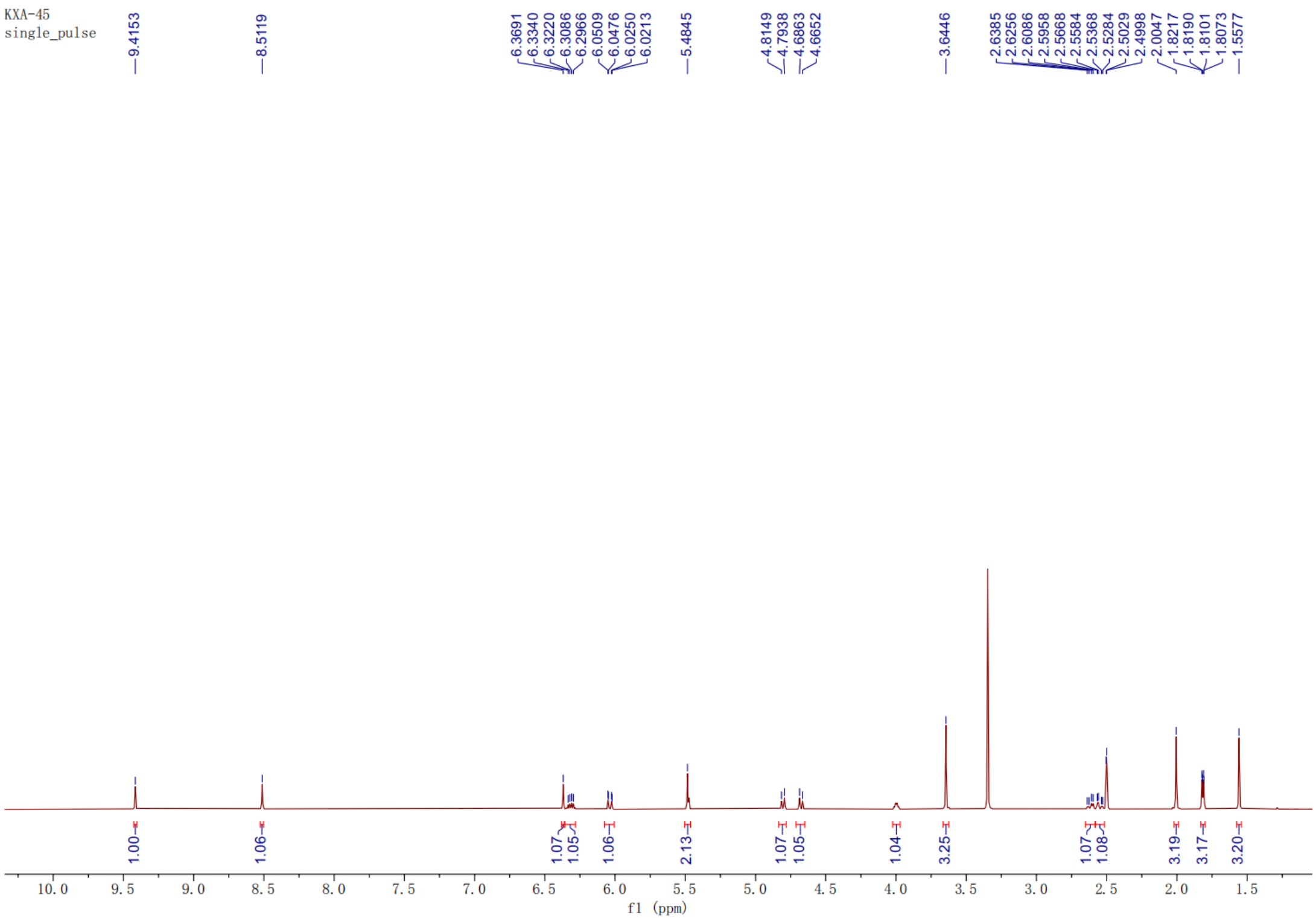

Figure S21. ^{1}H NMR (600 MHz) spectrum of compound **6** in DMSO-d_6.

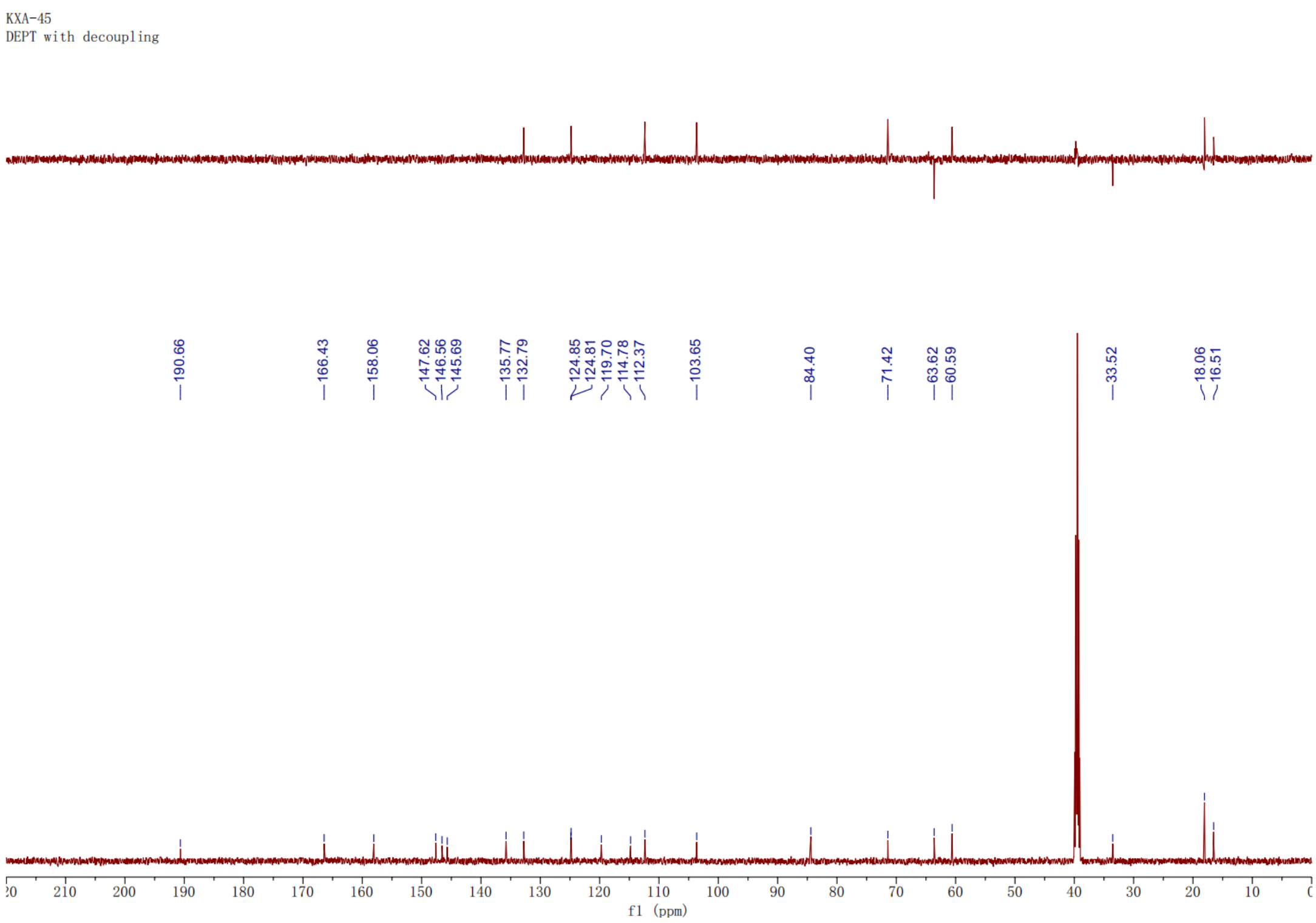

Figure S22. ^{13}C NMR (151 MHz) spectrum of compound **6** in DMSO-d_6.

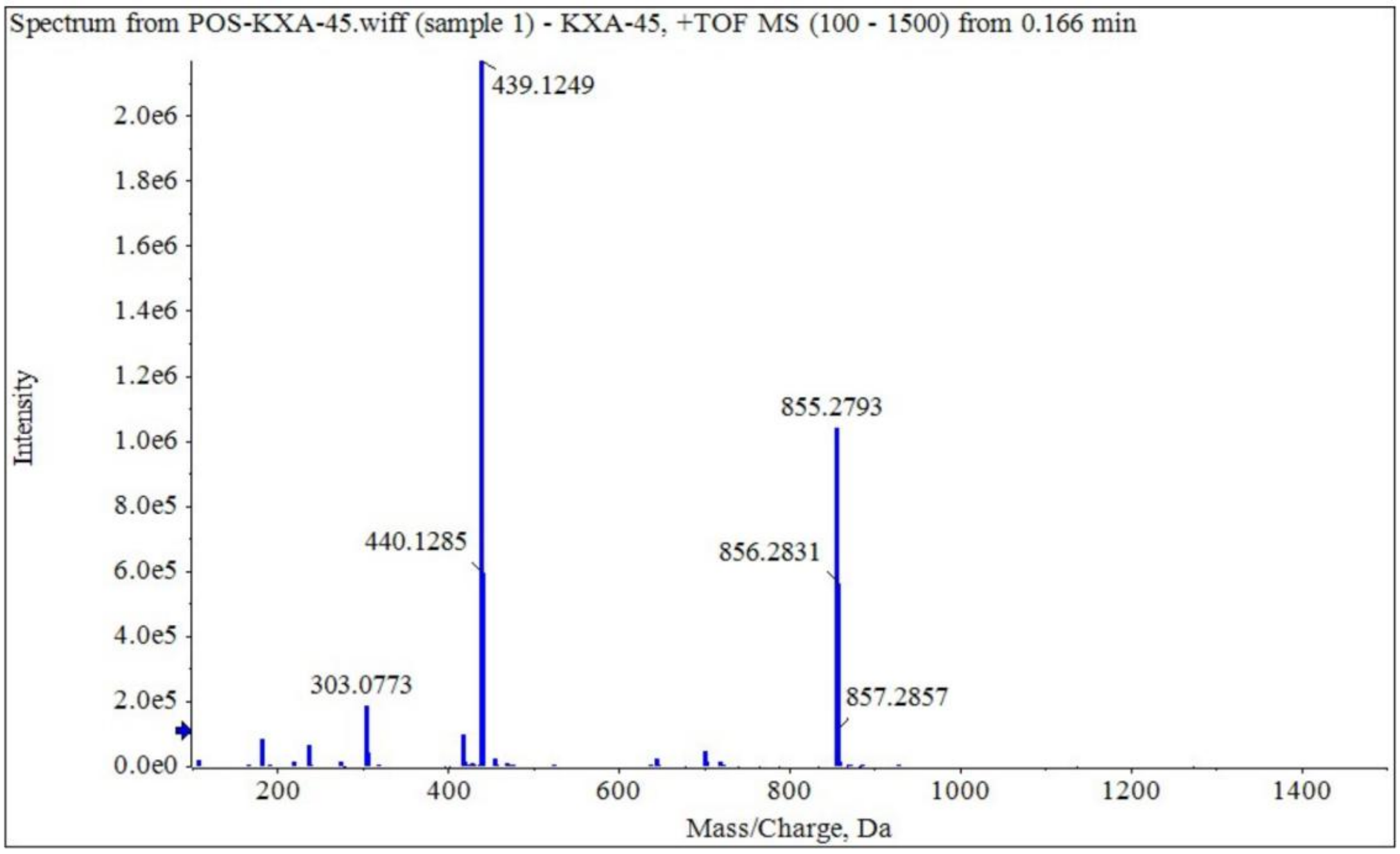

Figure S23. The positive HR-ESI-MS spectrum of compound **6**.

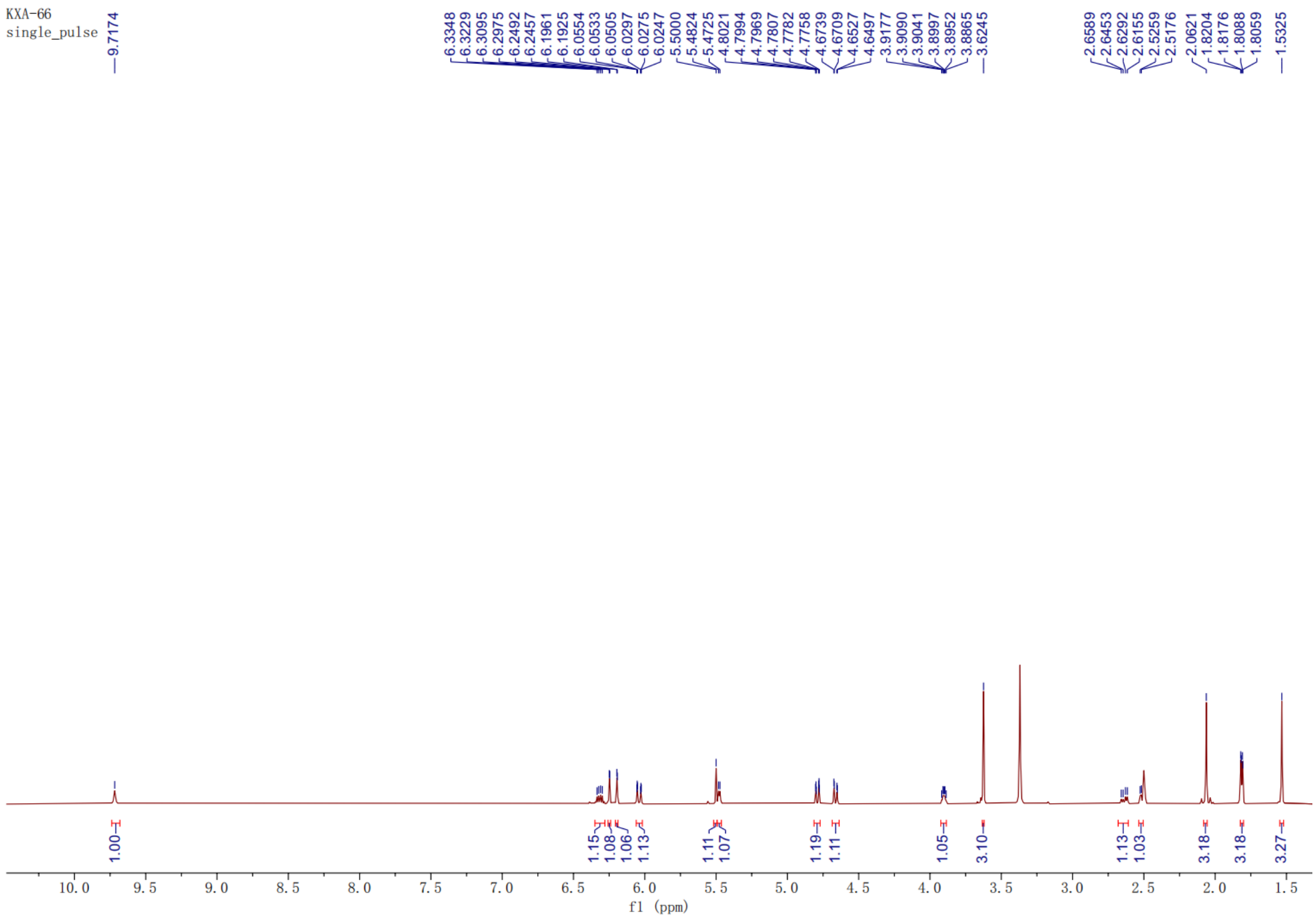

Figure S24. ^{1}H NMR (600 MHz) spectrum of compound **7** in DMSO-d_6.

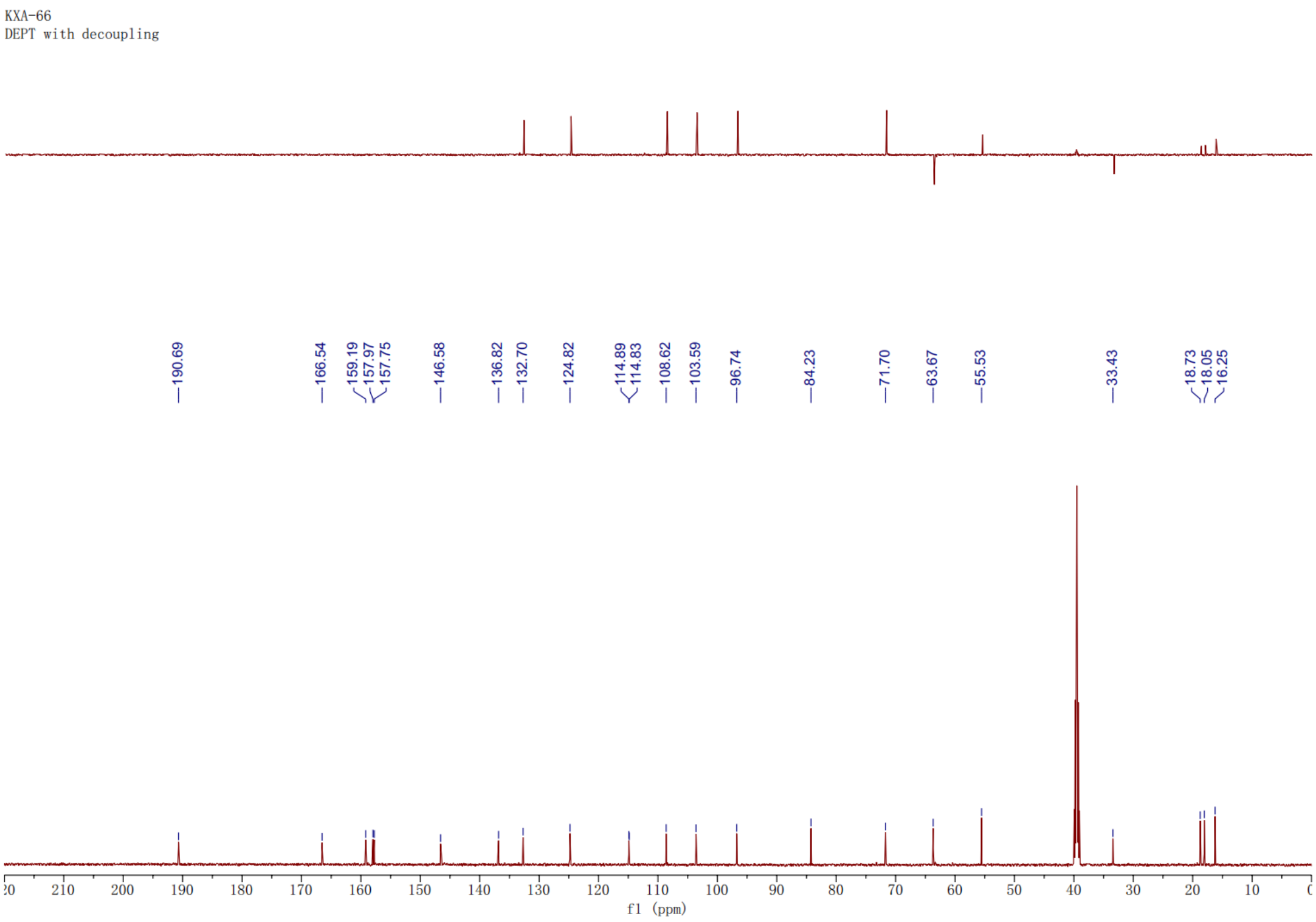

Figure S25. ^{13}C NMR (151 MHz) spectrum of compound **7** in DMSO-d_6.

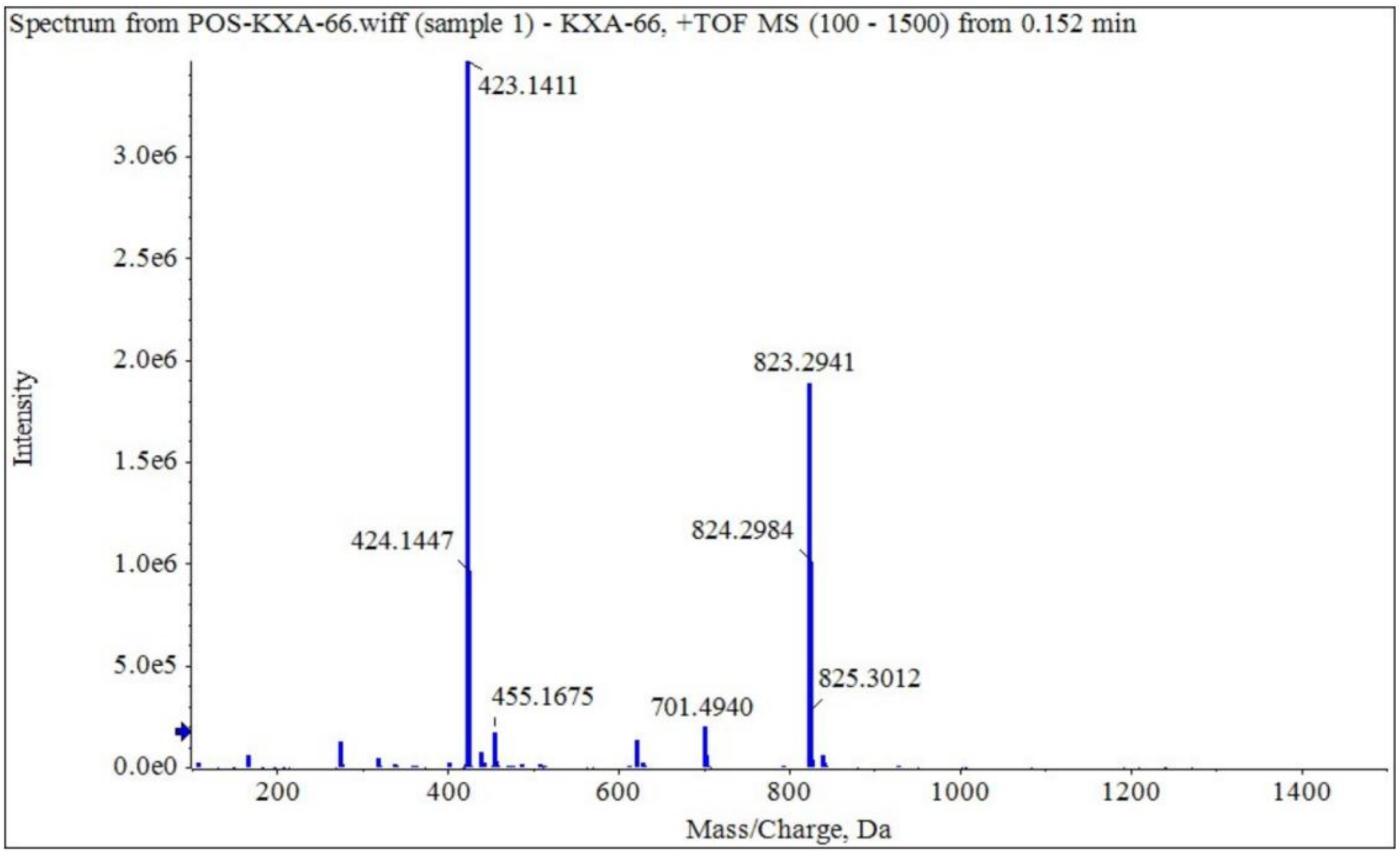

Figure S26. The positive HR-ESI-MS spectrum of compound **7**.

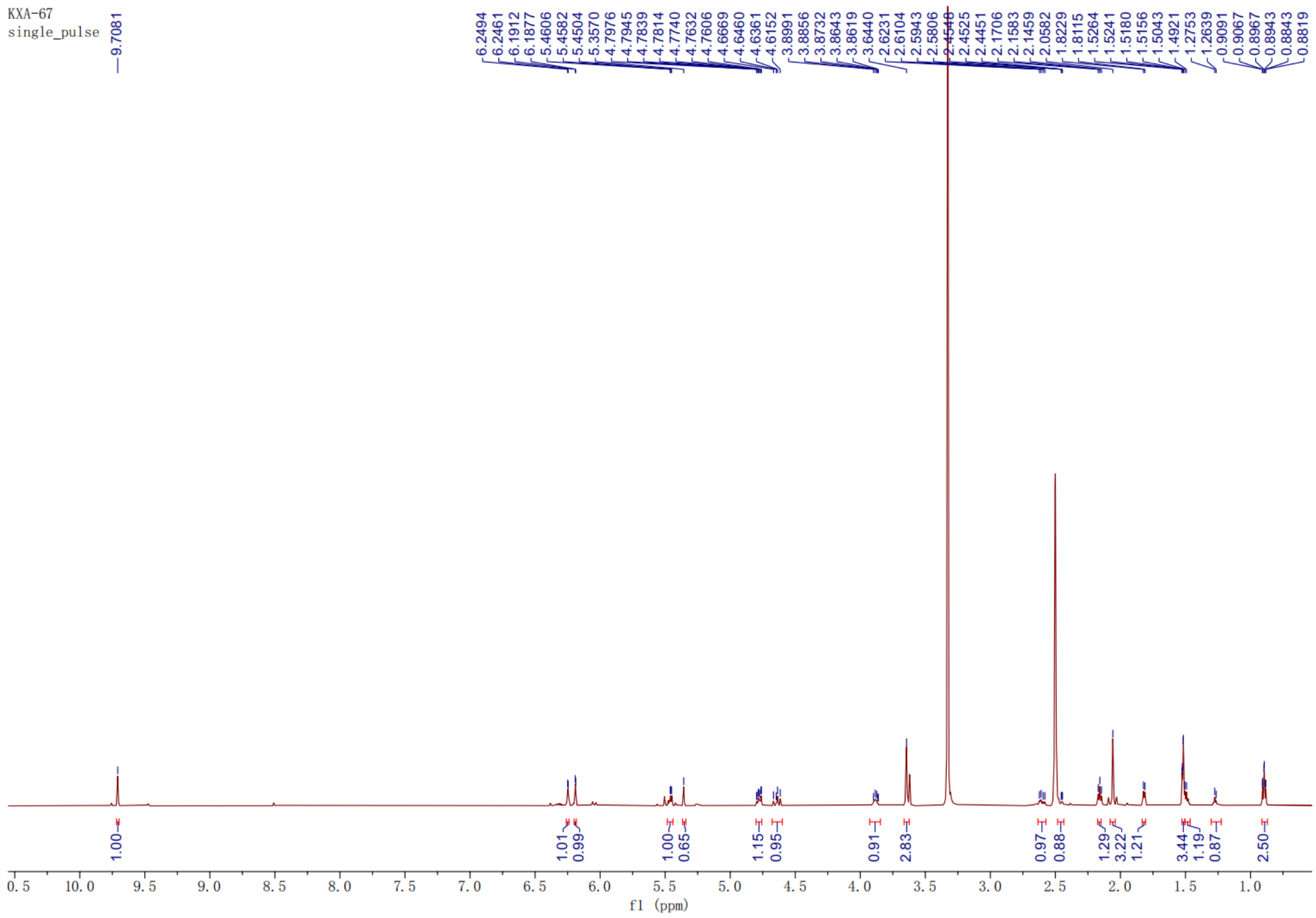

Figure S27. ^{1}H NMR (600 MHz) spectrum of compound **8** in DMSO-d_6.

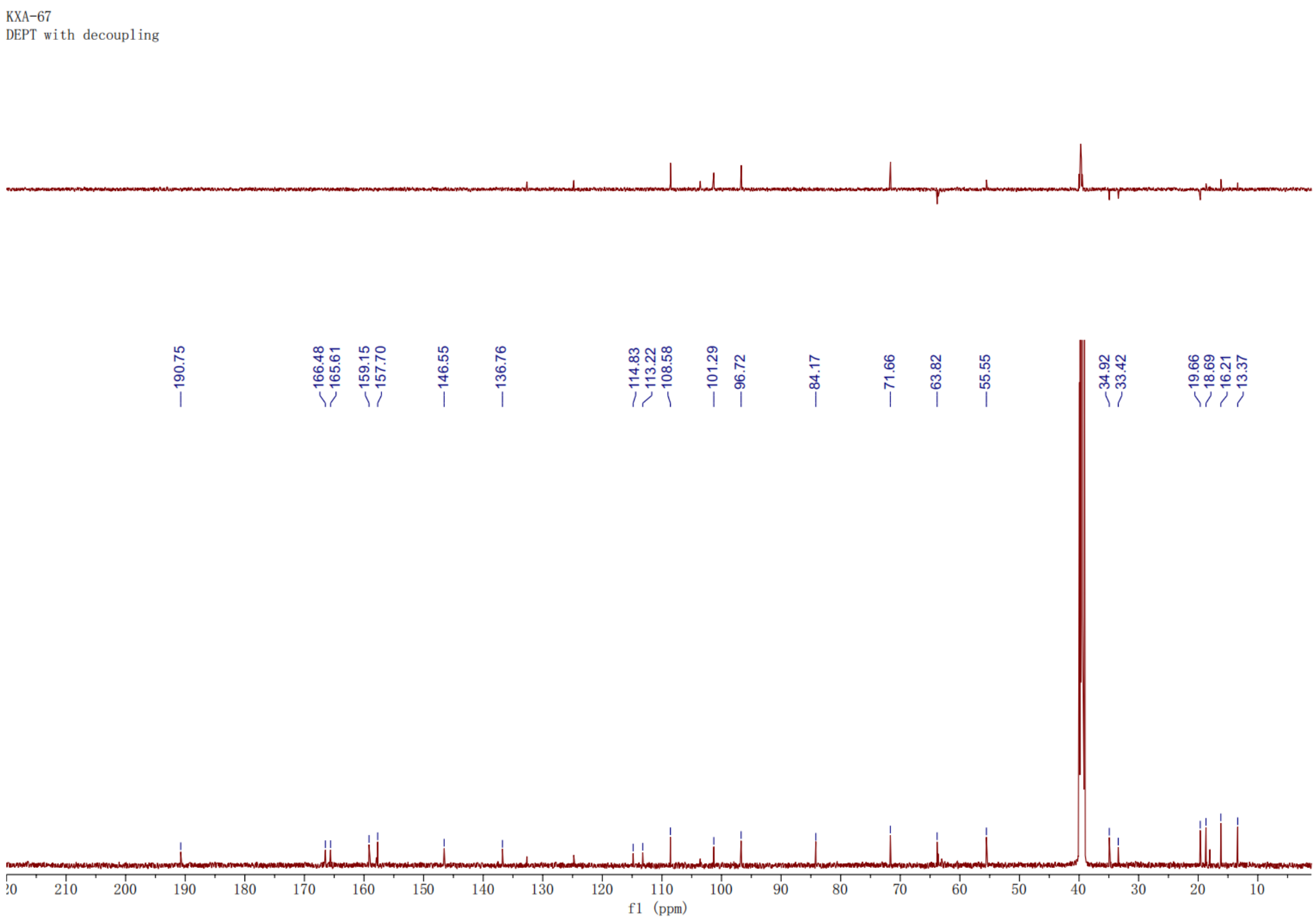

Figure S28. ^{13}C NMR (151 MHz) spectrum of compound **8** in DMSO-d_6.

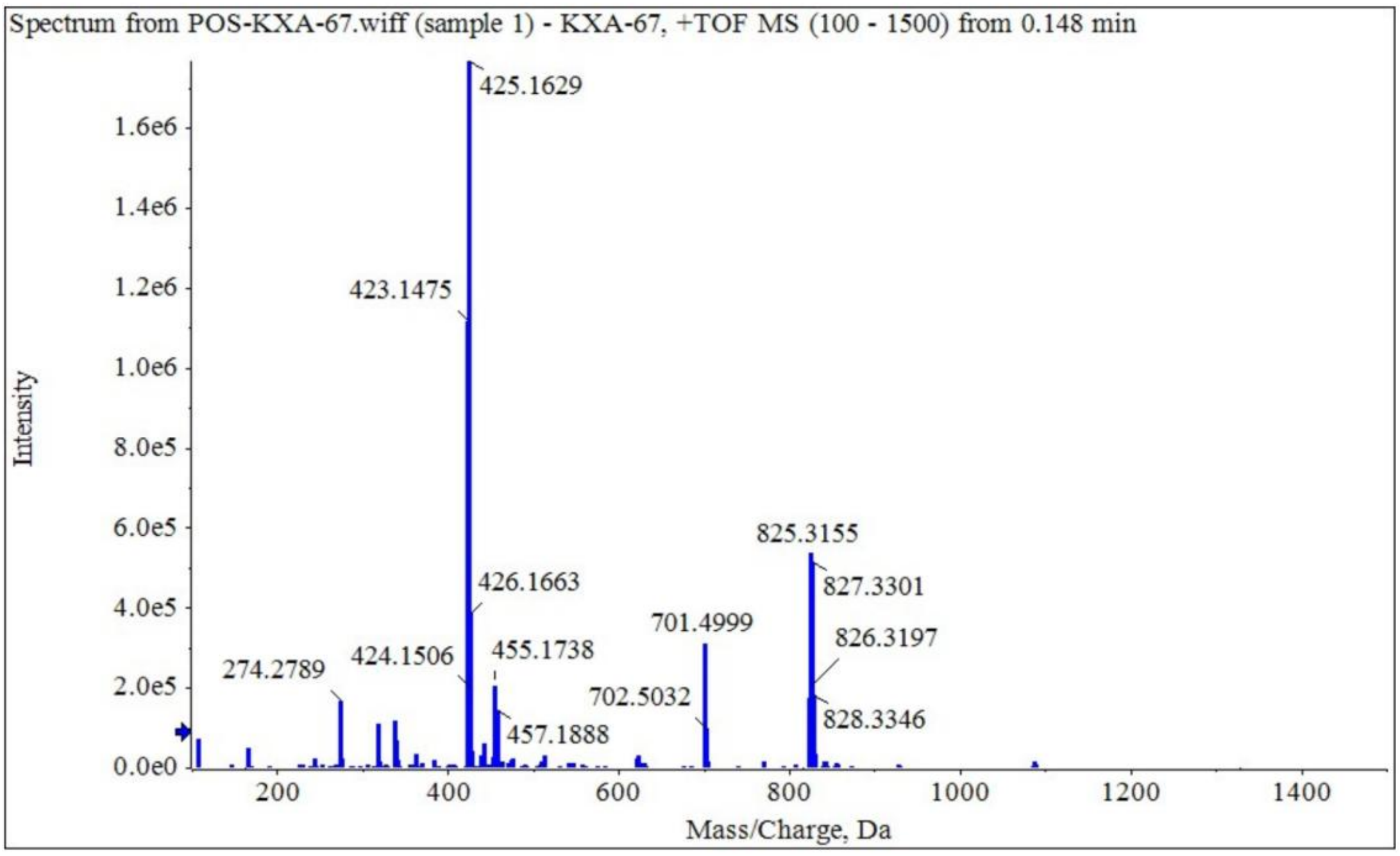

Figure S29. The positive HR-ESI-MS spectrum of compound **8**.

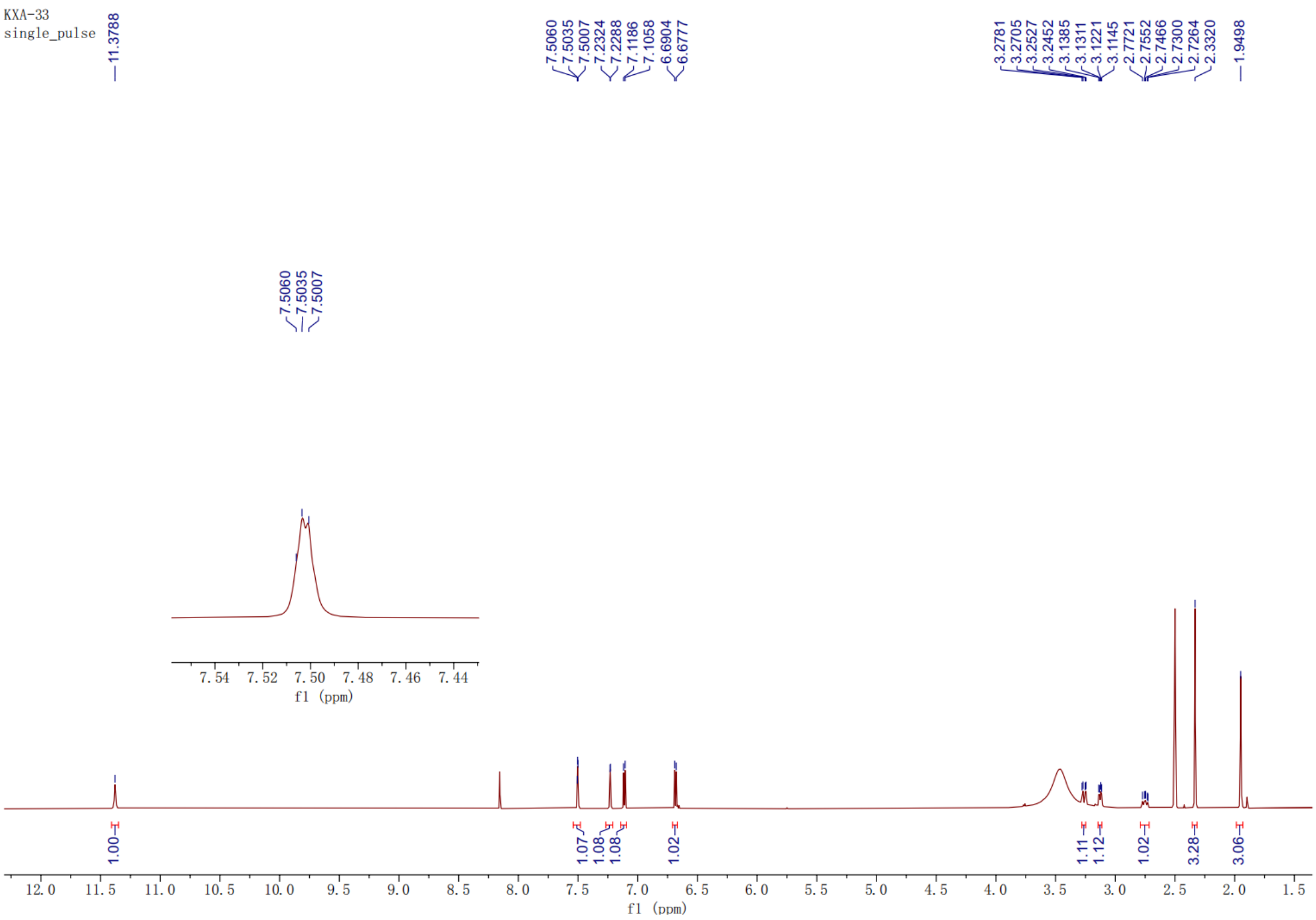

Figure S30. ^{1}H NMR (600 MHz) spectrum of compound **9** in DMSO-d_6.

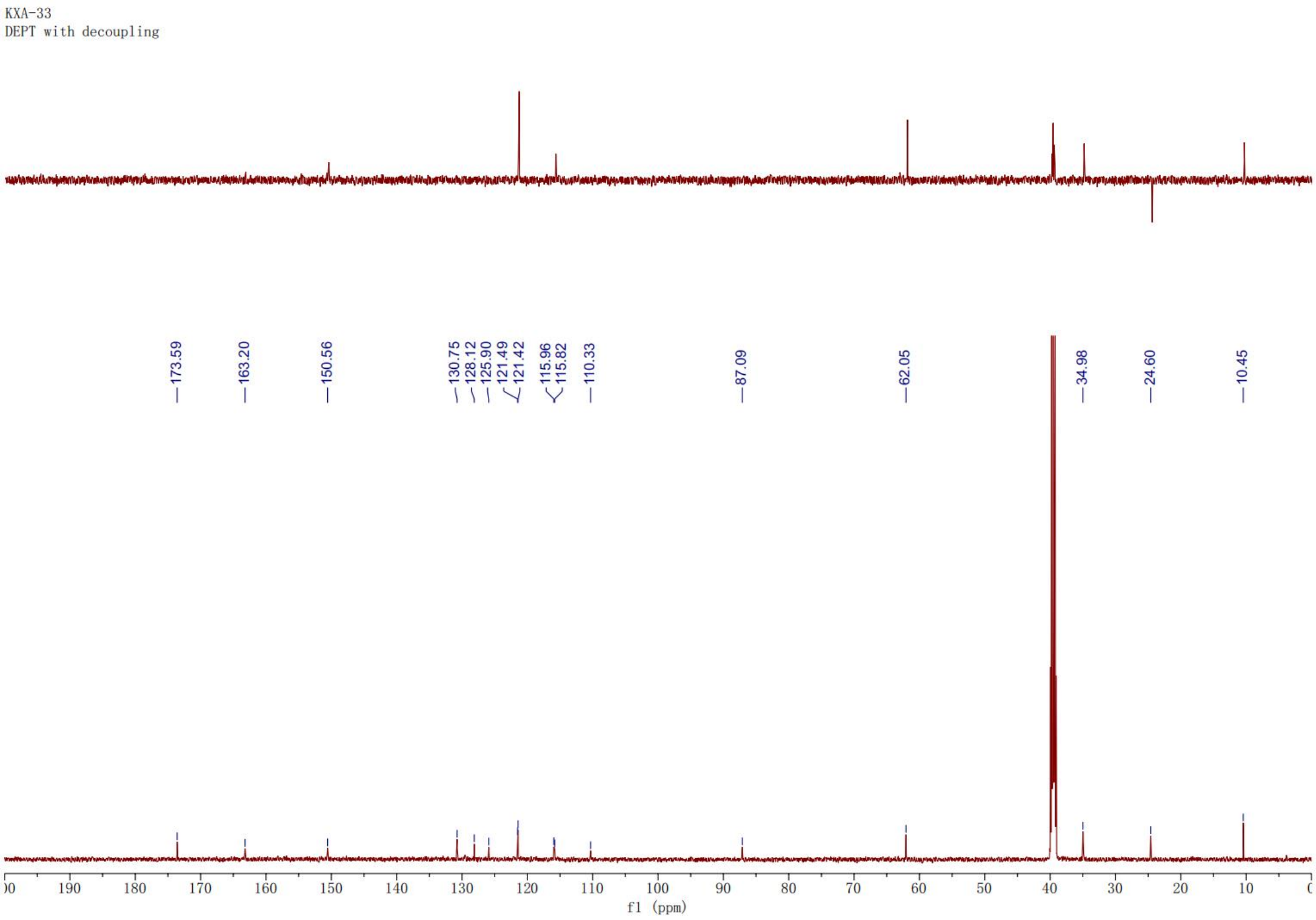

Figure S31. ^{13}C NMR (151 MHz) spectrum of compound **9** in DMSO-*d*6.

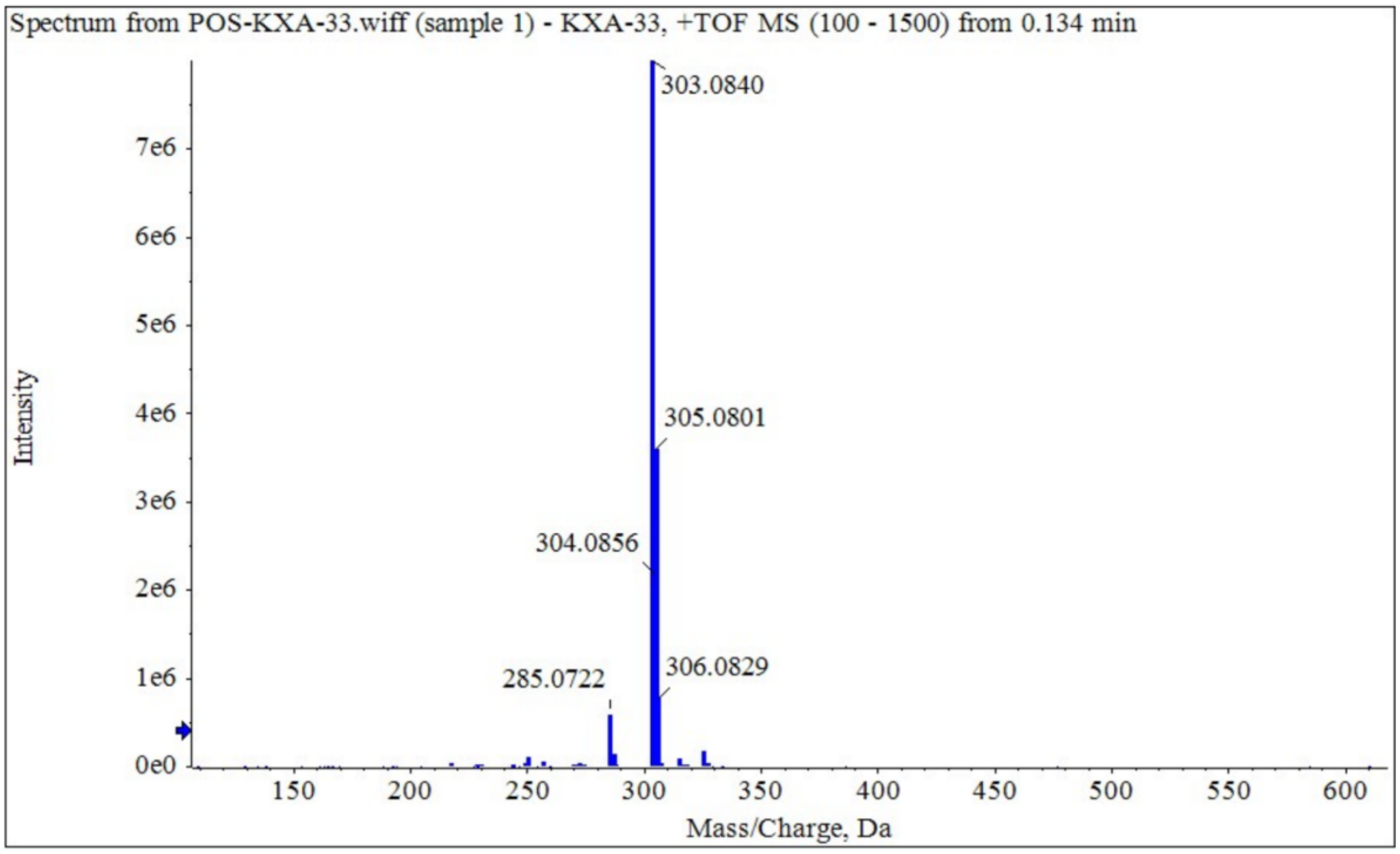

Figure S32. The positive HR-ESI-MS spectrum of compound **9**.

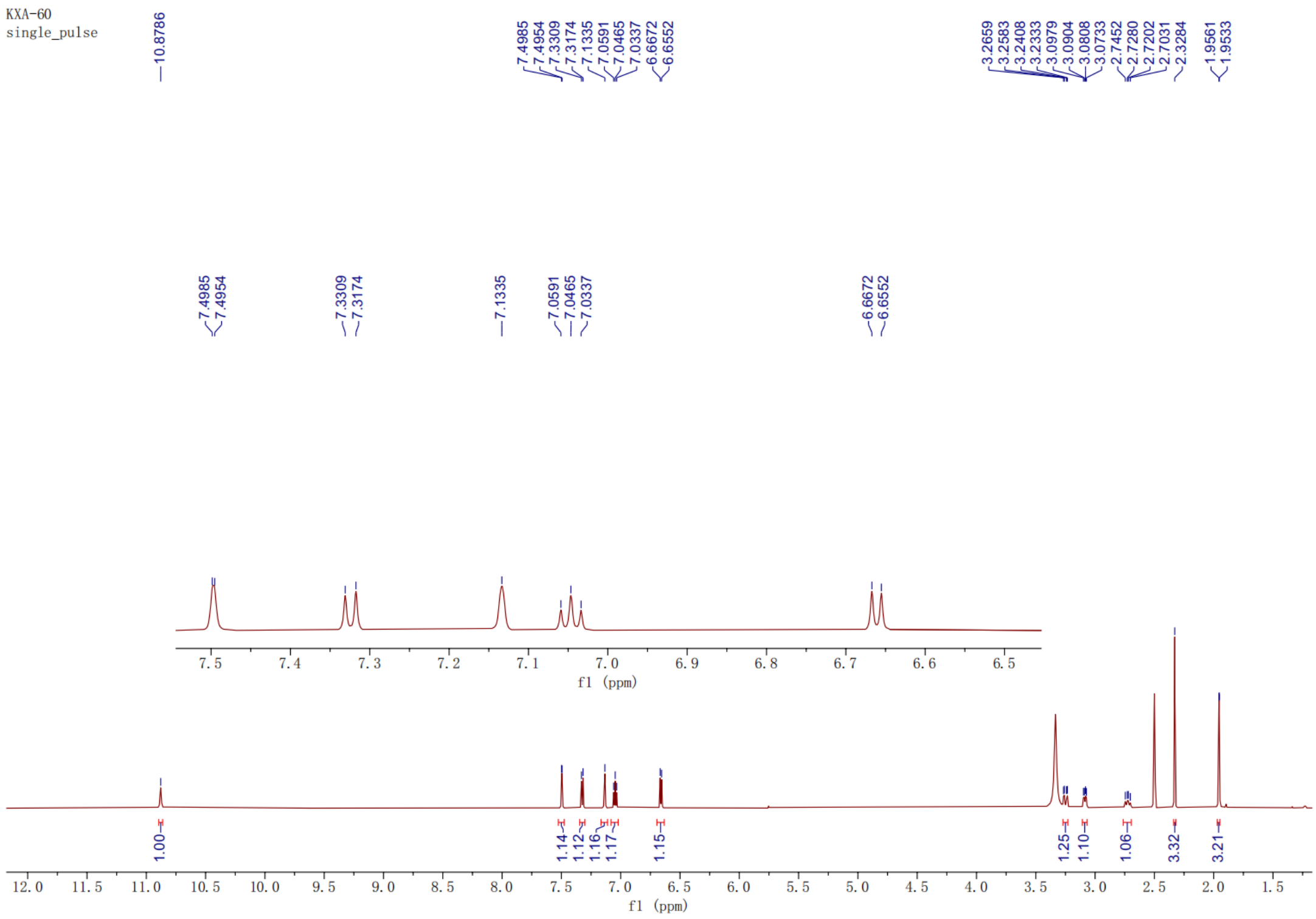

Figure S33. ^{1}H NMR (600 MHz) spectrum of compound **10** in DMSO-d_6.

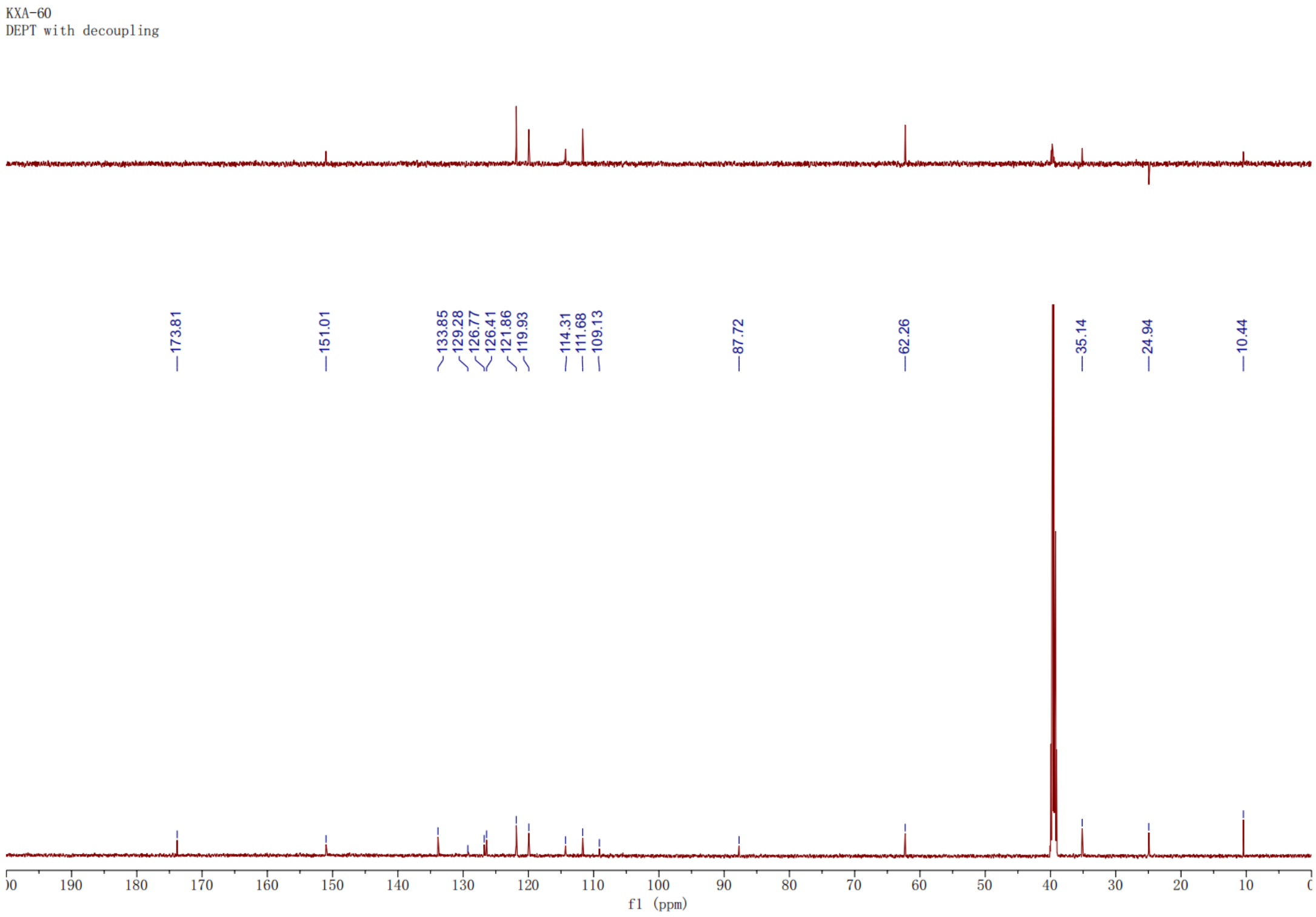

Figure S34. ^{13}C NMR (151 MHz) spectrum of compound **10** in DMSO-d_6.

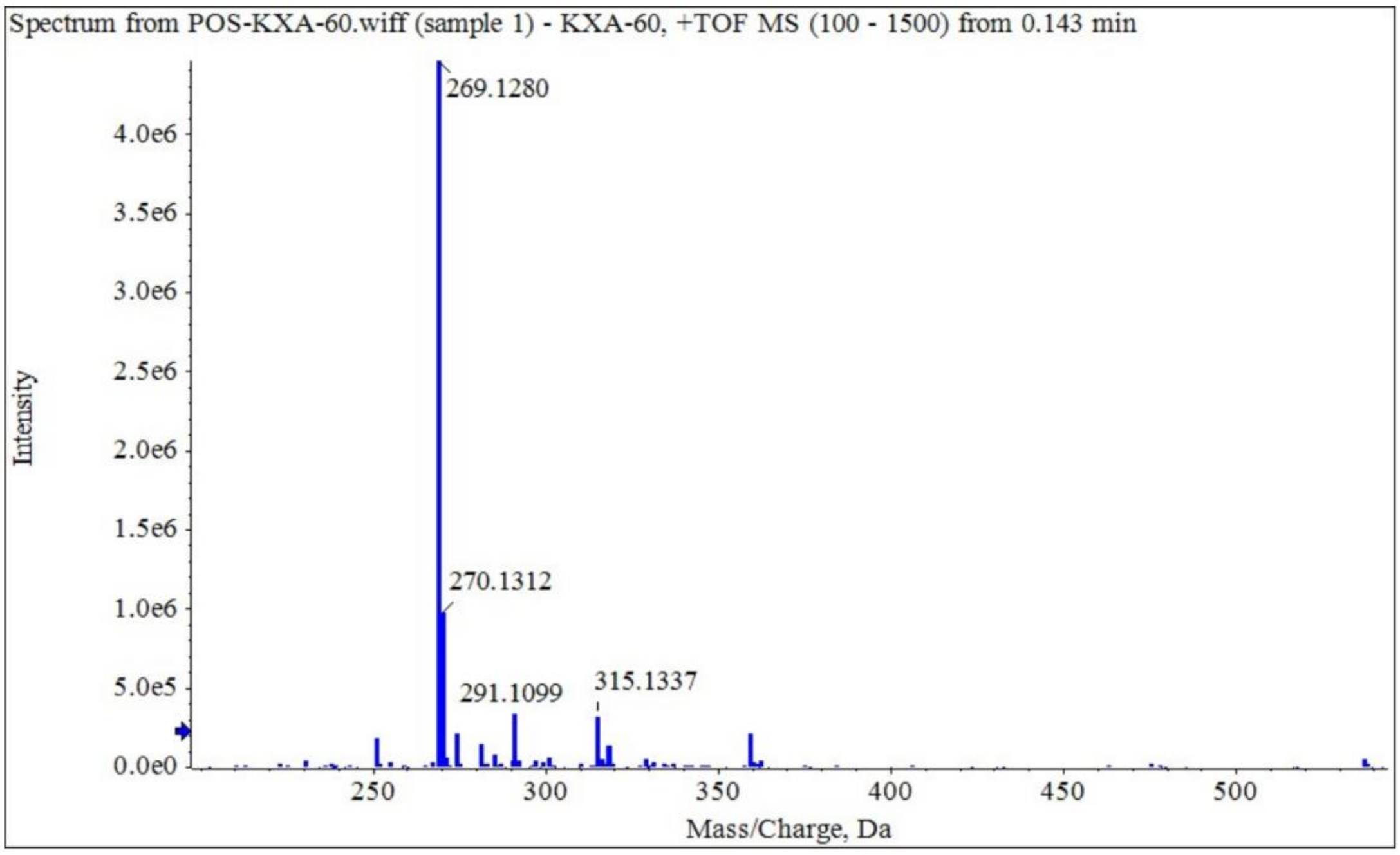

Figure S35. The positive HR-ESI-MS spectrum of compound **10**.

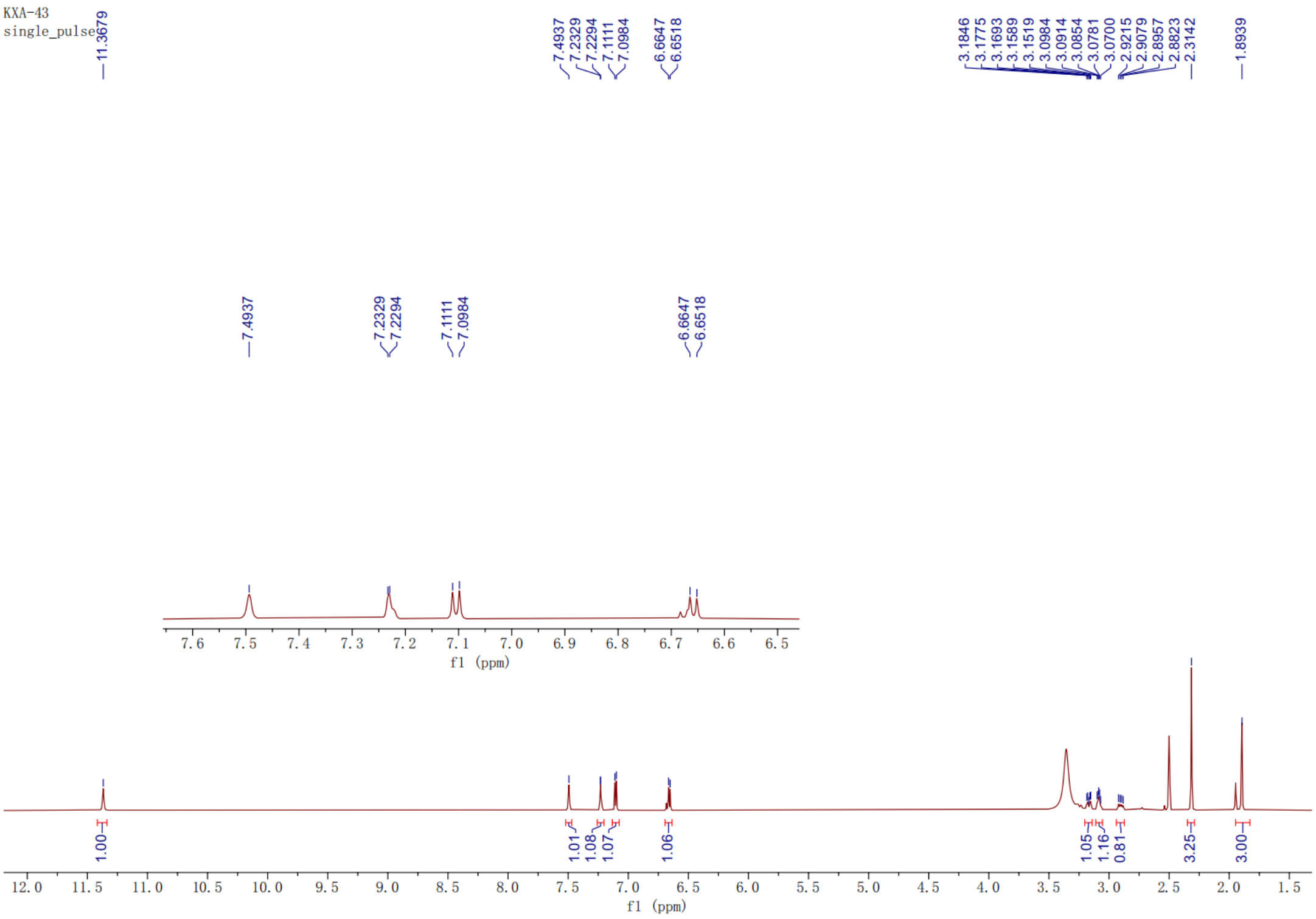

Figure S36. ^{1}H NMR (600 MHz) spectrum of compound **11** in DMSO-d_6.

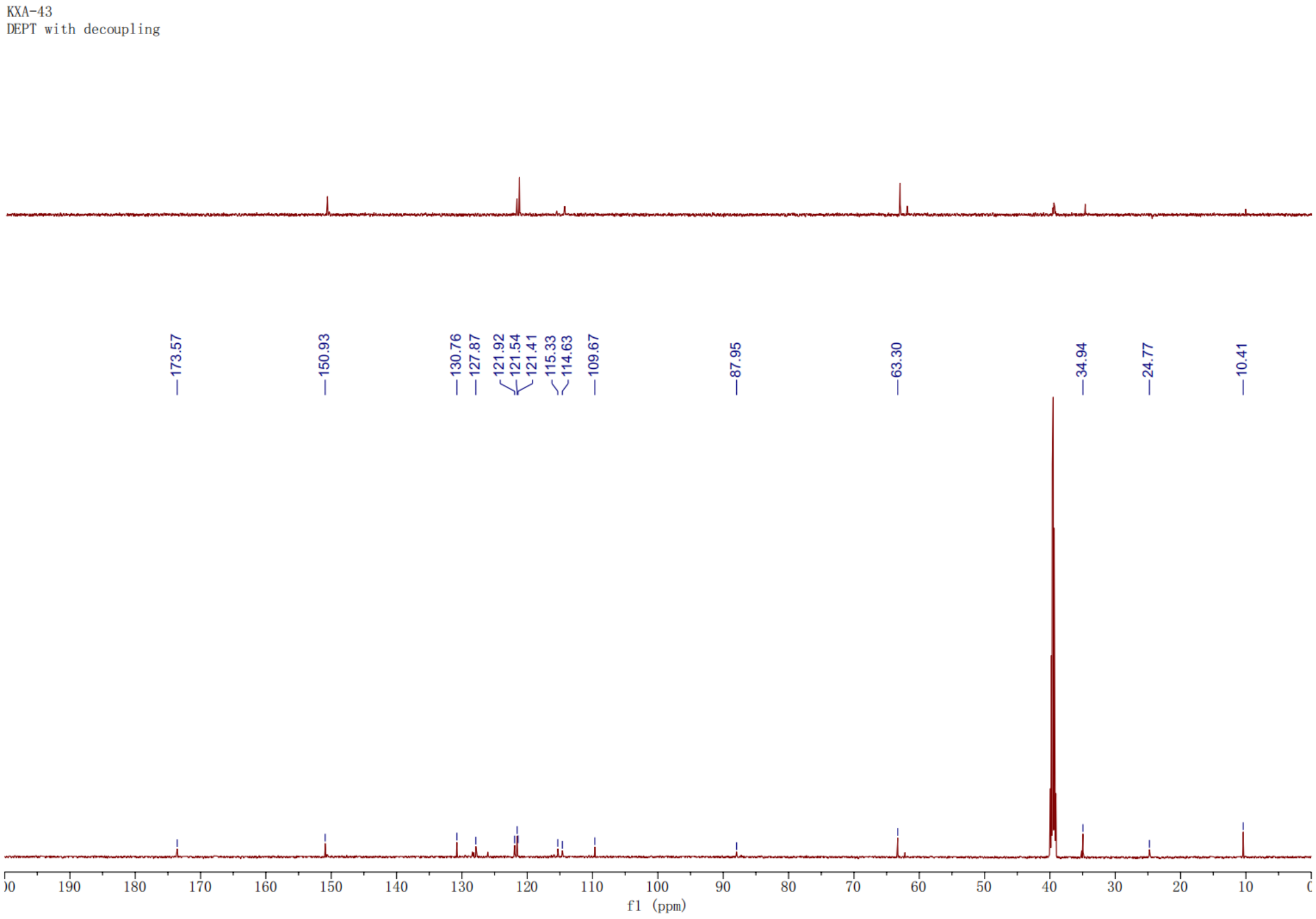

Figure S37. ^{13}C NMR (151 MHz) spectrum of compound **11** in DMSO-d_6.

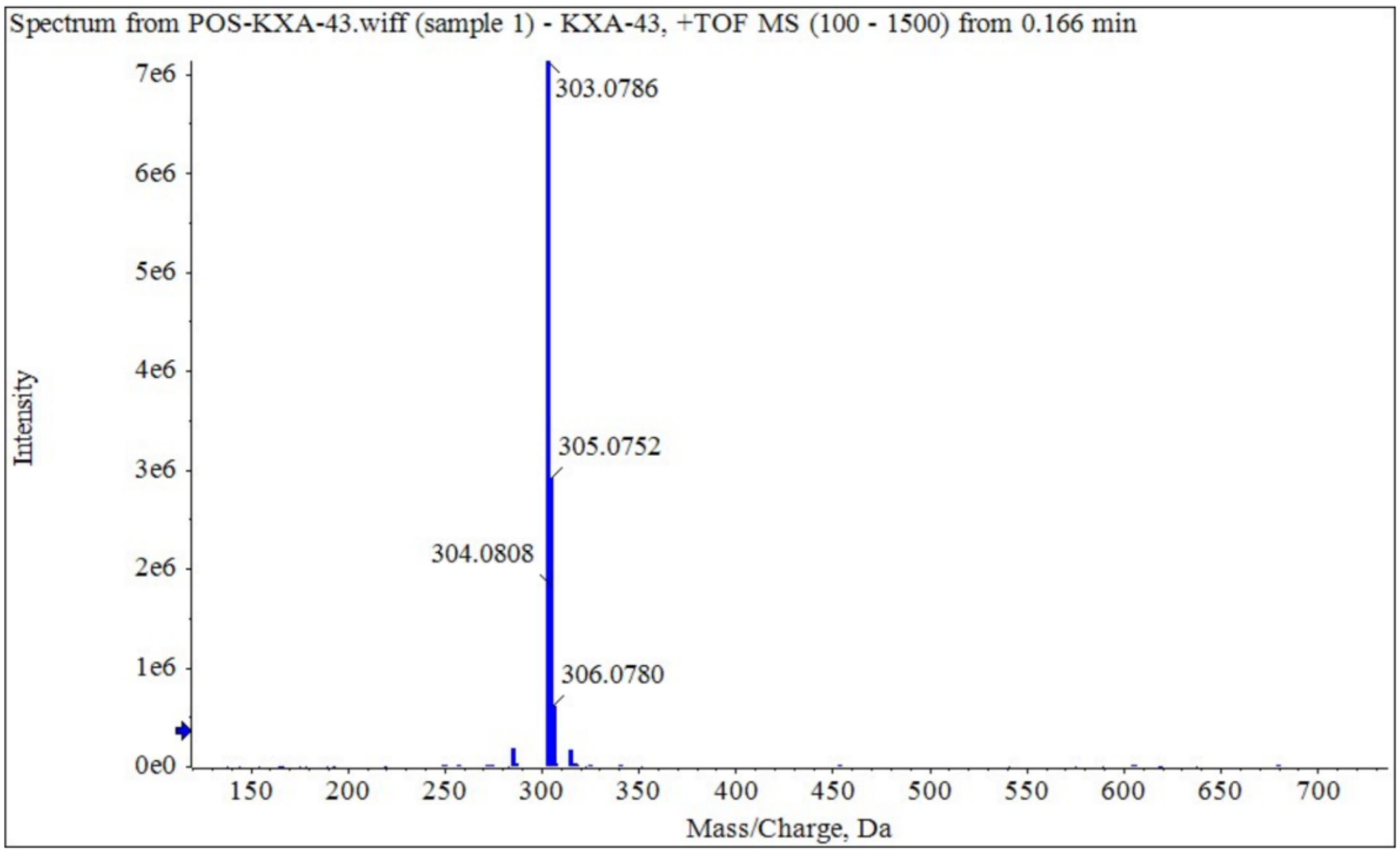

Figure S38. The positive HR-ESI-MS spectrum of compound **11**.

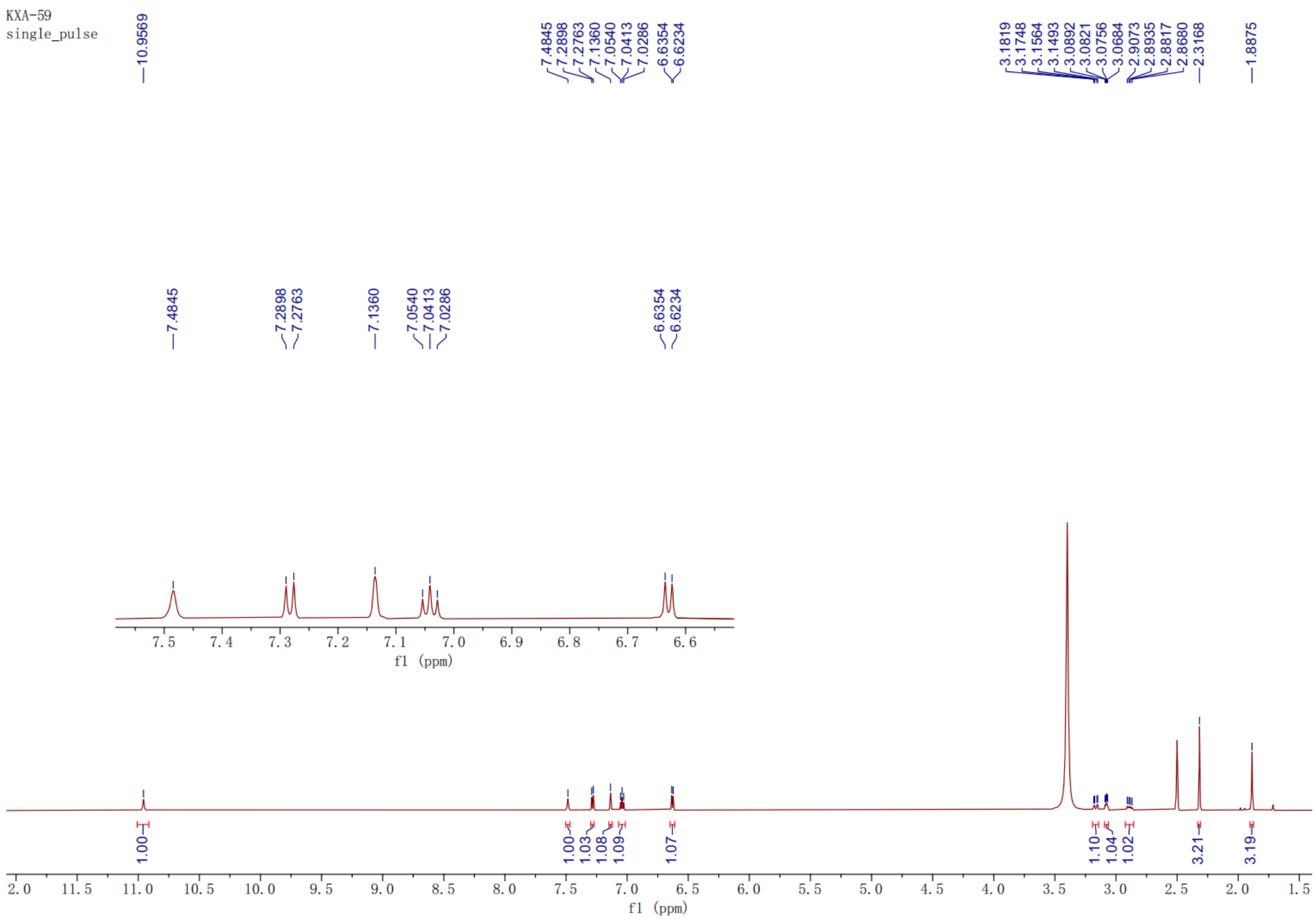

Figure S39. ^{1}H NMR (600 MHz) spectrum of compound **12** in DMSO-d_6.

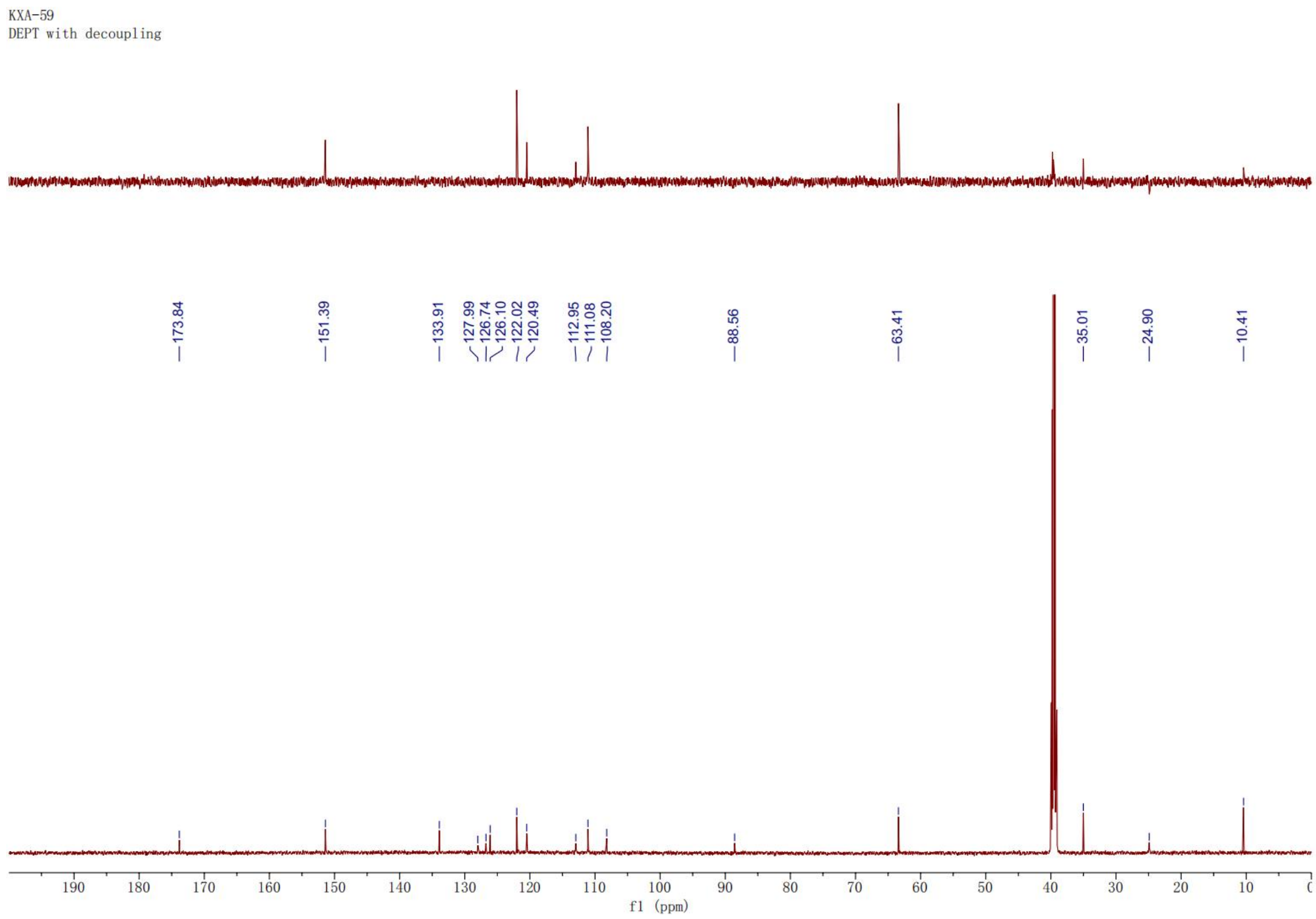

Figure S40. ^{13}C NMR (151 MHz) spectrum of compound **12** in DMSO-d_6.

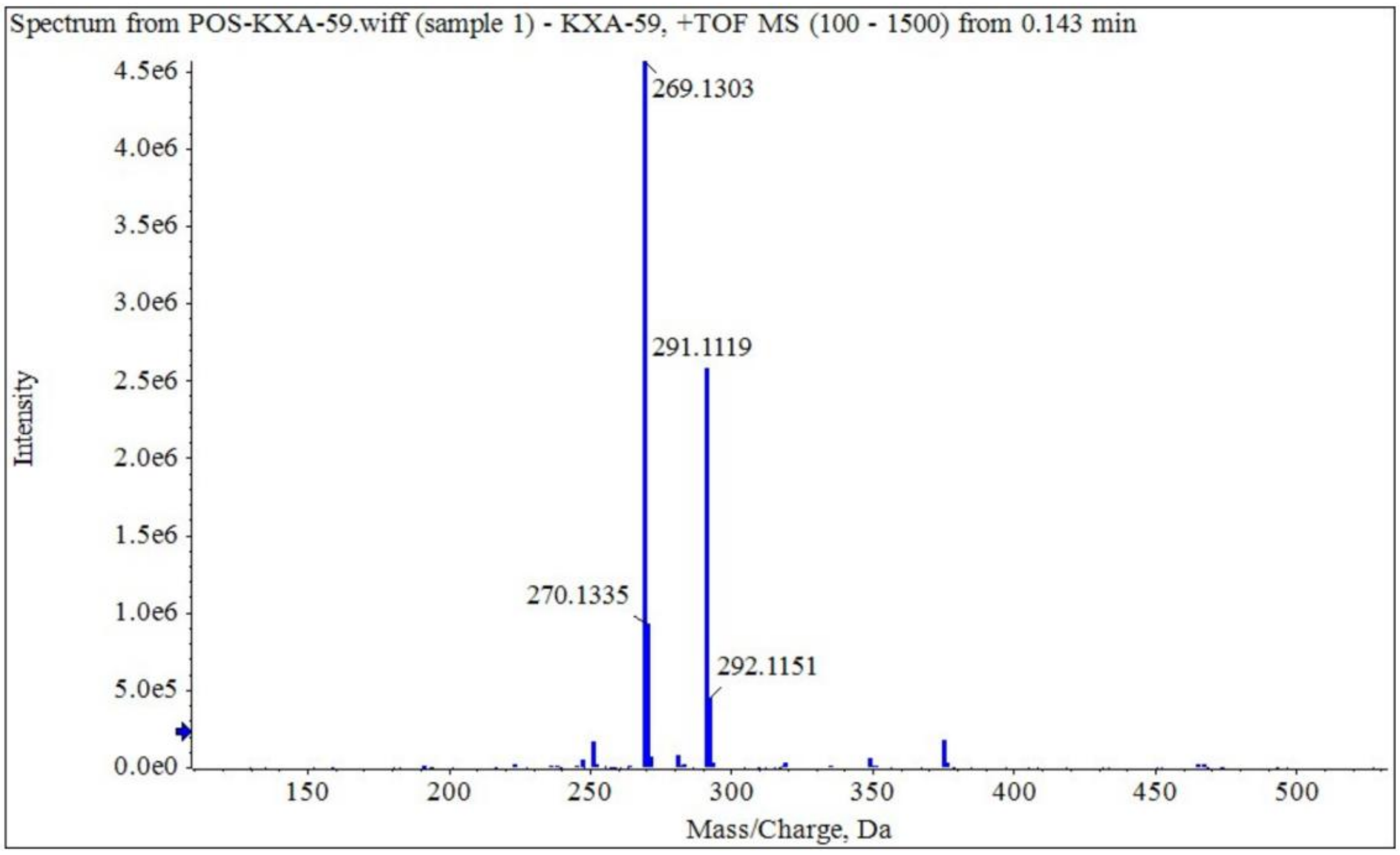

Figure S41. The positive HR-ESI-MS spectrum of compound **12**.

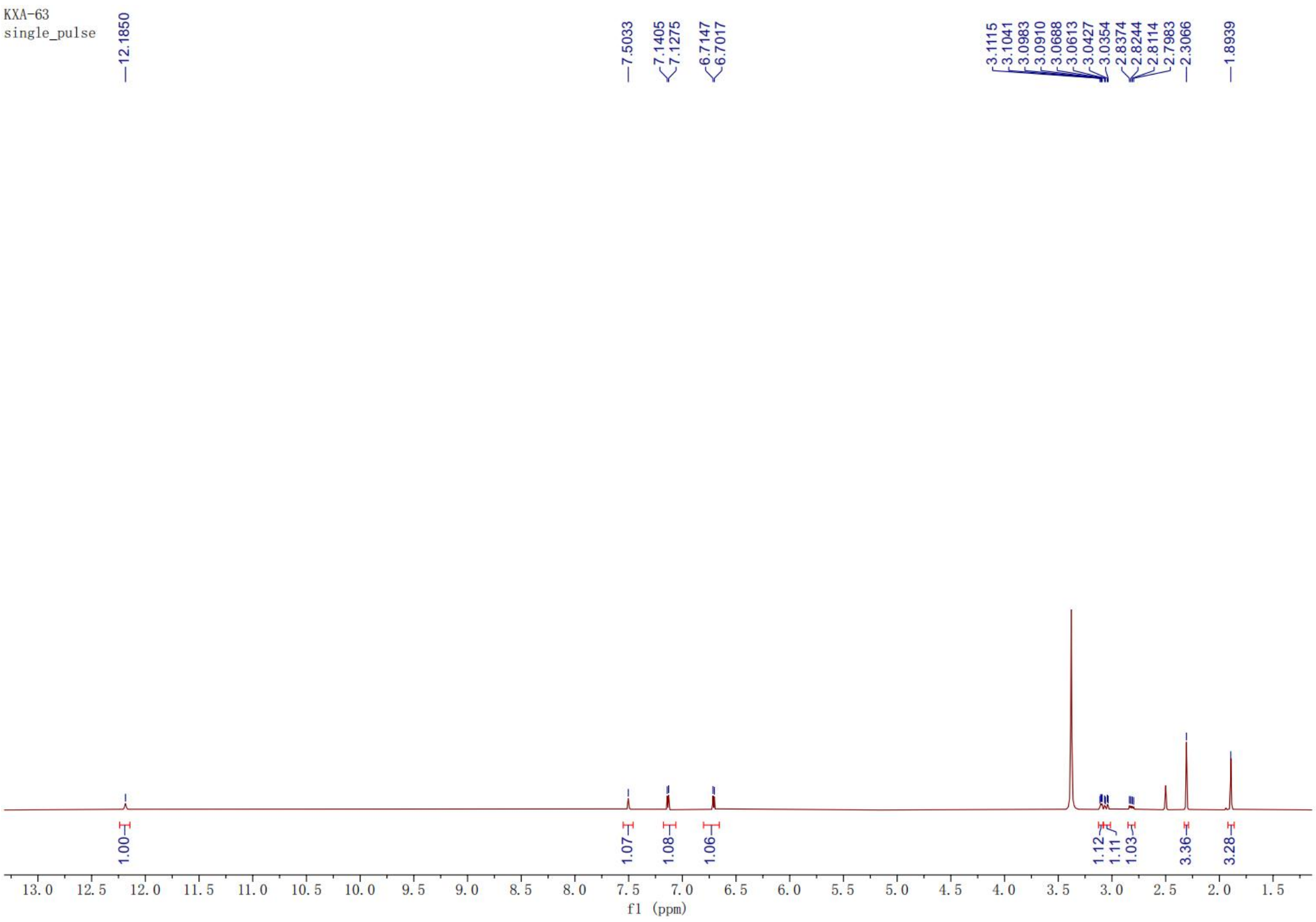

Figure S42. ^{1}H NMR (600 MHz) spectrum of compound **13** in DMSO-d_6.

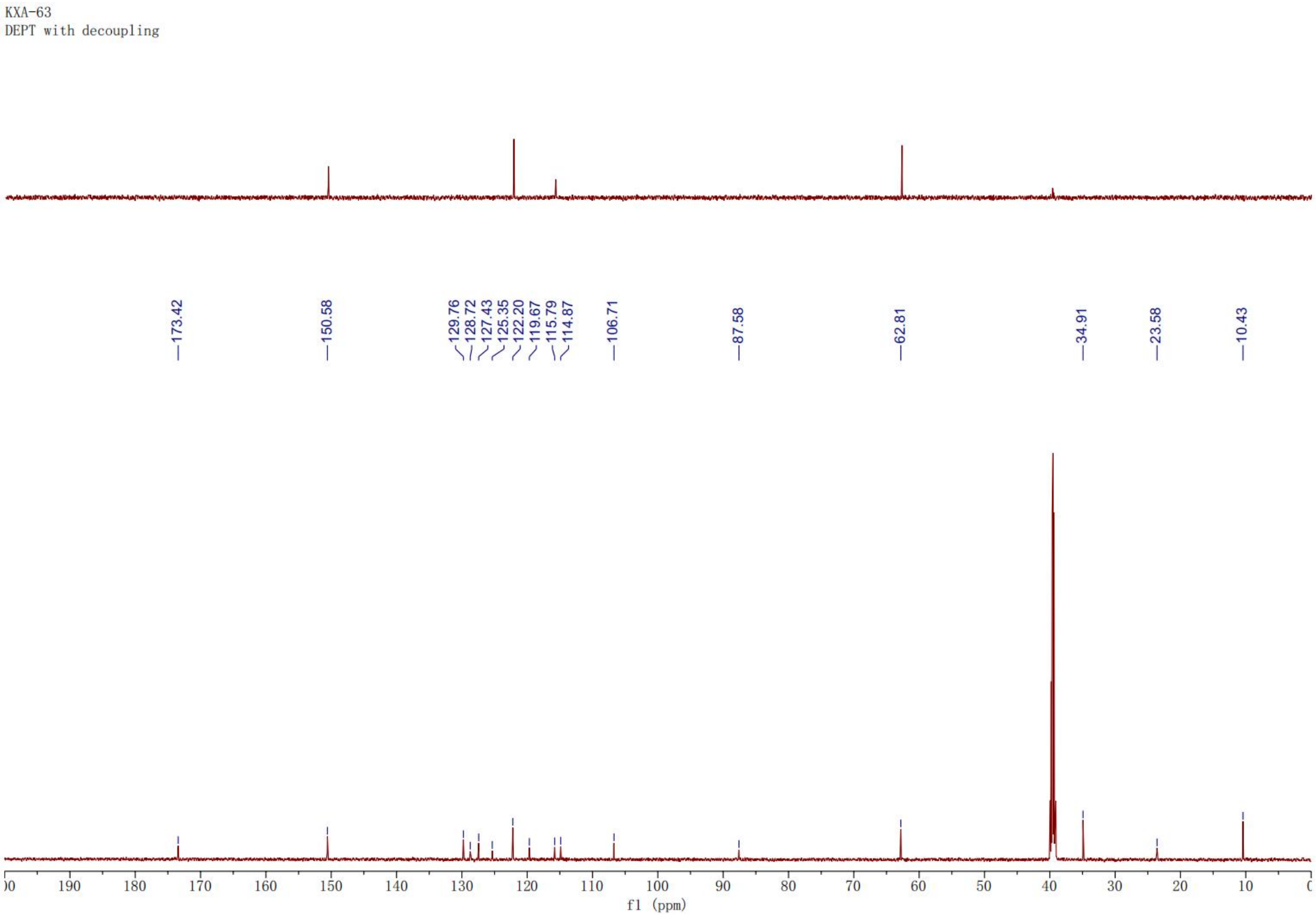

Figure S43. ^{13}C NMR (151 MHz) spectrum of compound **13** in DMSO-d_6.

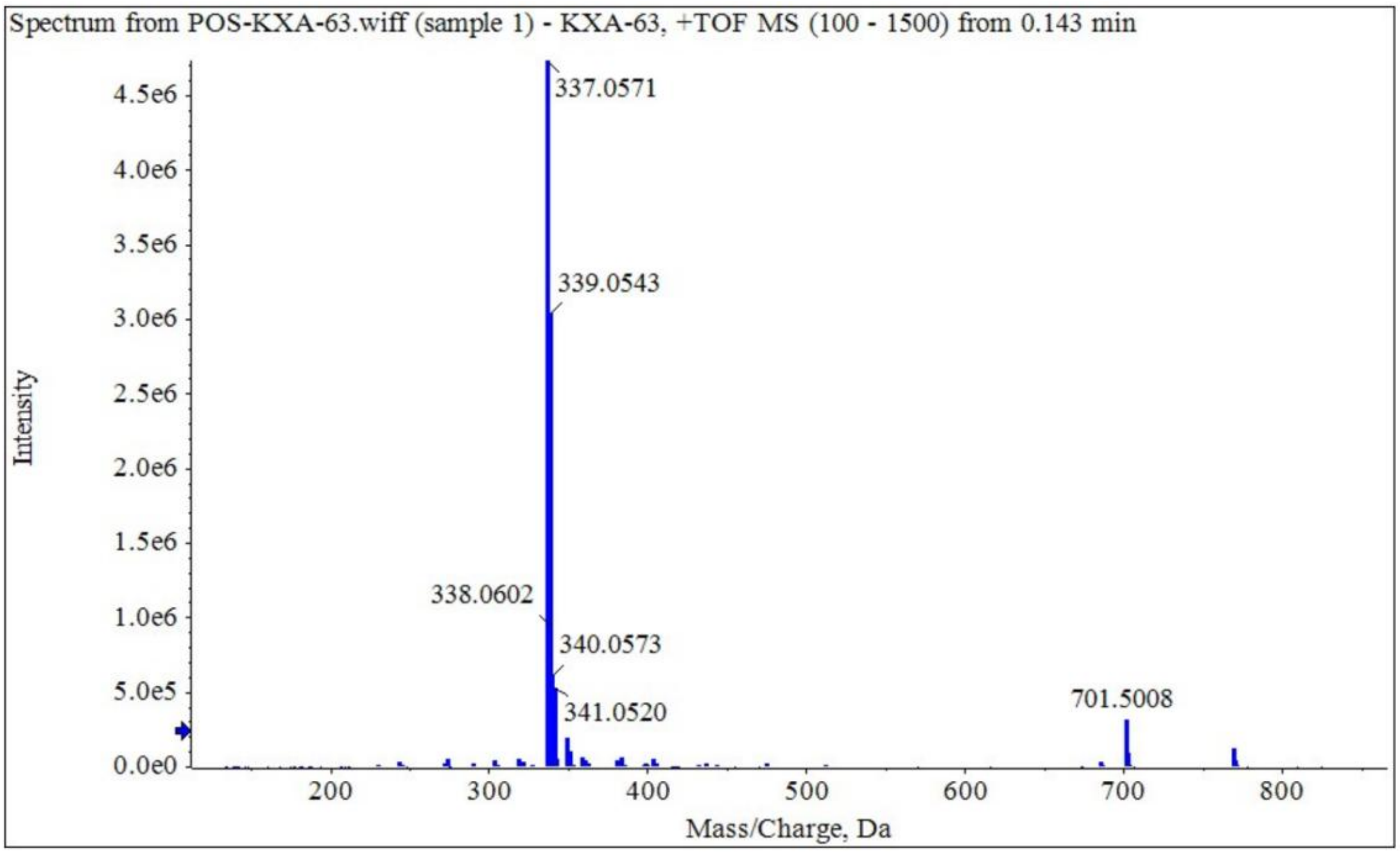

Figure S44. The positive HR-ESI-MS spectrum of compound **13**.

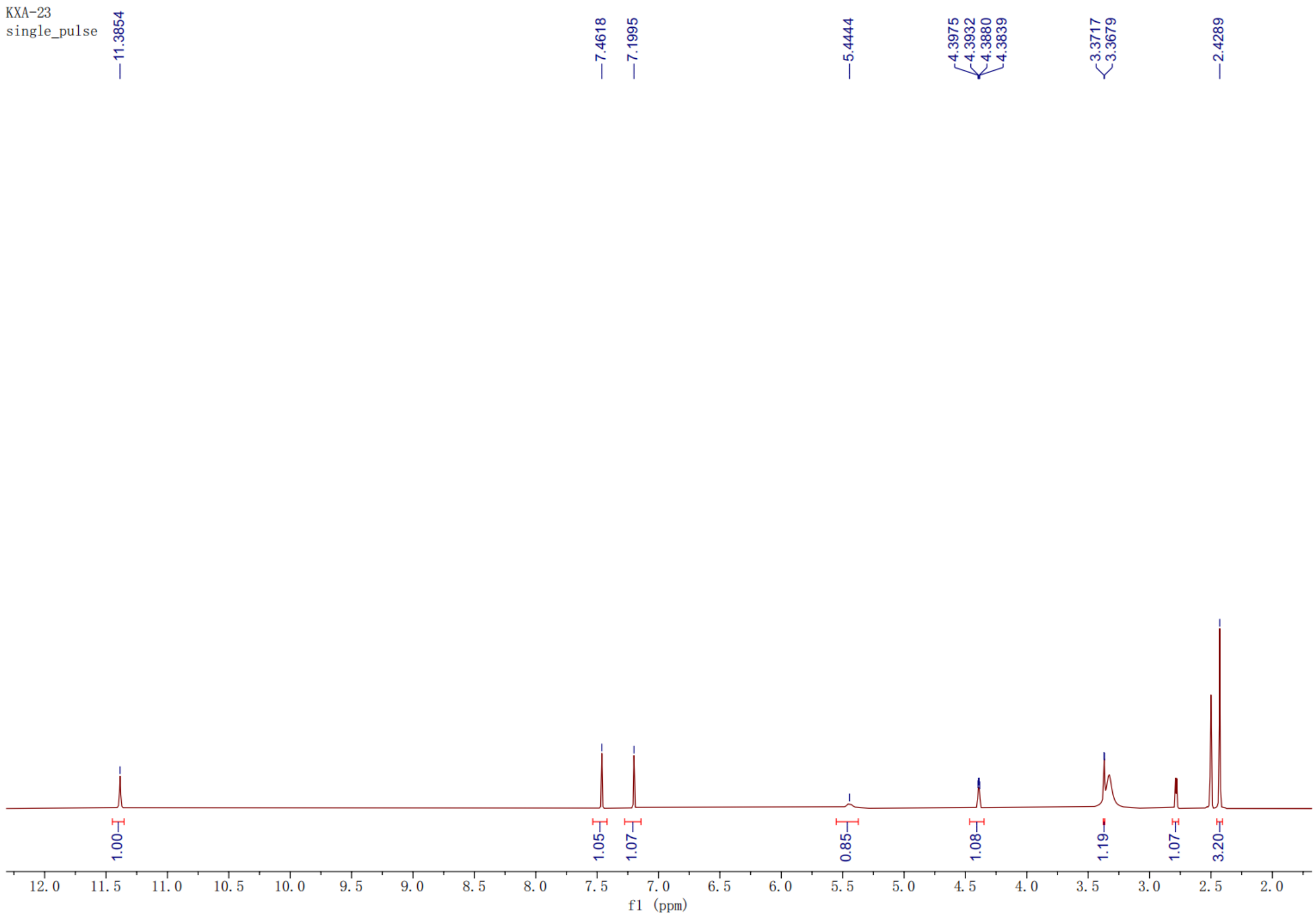

Figure S45. ^{1}H NMR (600 MHz) spectrum of compound **14** in DMSO-d_6.

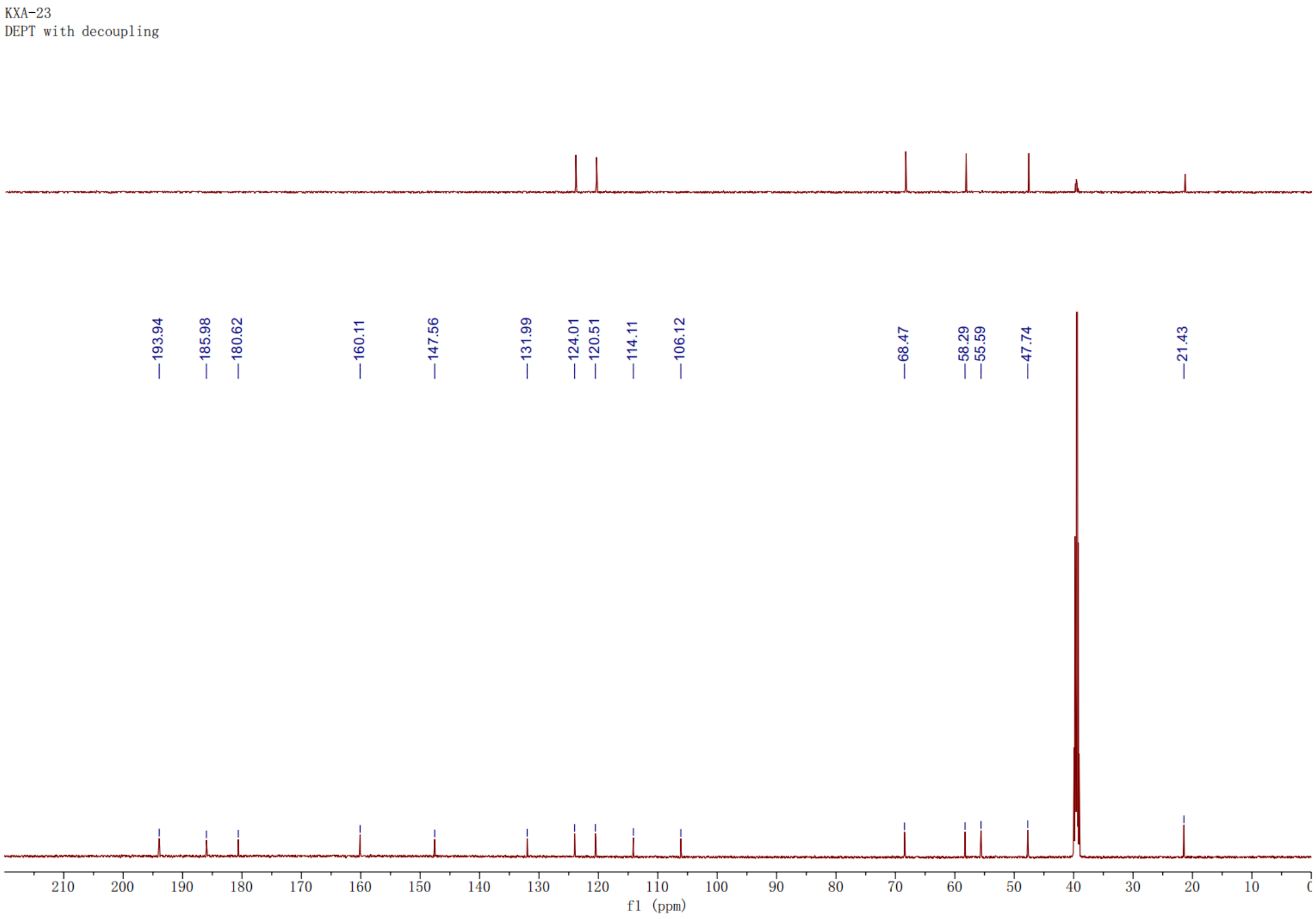

Figure S46. ^{13}C NMR (151 MHz) spectrum of compound **14** in DMSO-d_6.

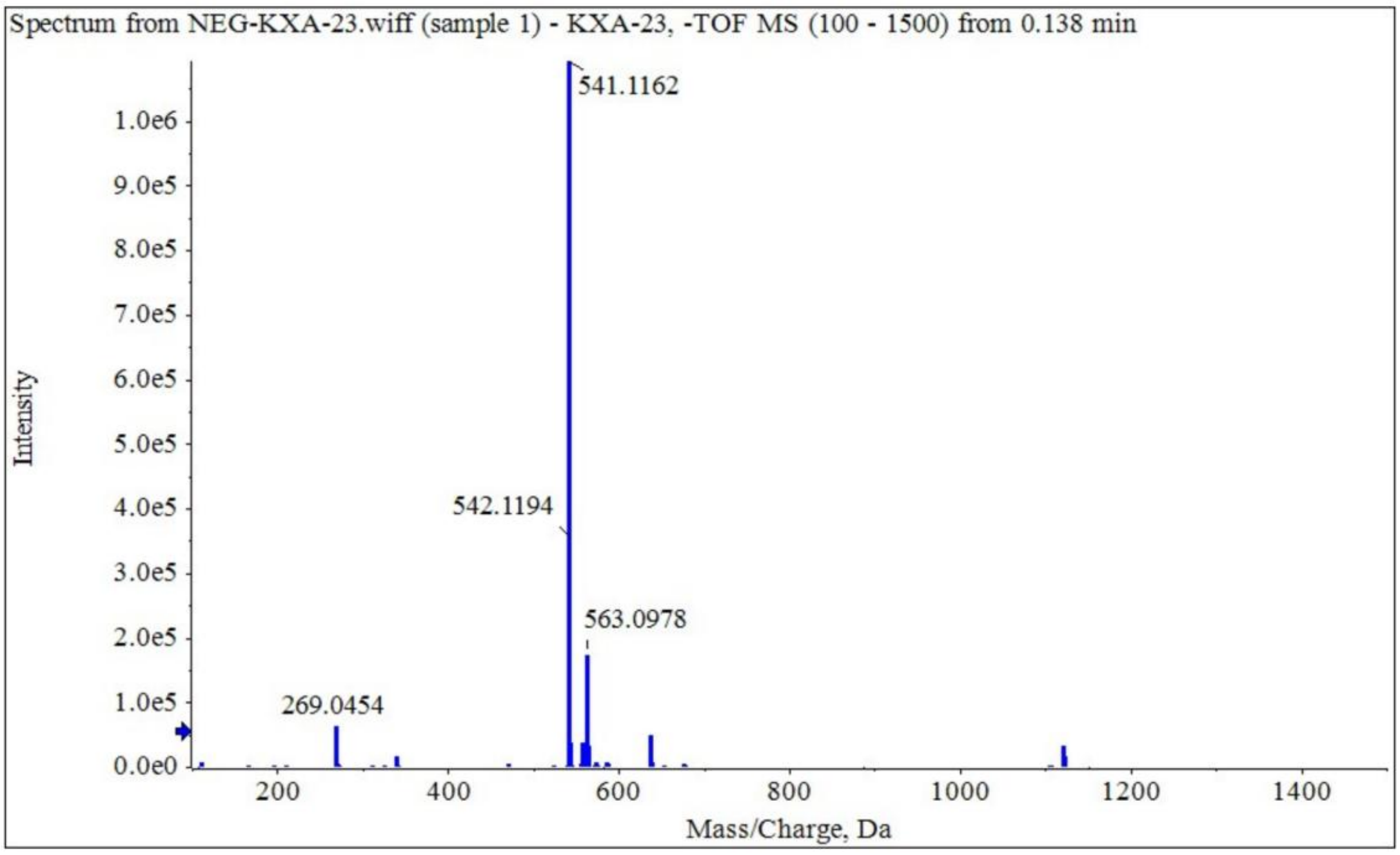

Figure S47. The negative HR-ESI-MS spectrum of compound **14**.

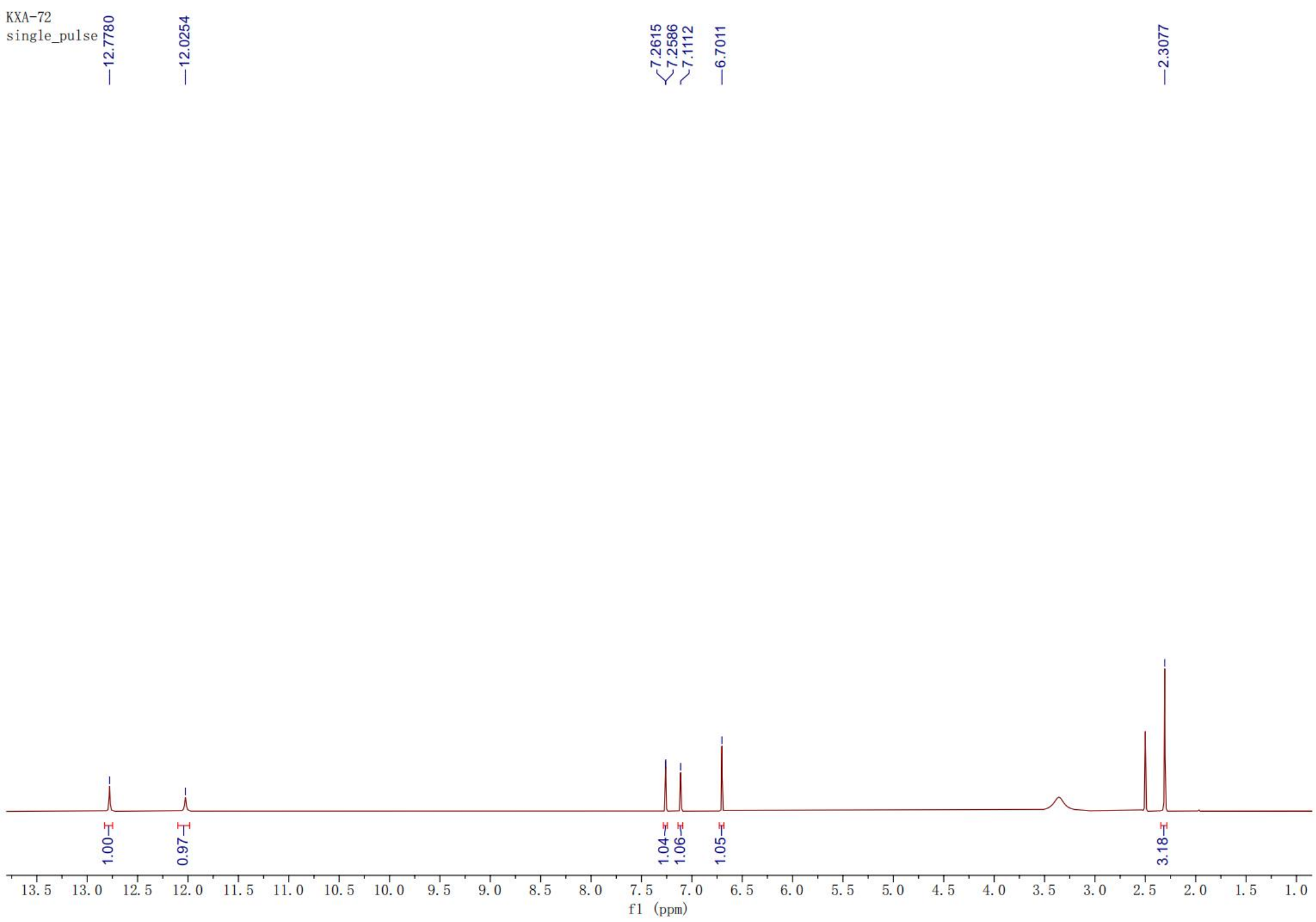

Figure S48. ^{1}H NMR (600 MHz) spectrum of compound **15** in DMSO-d_6.

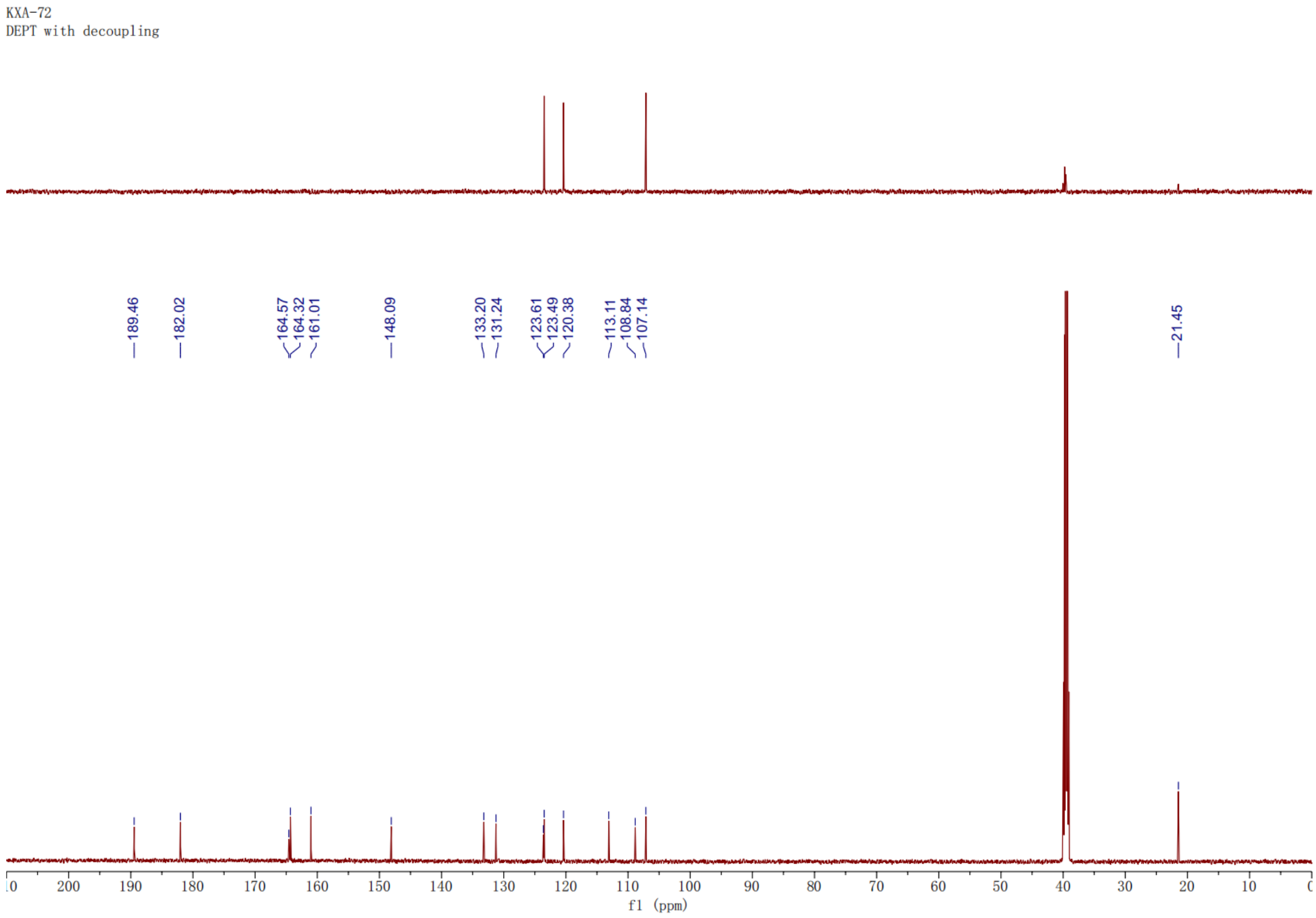

Figure S49. ^{13}C NMR (151 MHz) spectrum of compound **15** in DMSO-d_6.

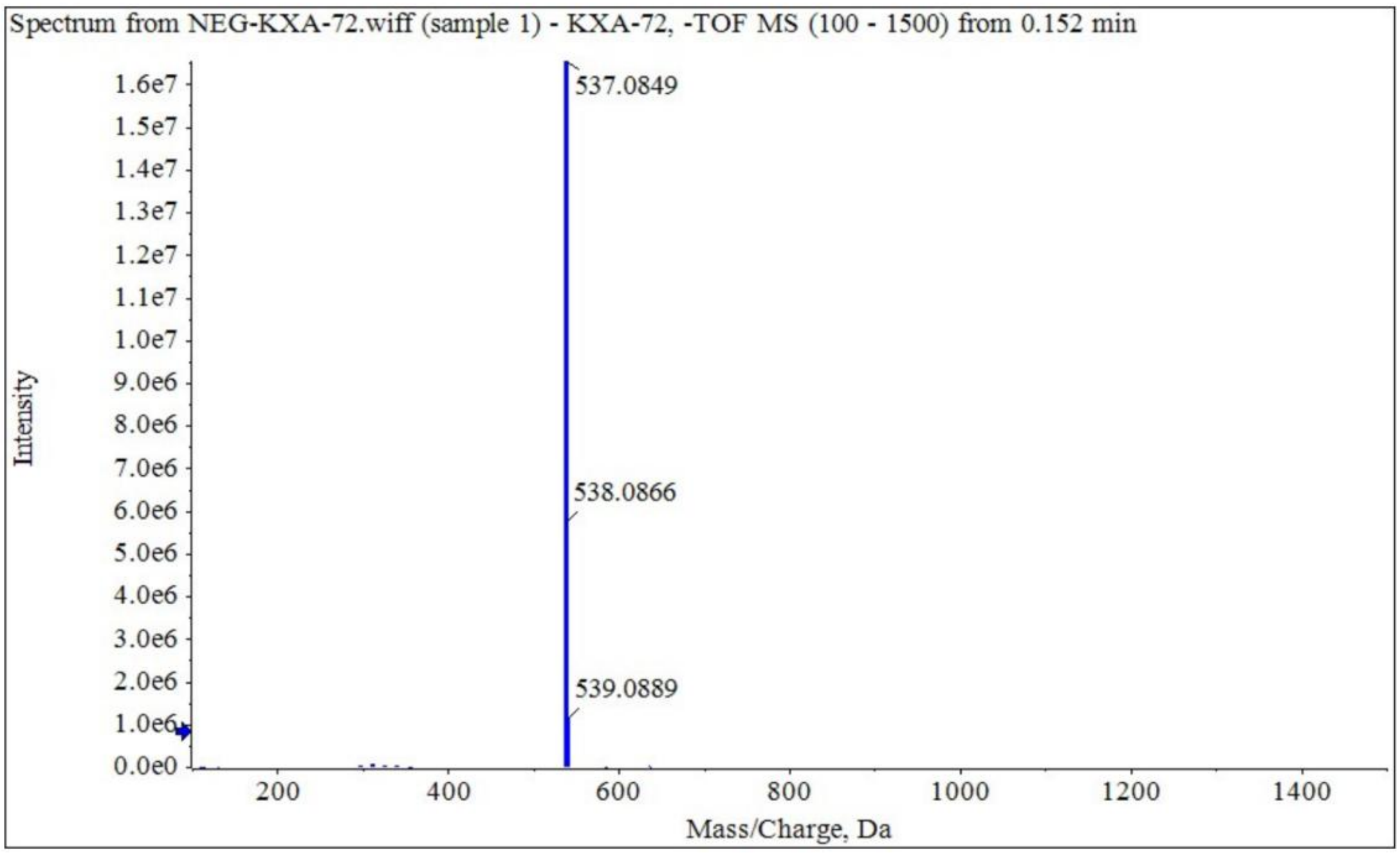

Figure S50. The negative HR-ESI-MS spectrum of compound **15**.

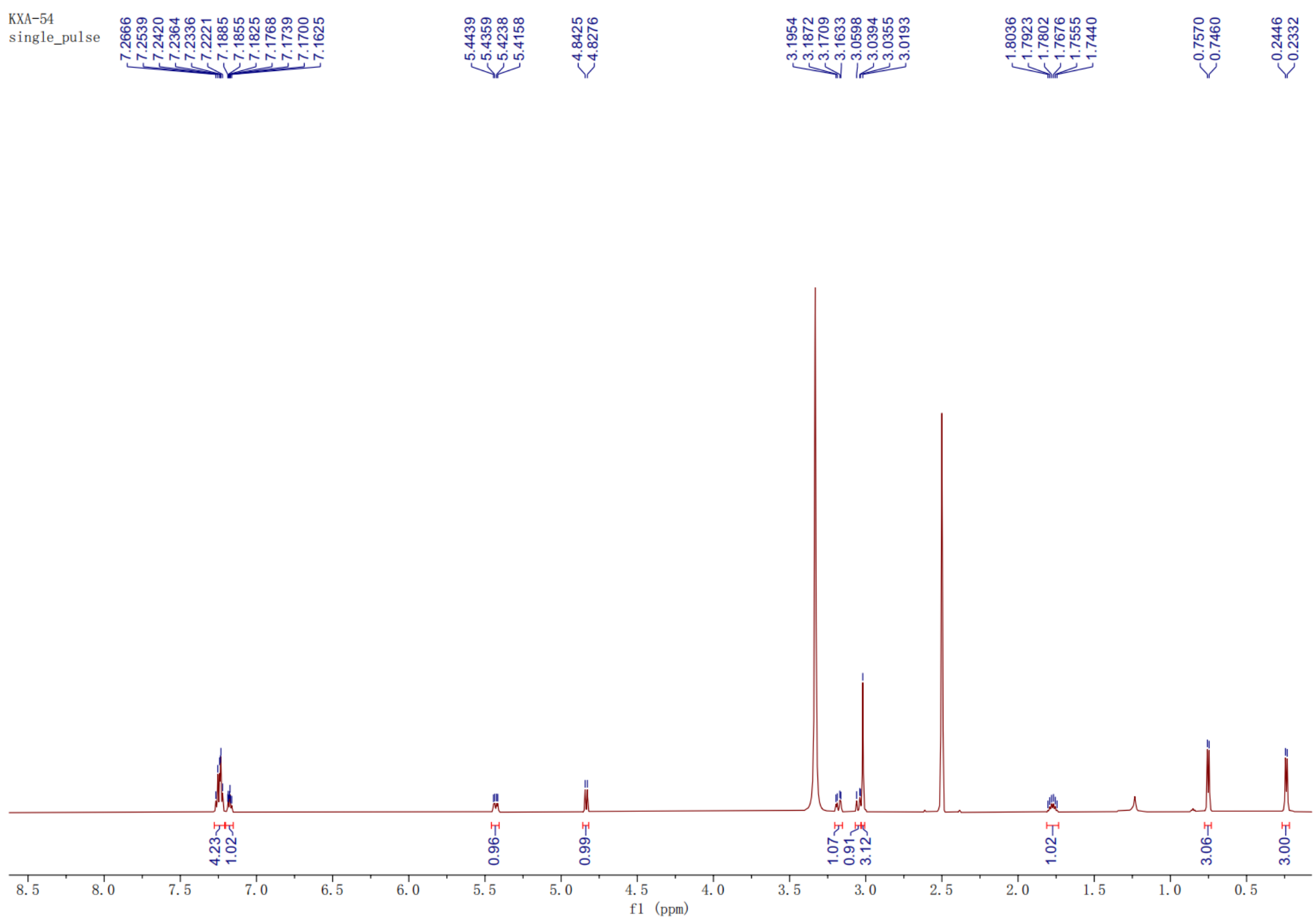

Figure S51. ^{1}H NMR (600 MHz) spectrum of compound **16** in DMSO-d_6.

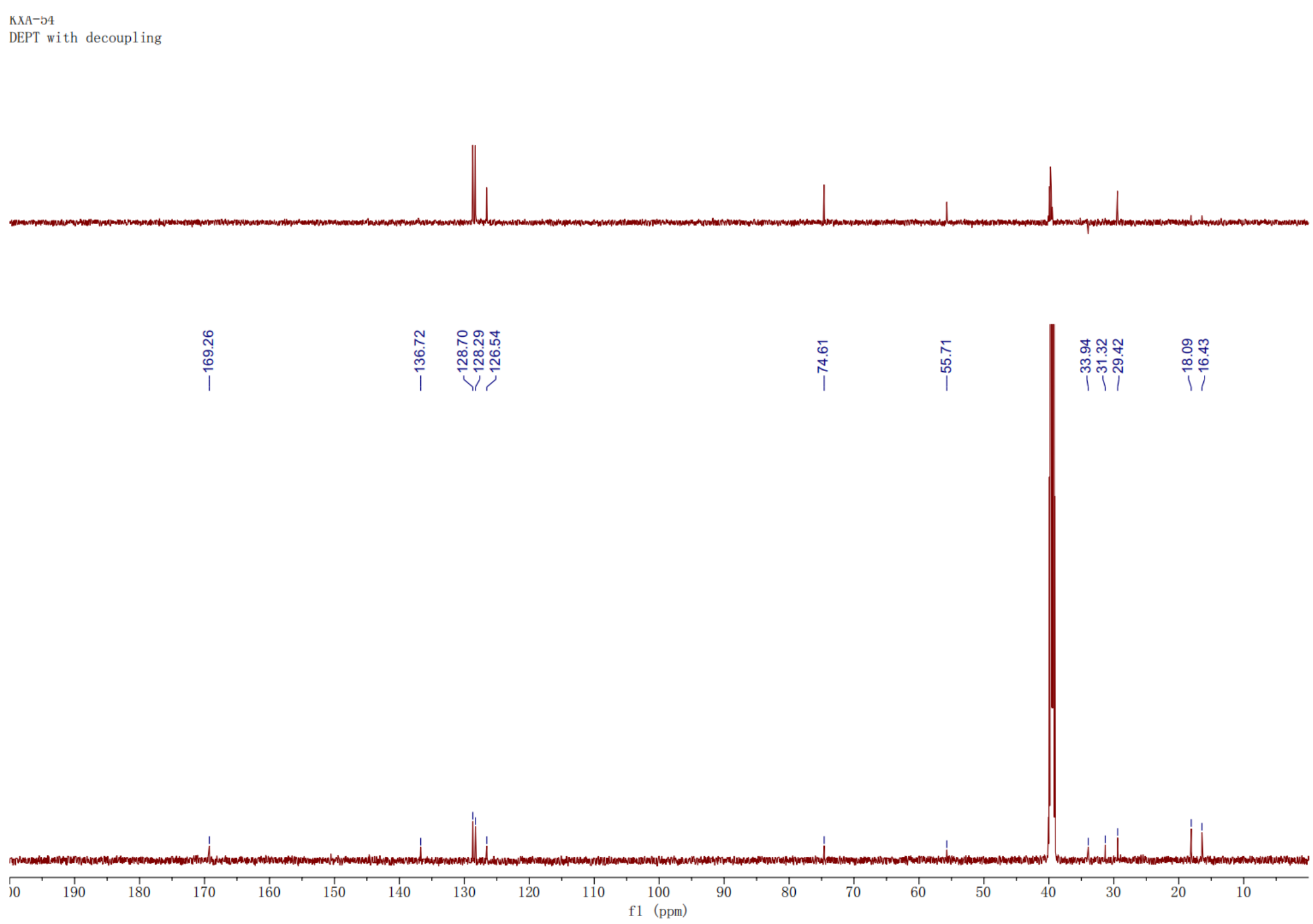

Figure S52. ^{13}C NMR (151 MHz) spectrum of compound **16** in DMSO-d_6.

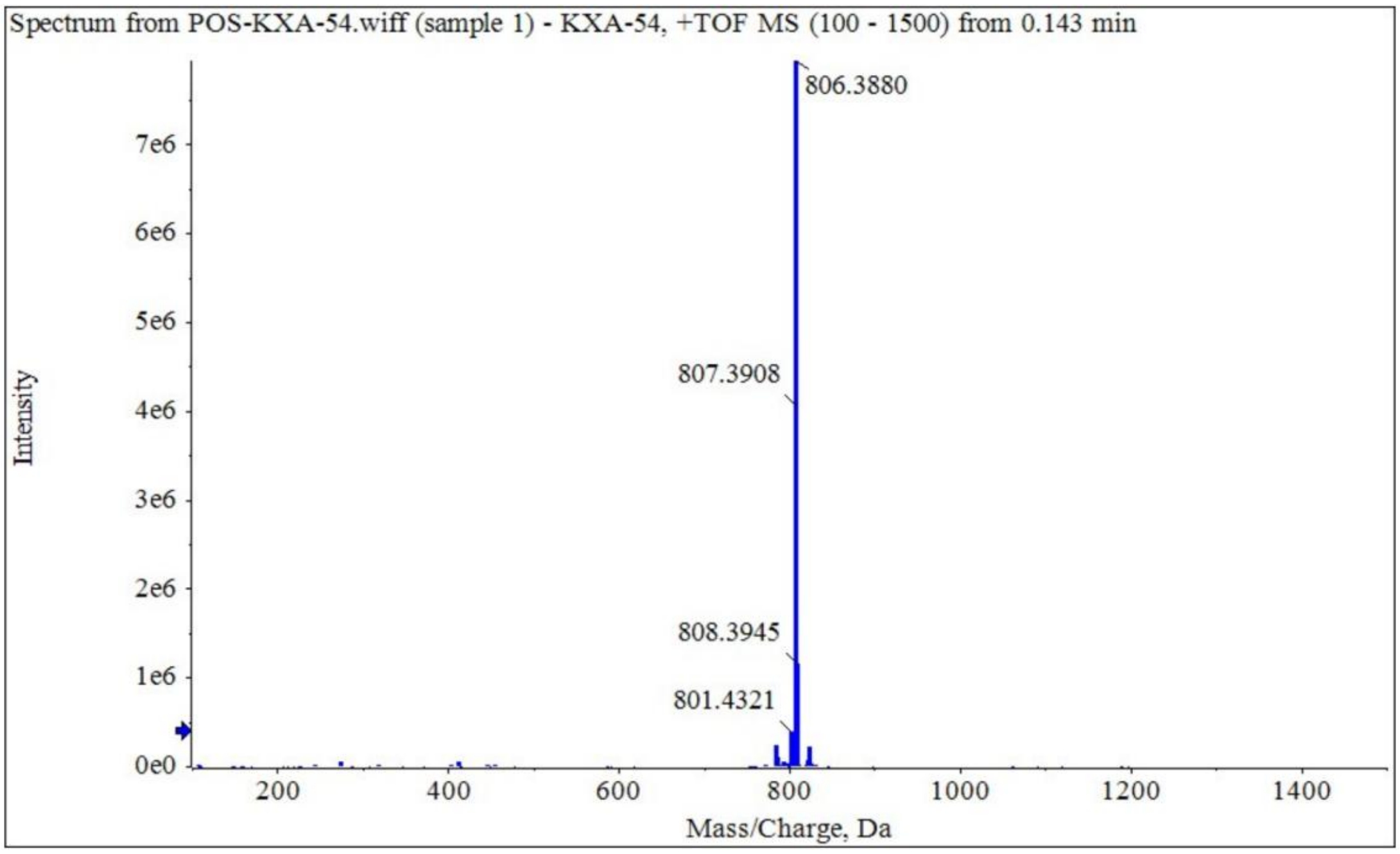

Figure S53. The positive HR-ESI-MS spectrum of compound **16**.

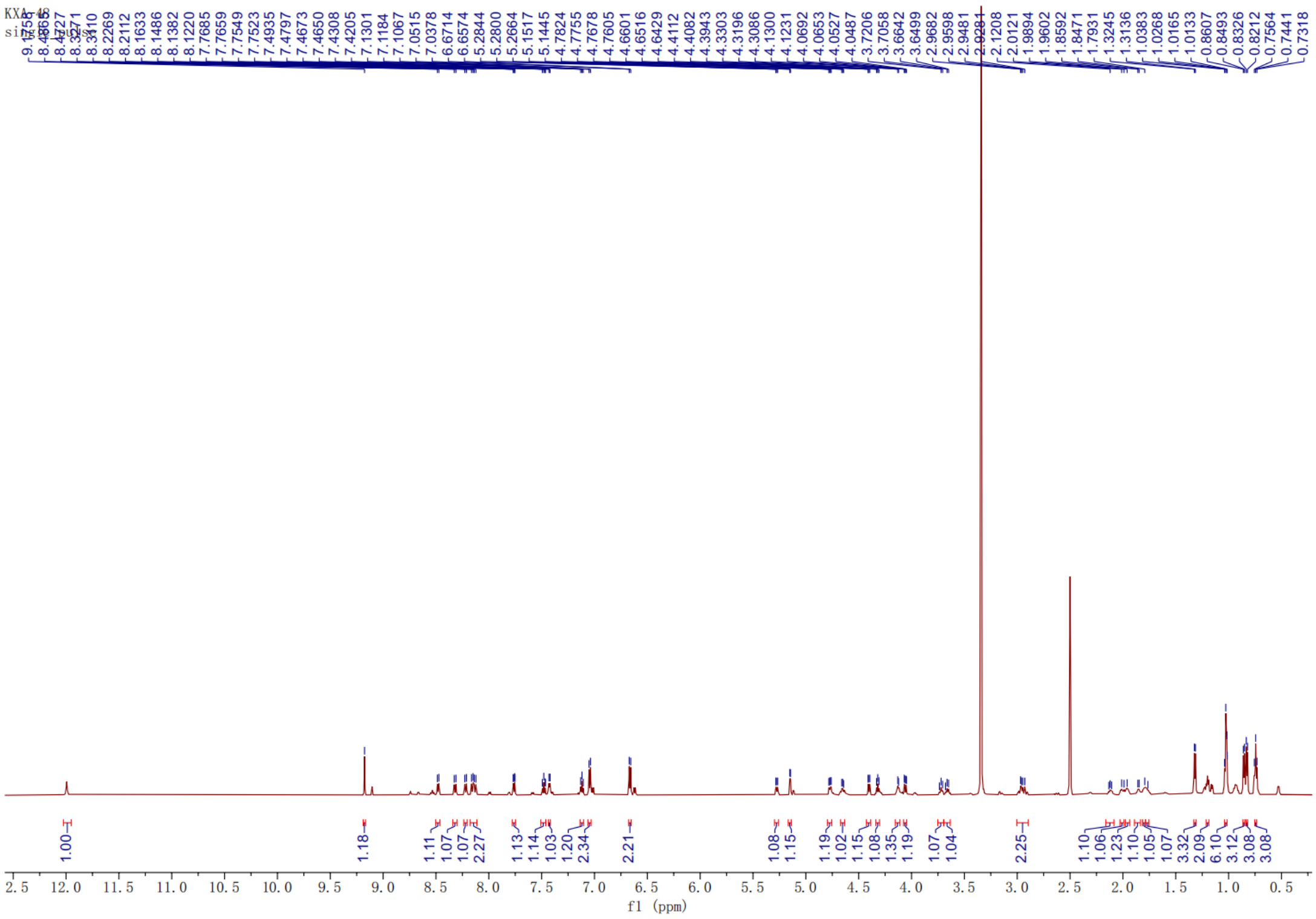

Figure S54. ^{1}H NMR (600 MHz) spectrum of compound **17** in DMSO-d_6.

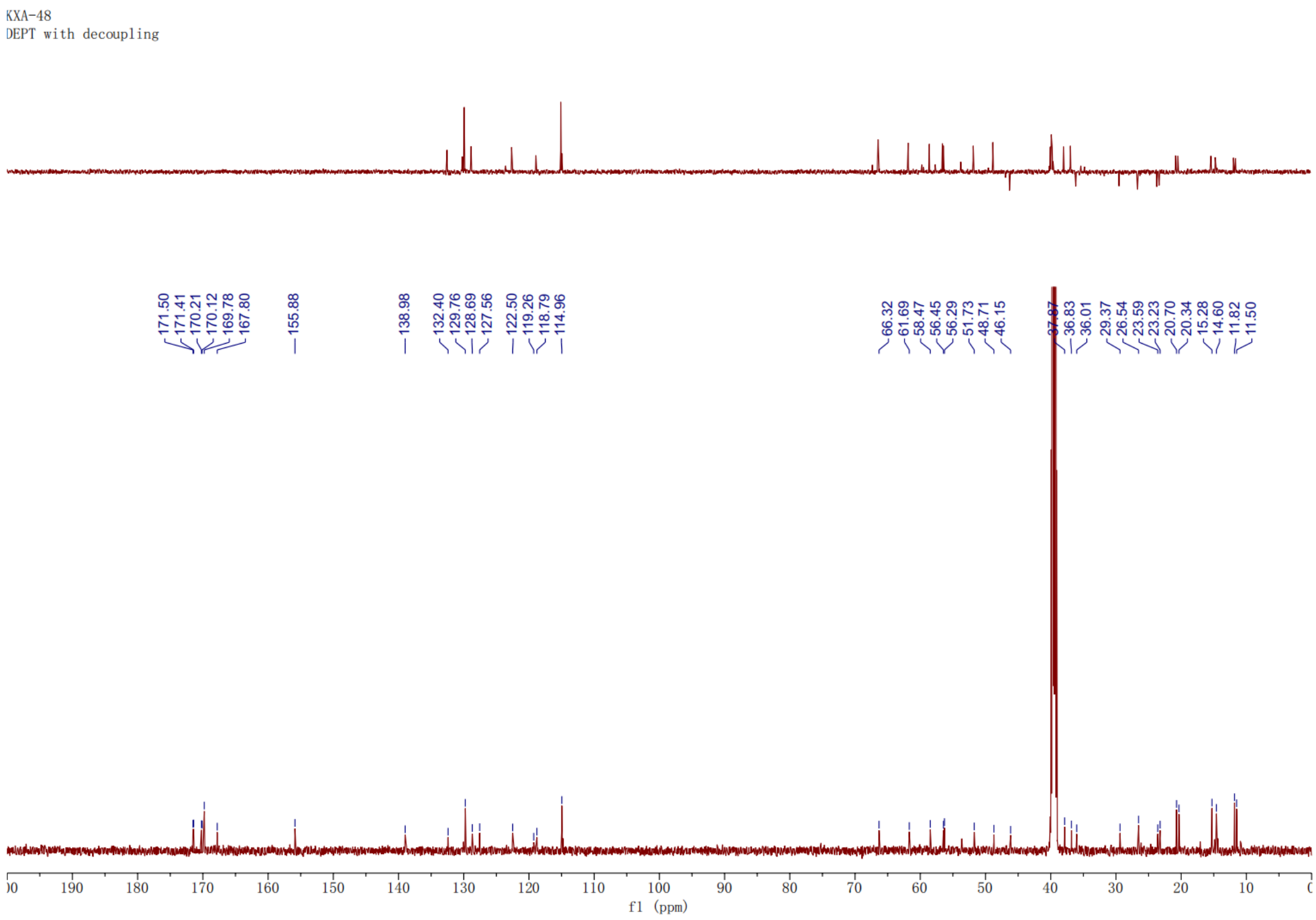

Figure S55. ^{13}C NMR (151 MHz) spectrum of compound **17** in DMSO-d_6.

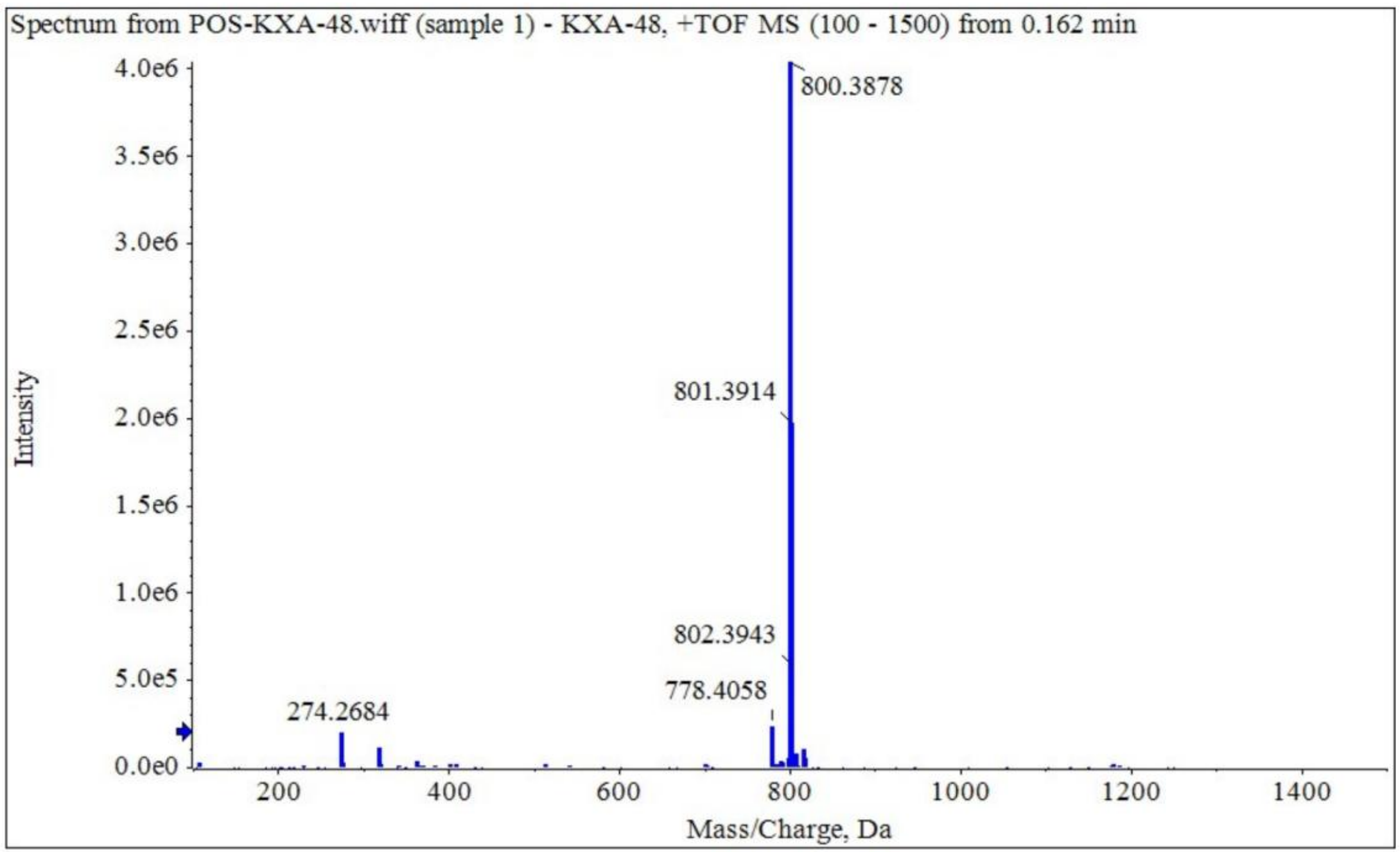

Figure S56. The positive HR-ESI-MS spectrum of compound **17**.

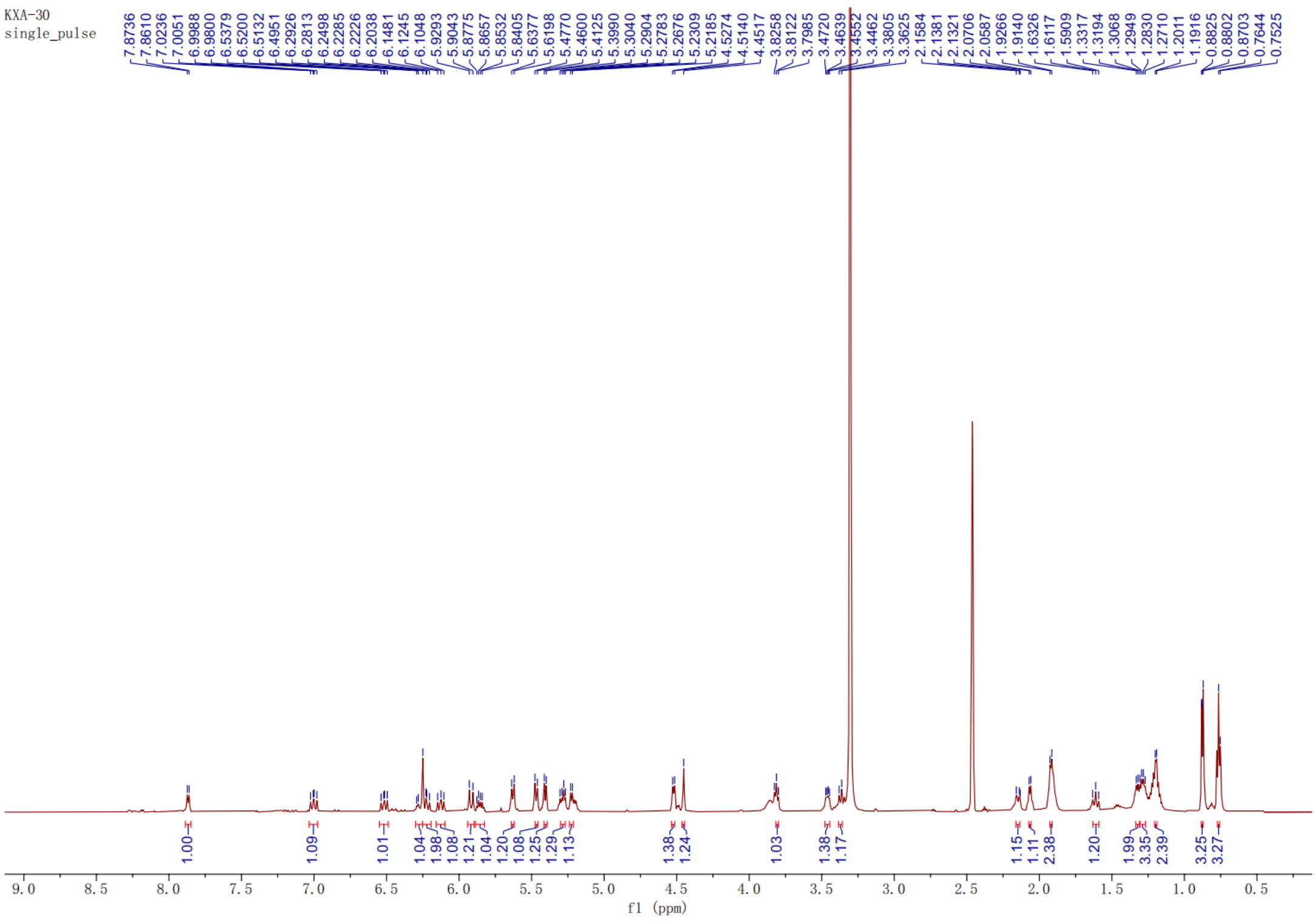

Figure S57. ^{1}H NMR (600 MHz) spectrum of compound **18** in DMSO-d_6.

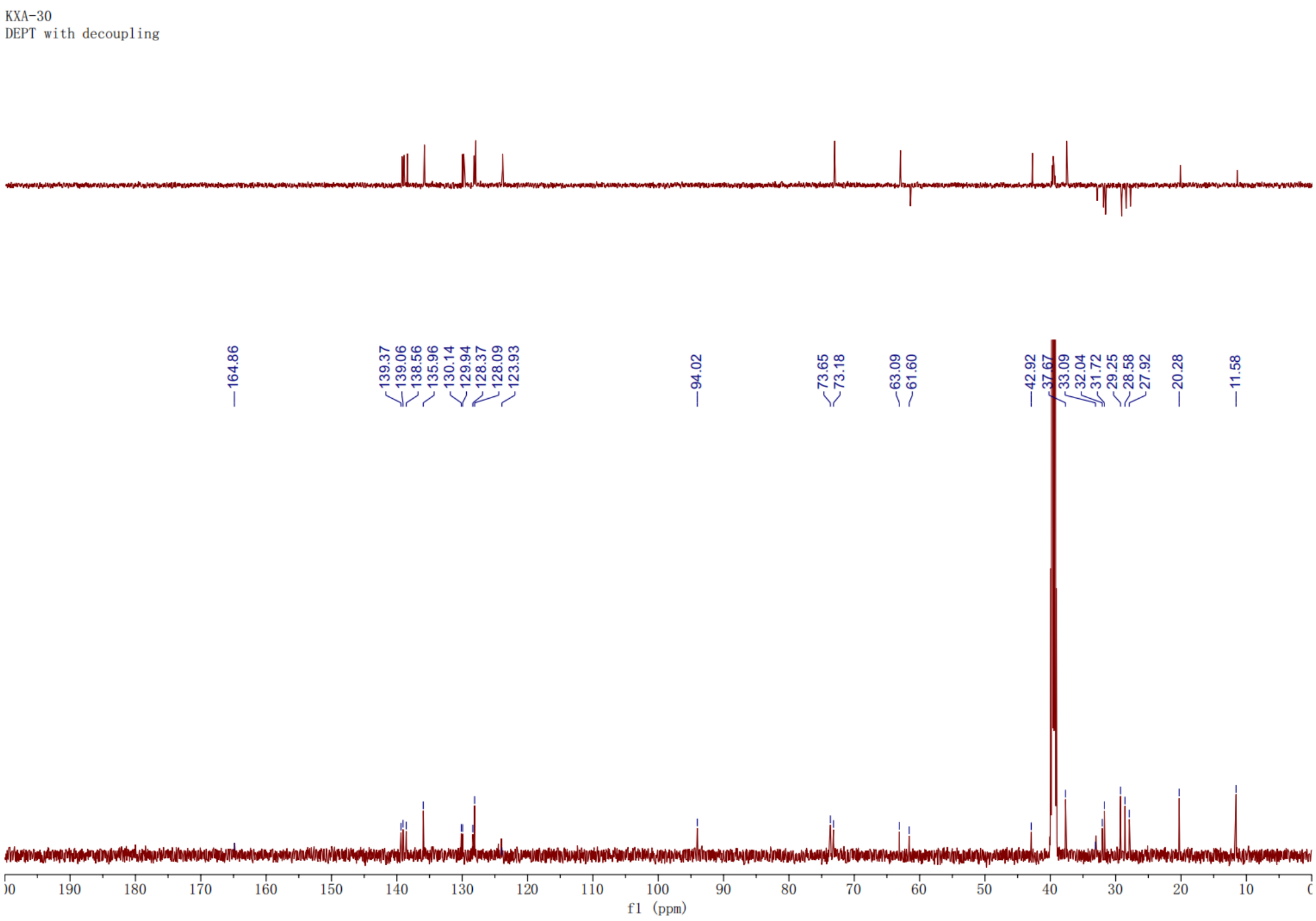

Figure S58. ^{13}C NMR (151 MHz) spectrum of compound **18** in DMSO-d_6.

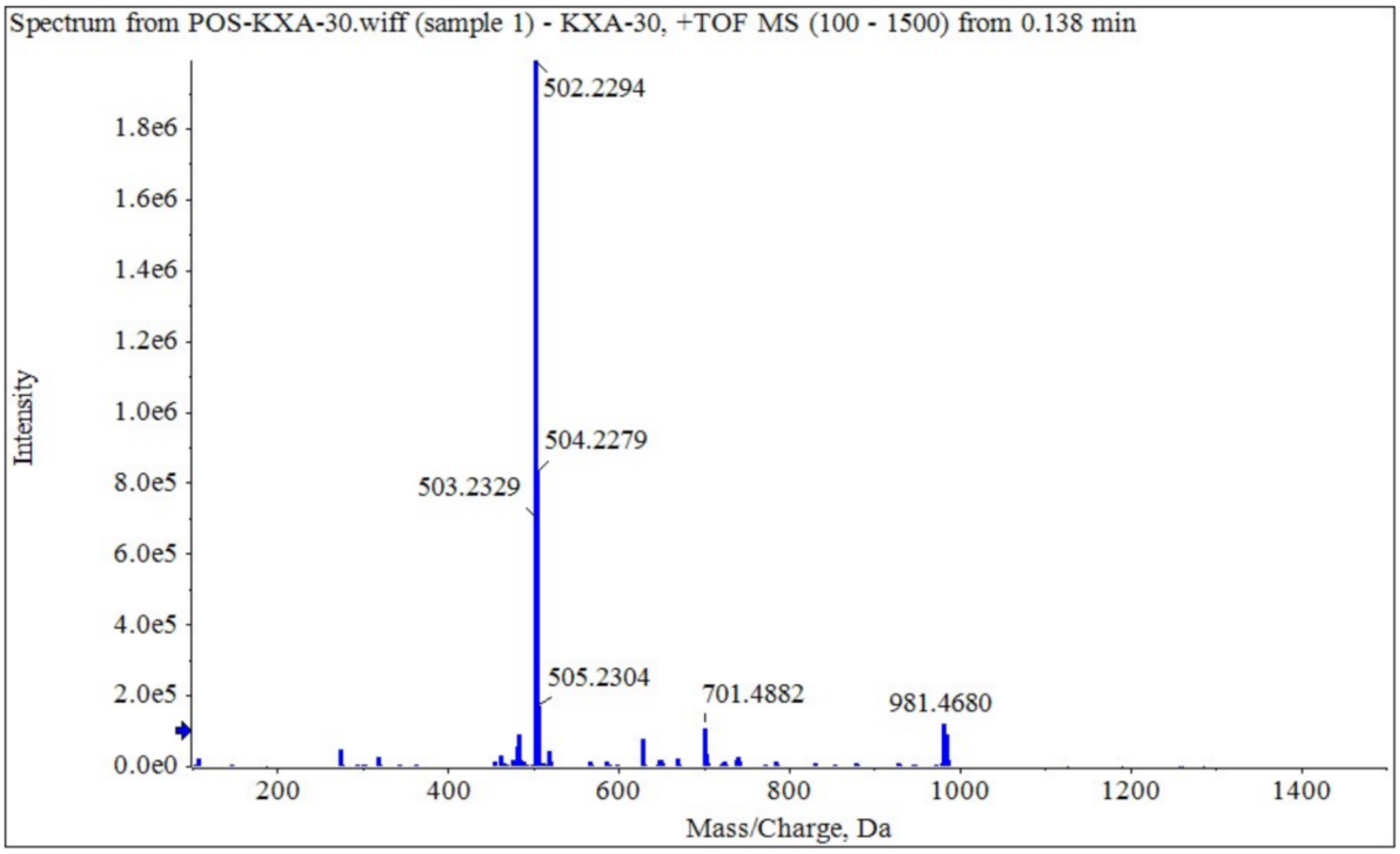

Figure S59. The positive HR-ESI-MS spectrum of compound **18**.

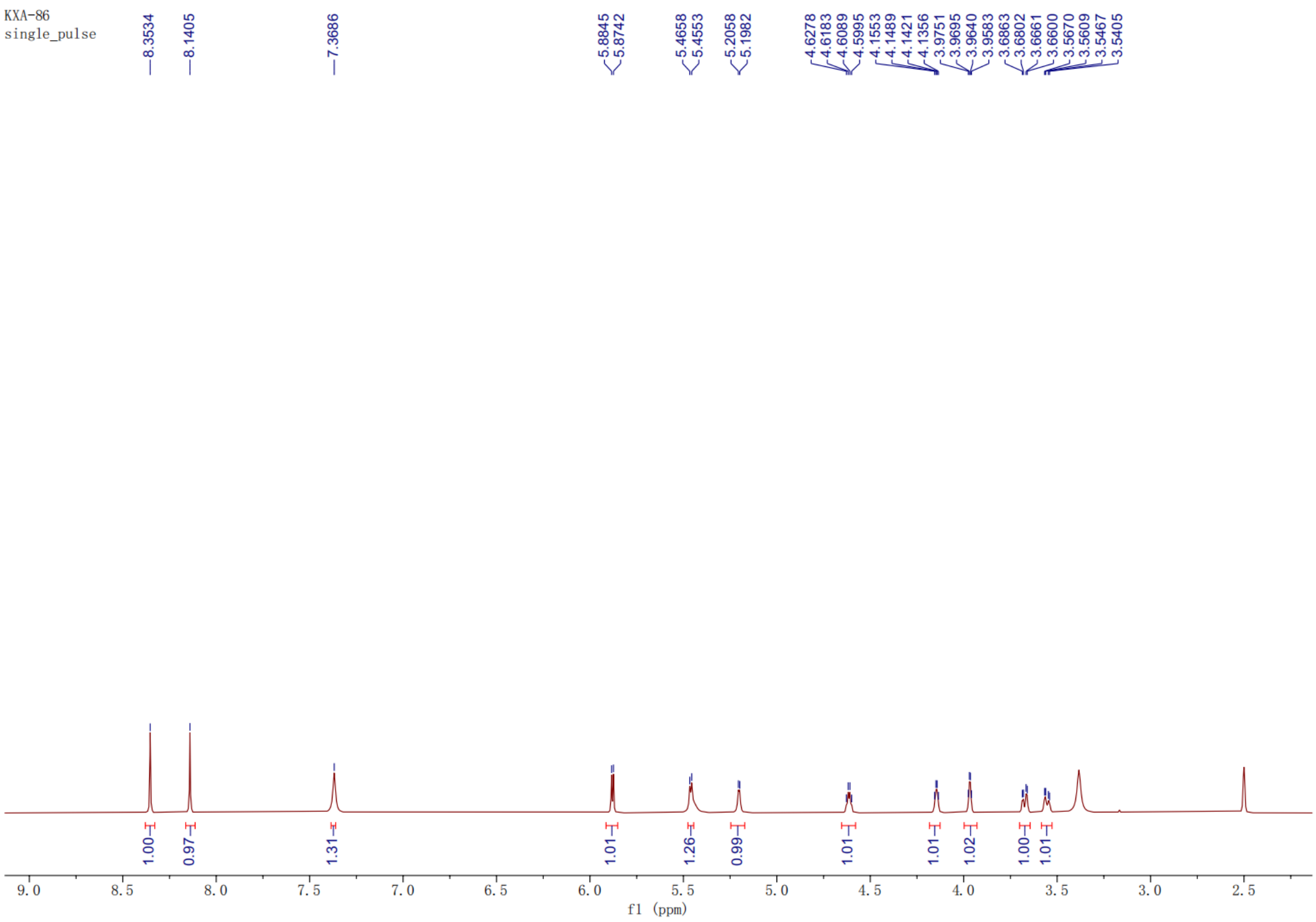

Figure S60. ^{1}H NMR (600 MHz) spectrum of compound **19** in DMSO-d_6.

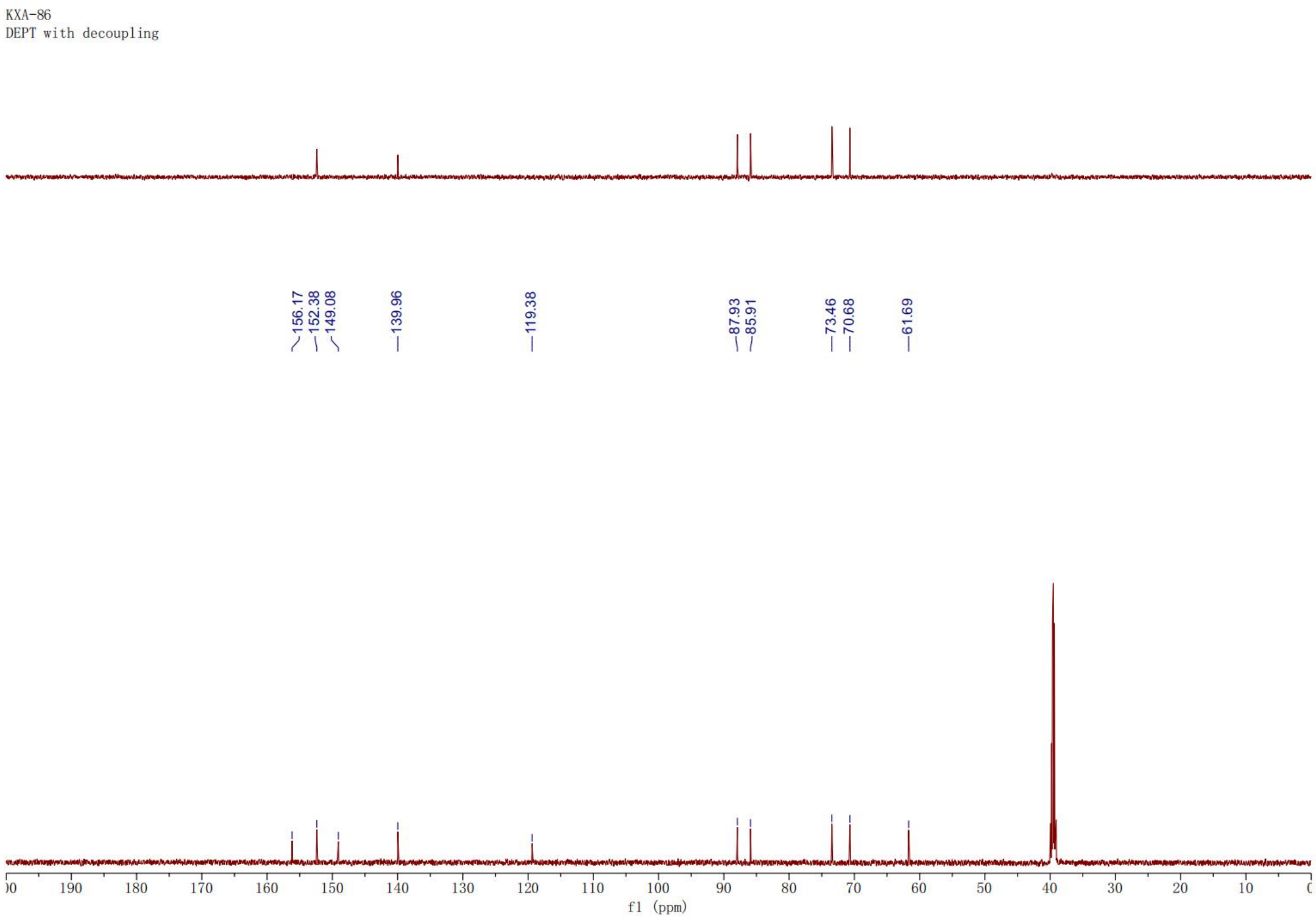

Figure S61. ^{13}C NMR (151 MHz) spectrum of compound **19** in DMSO-d_6.

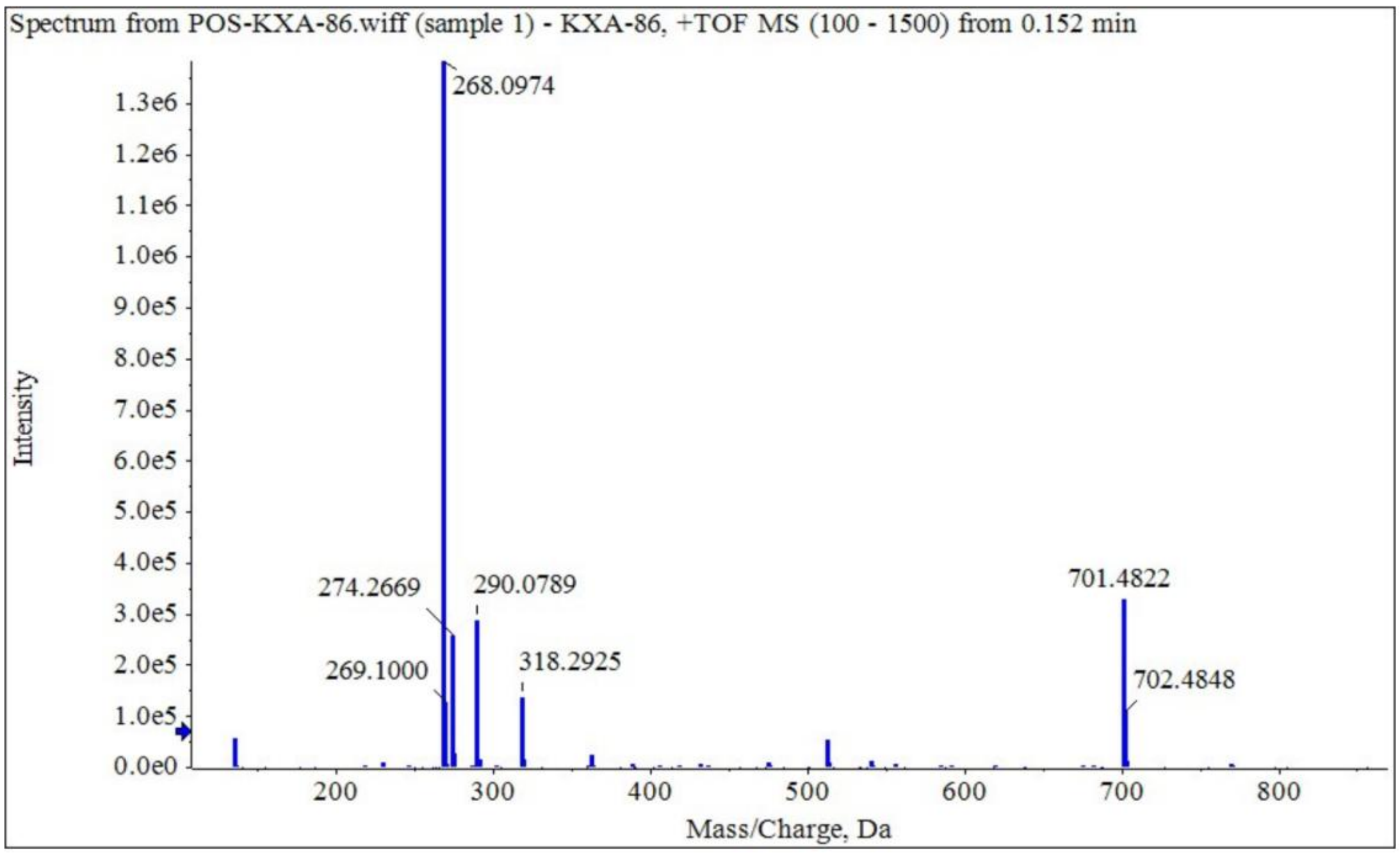

Figure S62. The positive HR-ESI-MS spectrum of compound **19**.

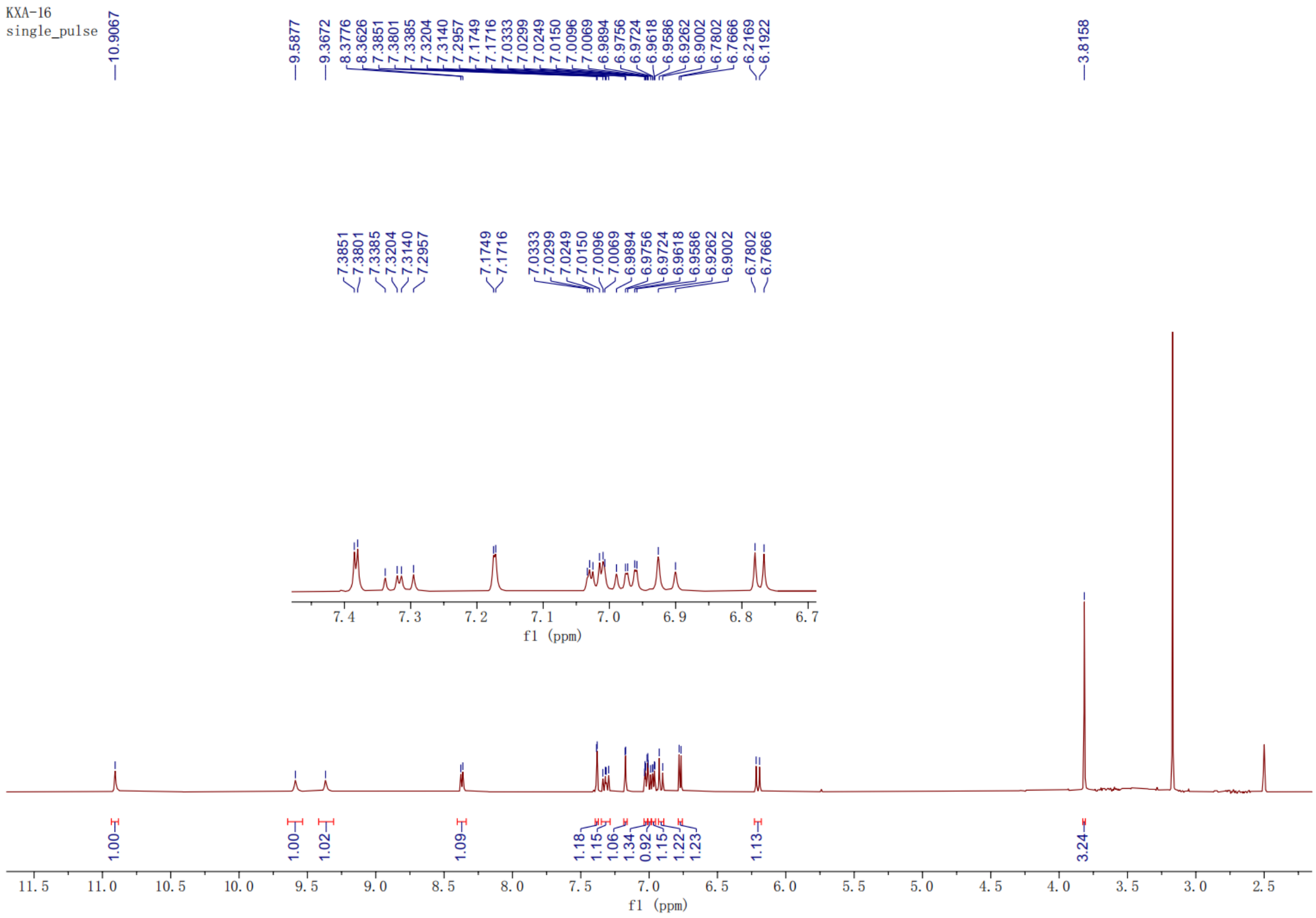

Figure S63. ^{1}H NMR (600 MHz) spectrum of compound **20** in DMSO-d_6.

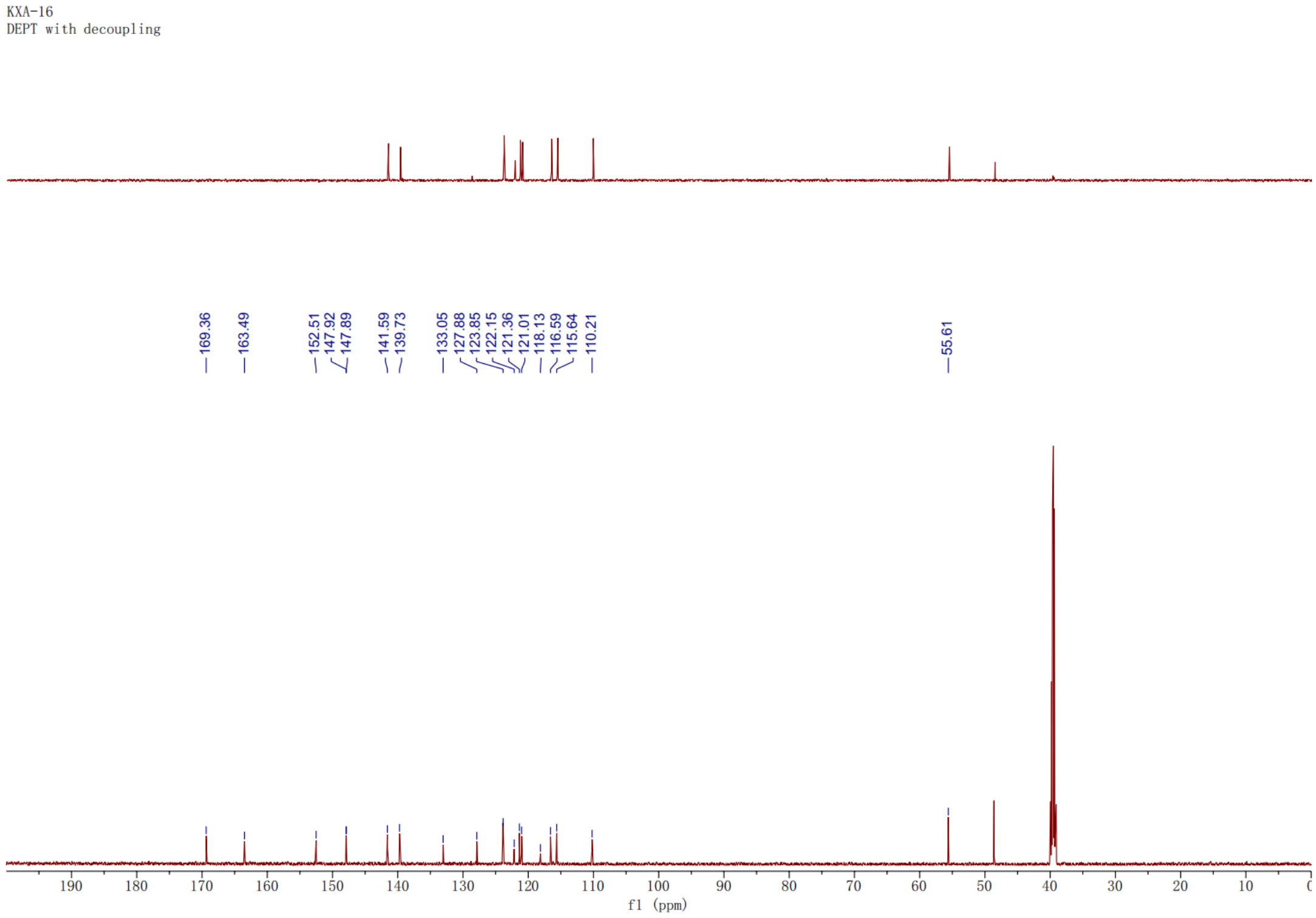

Figure S64. ^{13}C NMR (151 MHz) spectrum of compound **20** in DMSO-d_6.

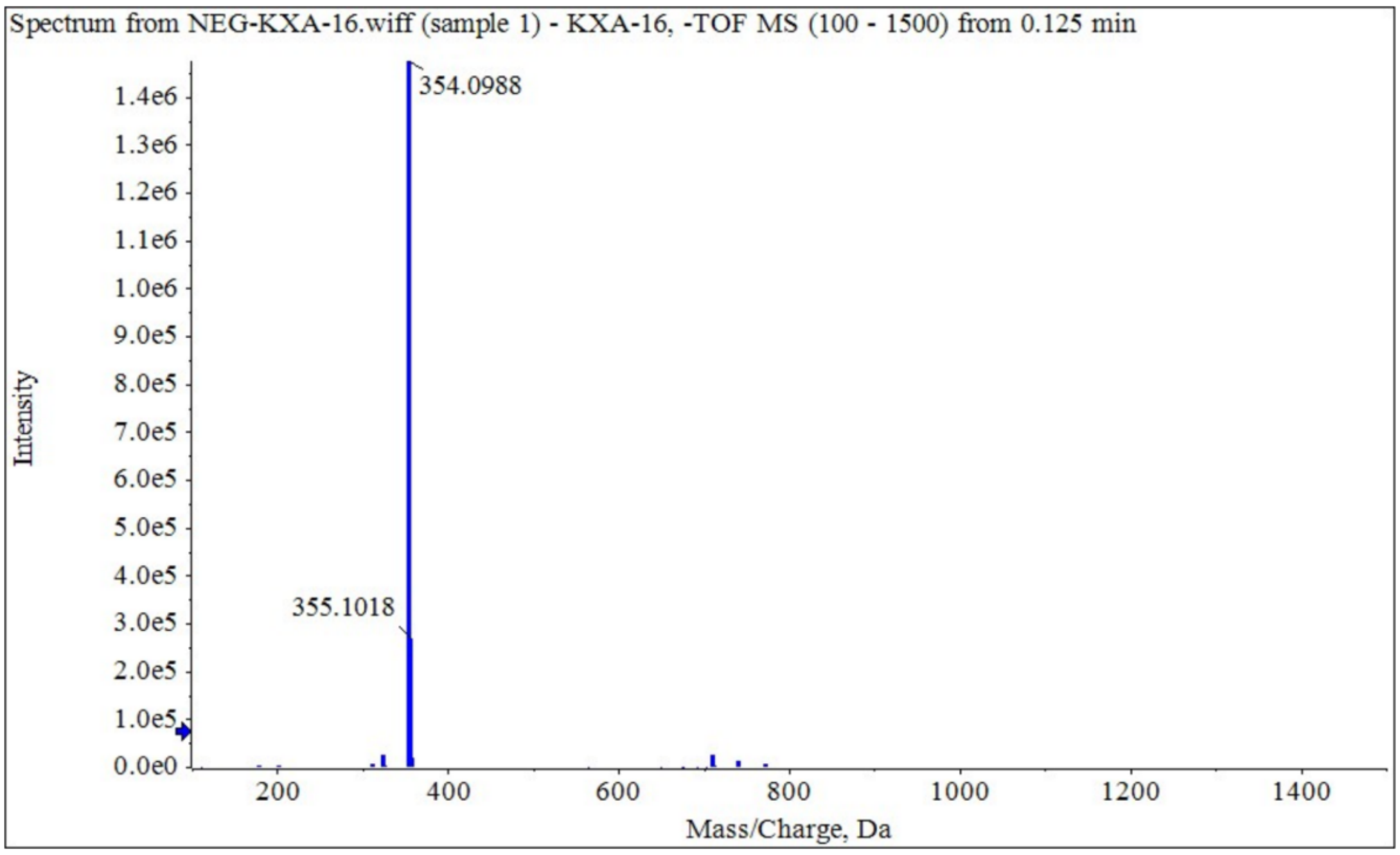

Figure S65. The negative HR-ESI-MS spectrum of compound **20**.

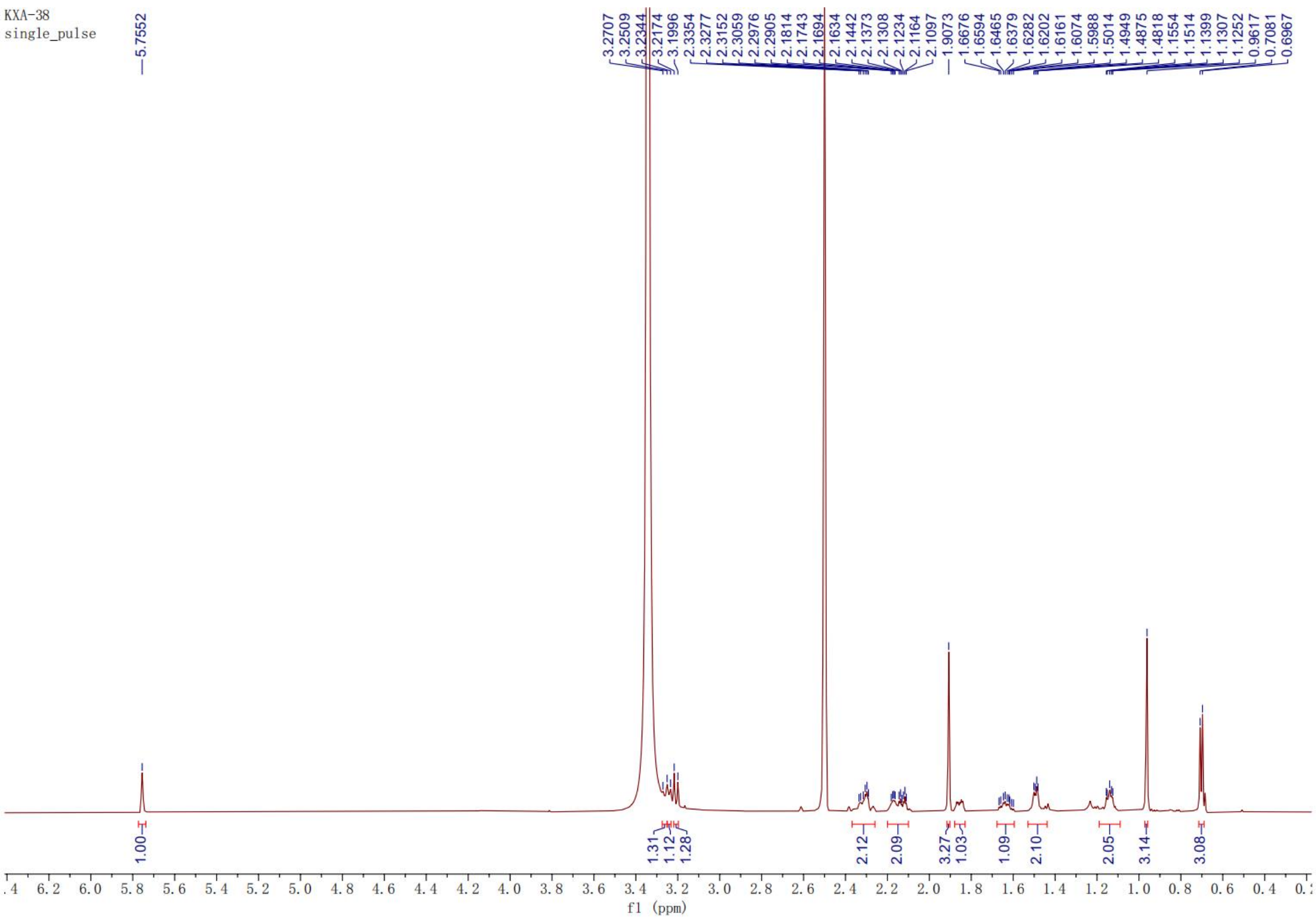

Figure S66. ^{1}H NMR (600 MHz) spectrum of compound **21** in DMSO-d_6.

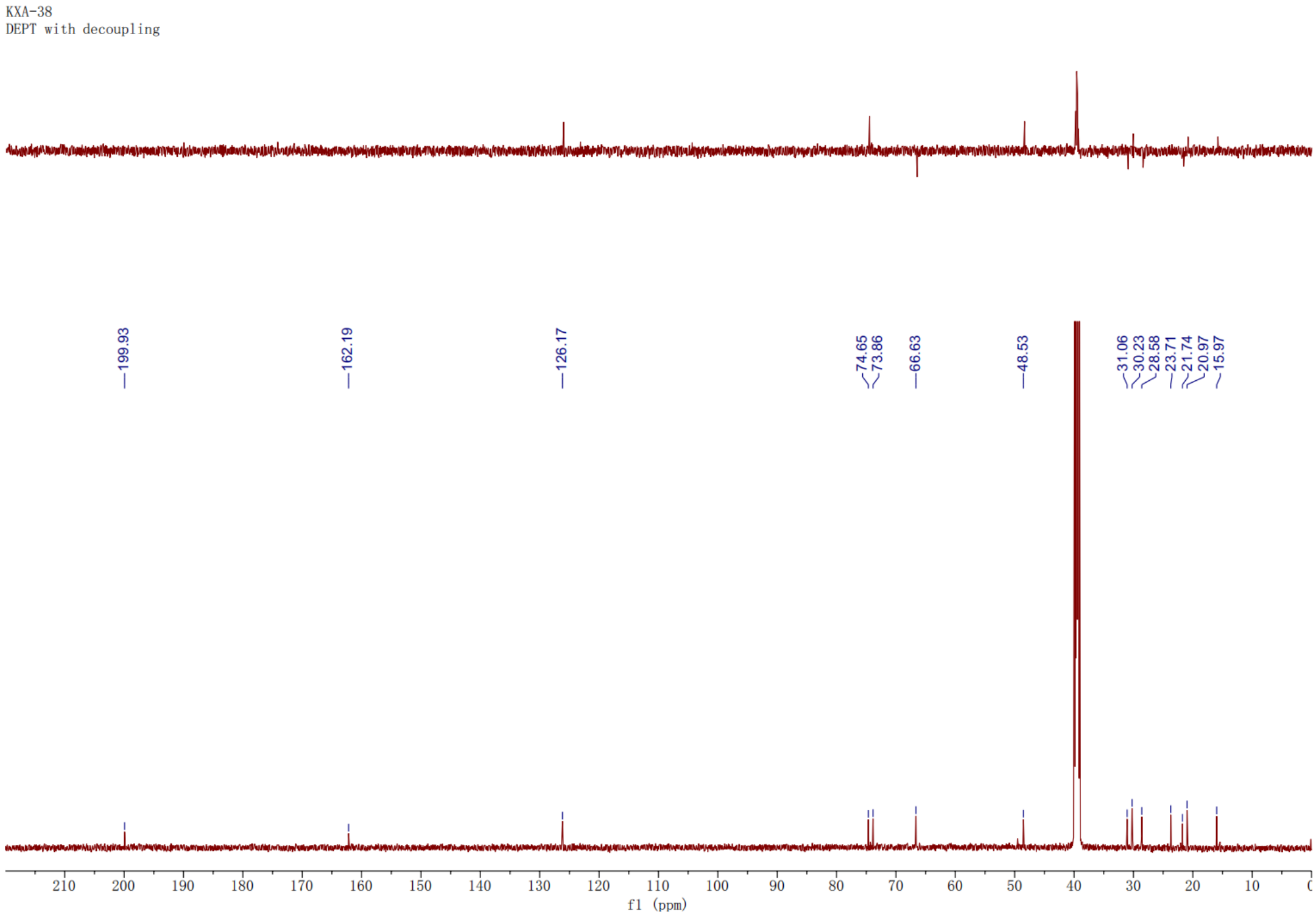

Figure S67. ^{13}C NMR (151 MHz) spectrum of compound **21** in DMSO-d_6.

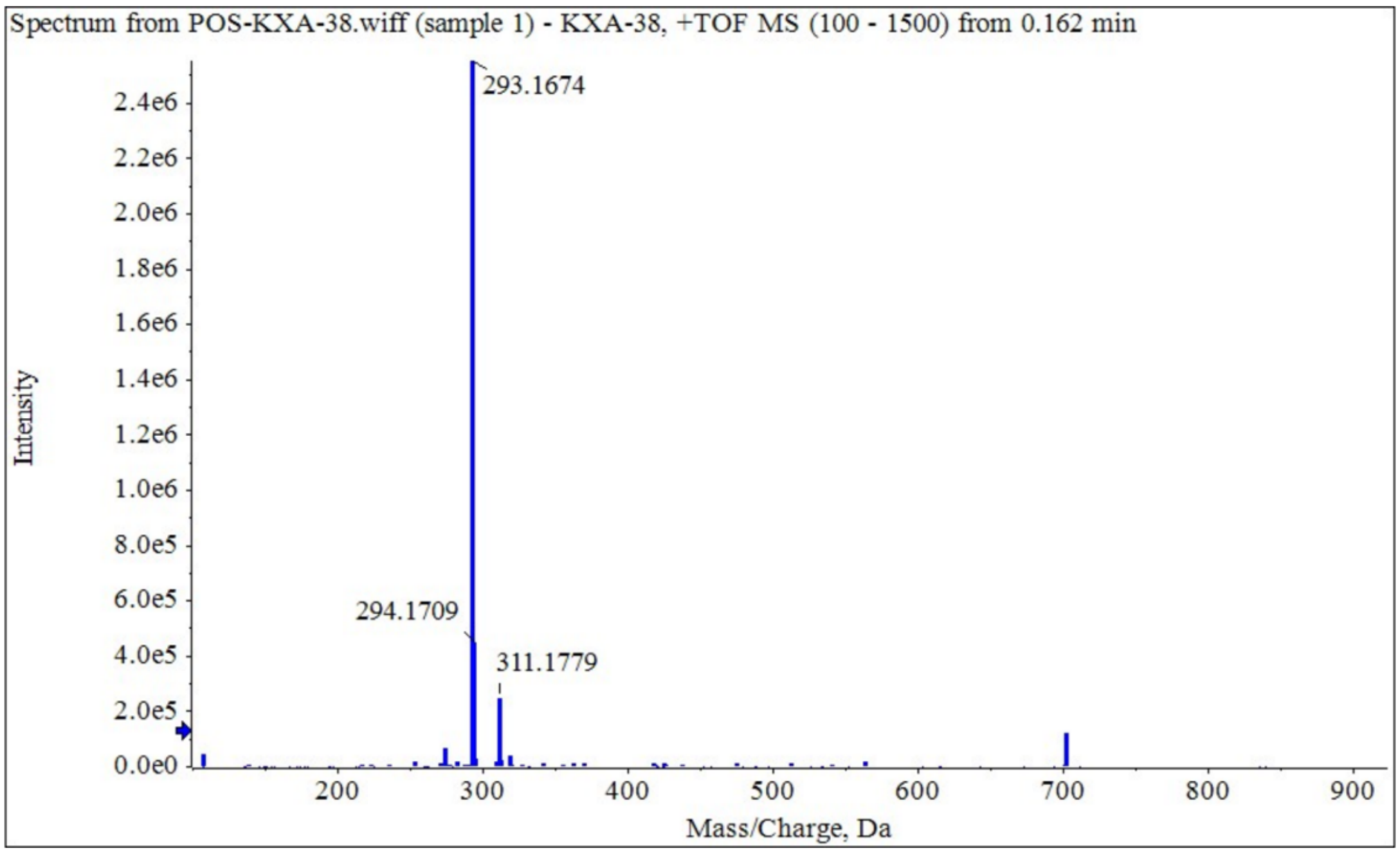

Figure S68. The positive HR-ESI-MS spectrum of compound **21**.

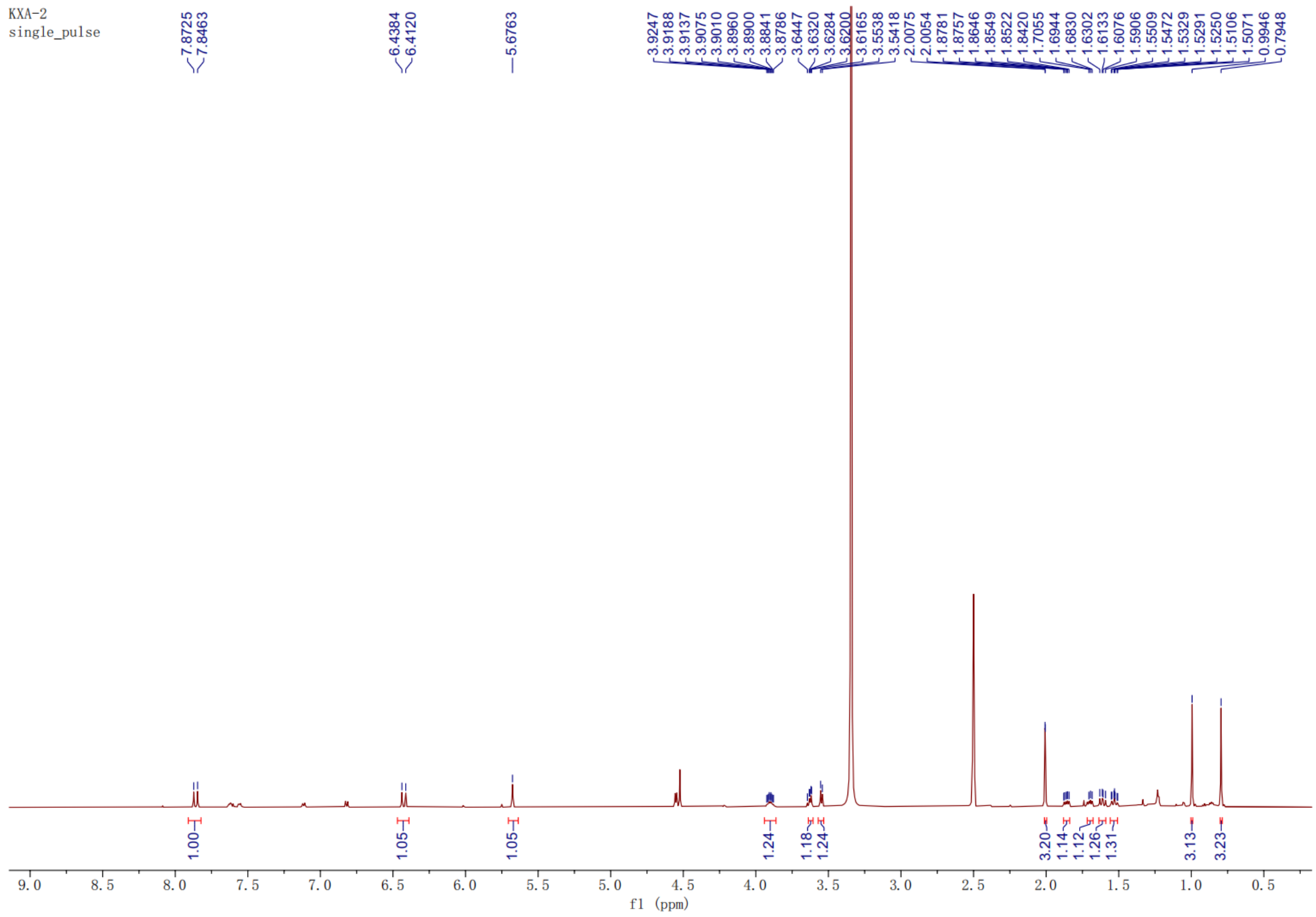

Figure S69. ^{1}H NMR (600 MHz) spectrum of compound **22** in DMSO-d_6.

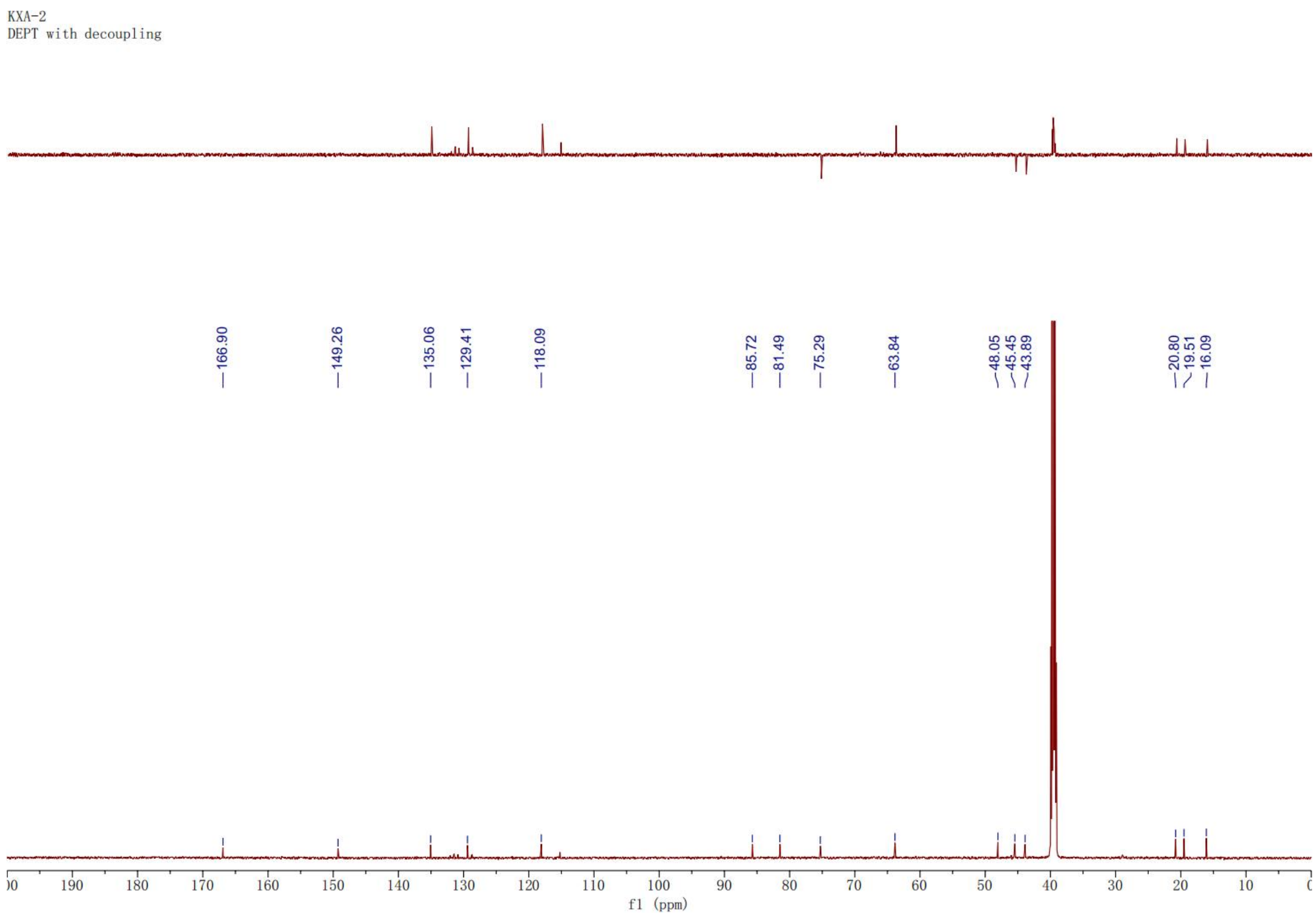

Figure S70. ^{13}C NMR (151 MHz) spectrum of compound **22** in DMSO-d_6.

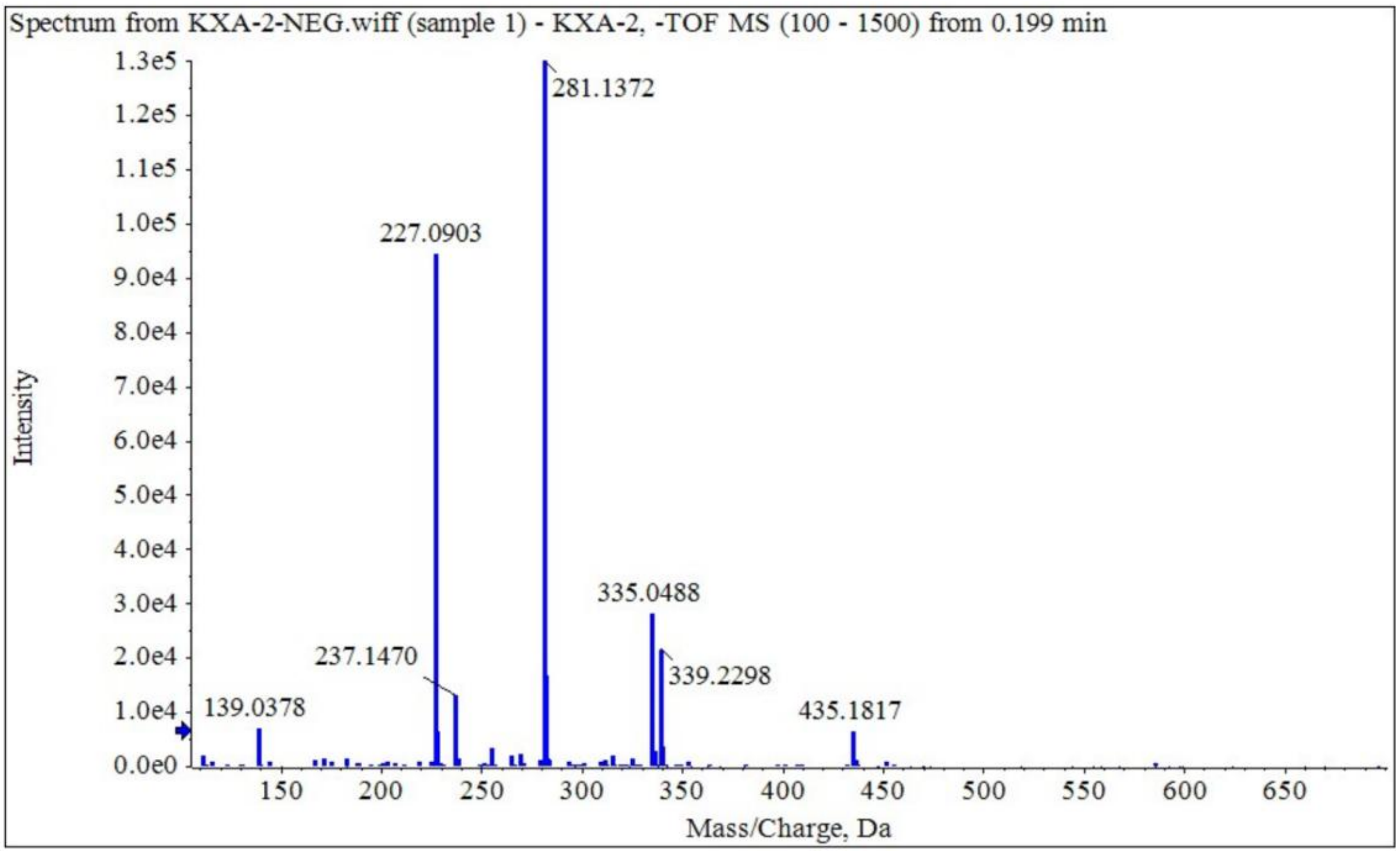

Figure S71. The negative HR-ESI-MS spectrum of compound **22**.

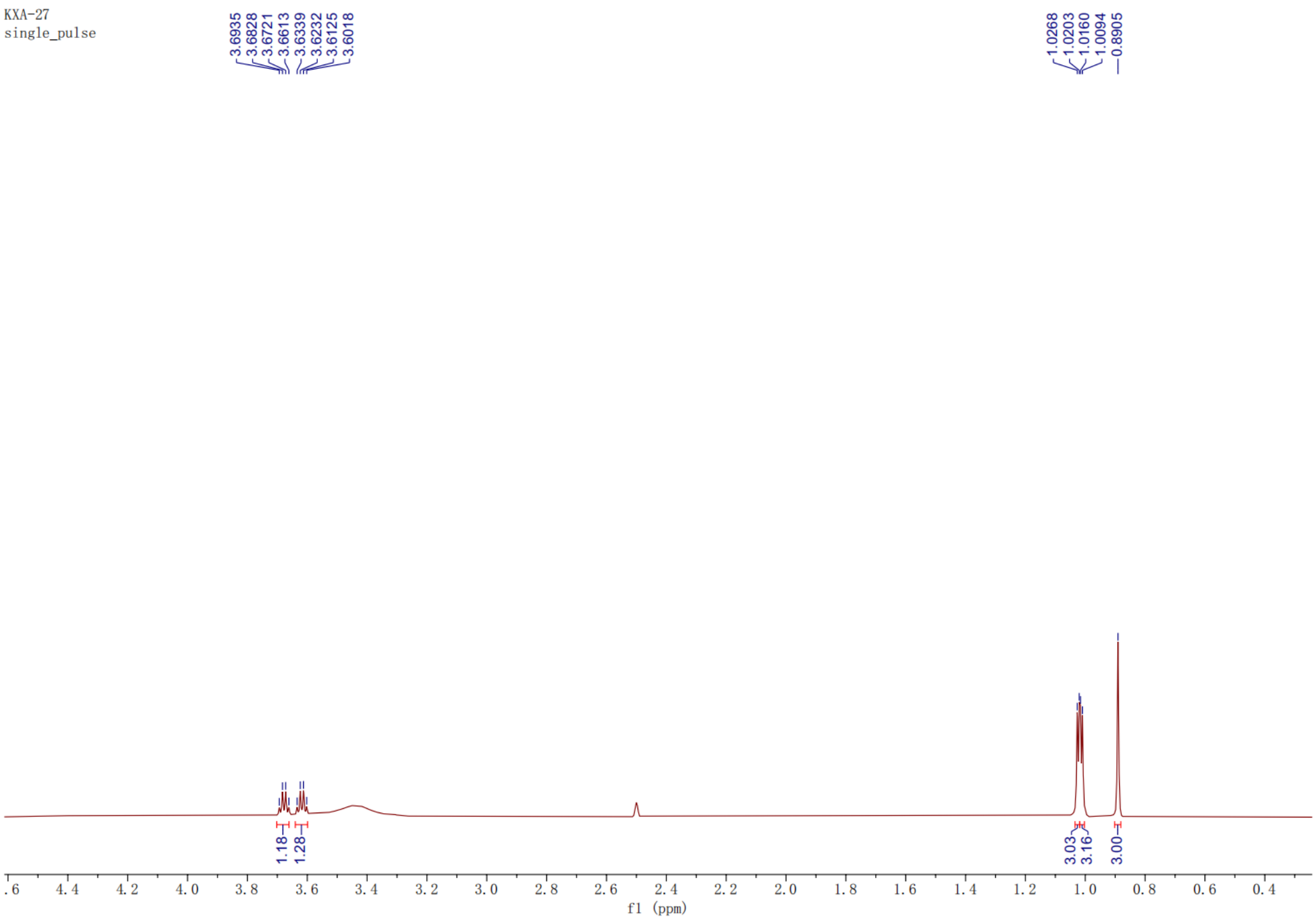

Figure S72. ^{1}H NMR (600 MHz) spectrum of compound **23** in DMSO-d_6.

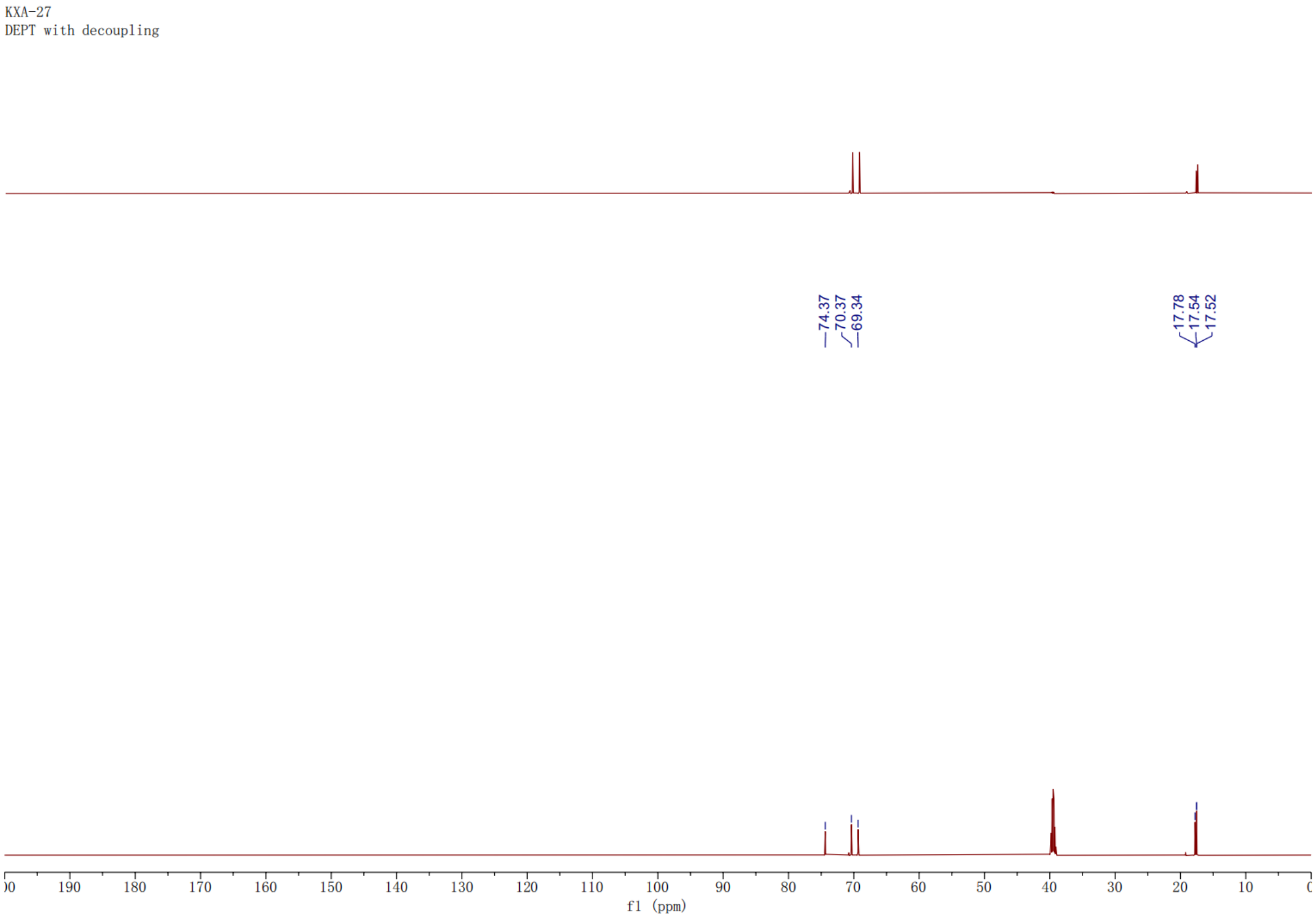

Figure S73. ^{13}C NMR (151 MHz) spectrum of compound **23** in DMSO-d_6.

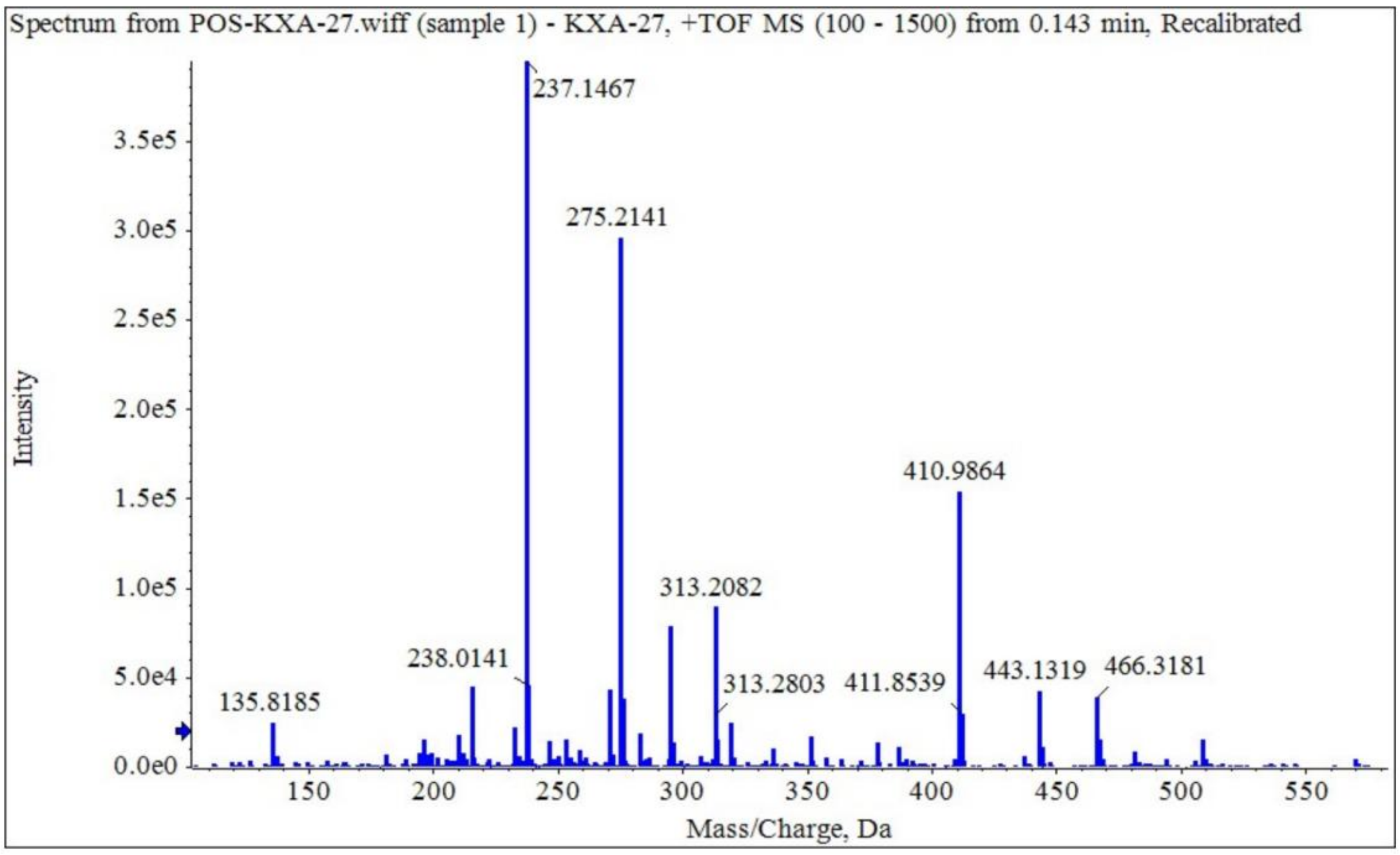

Figure S74. The positive HR-ESI-MS spectrum of compound **23**.

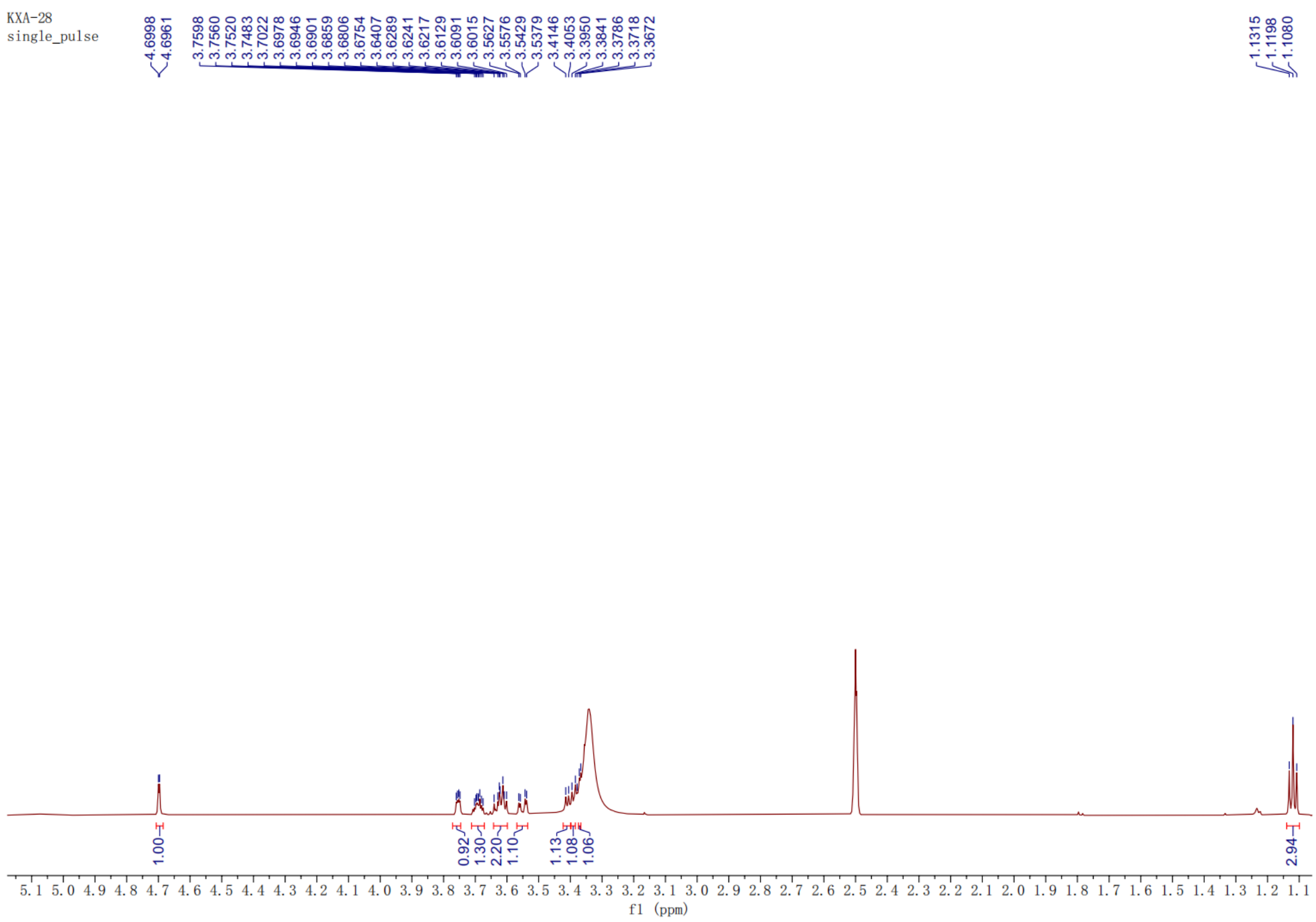

Figure S75. ^{1}H NMR (600 MHz) spectrum of compound **24** in DMSO-d_6.

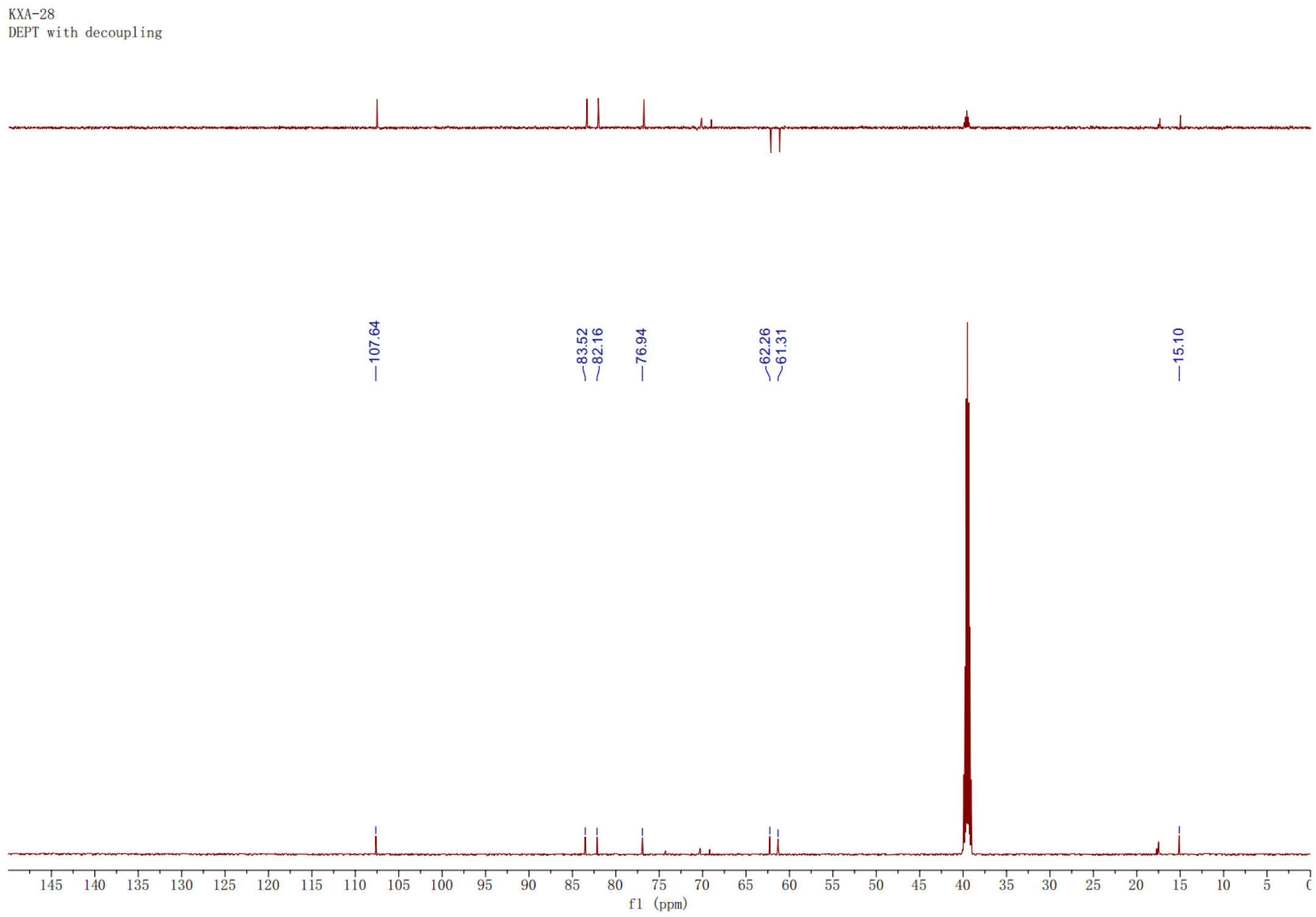

Figure S76. ^{13}C NMR (151 MHz) spectrum of compound **24** in DMSO-d_6.

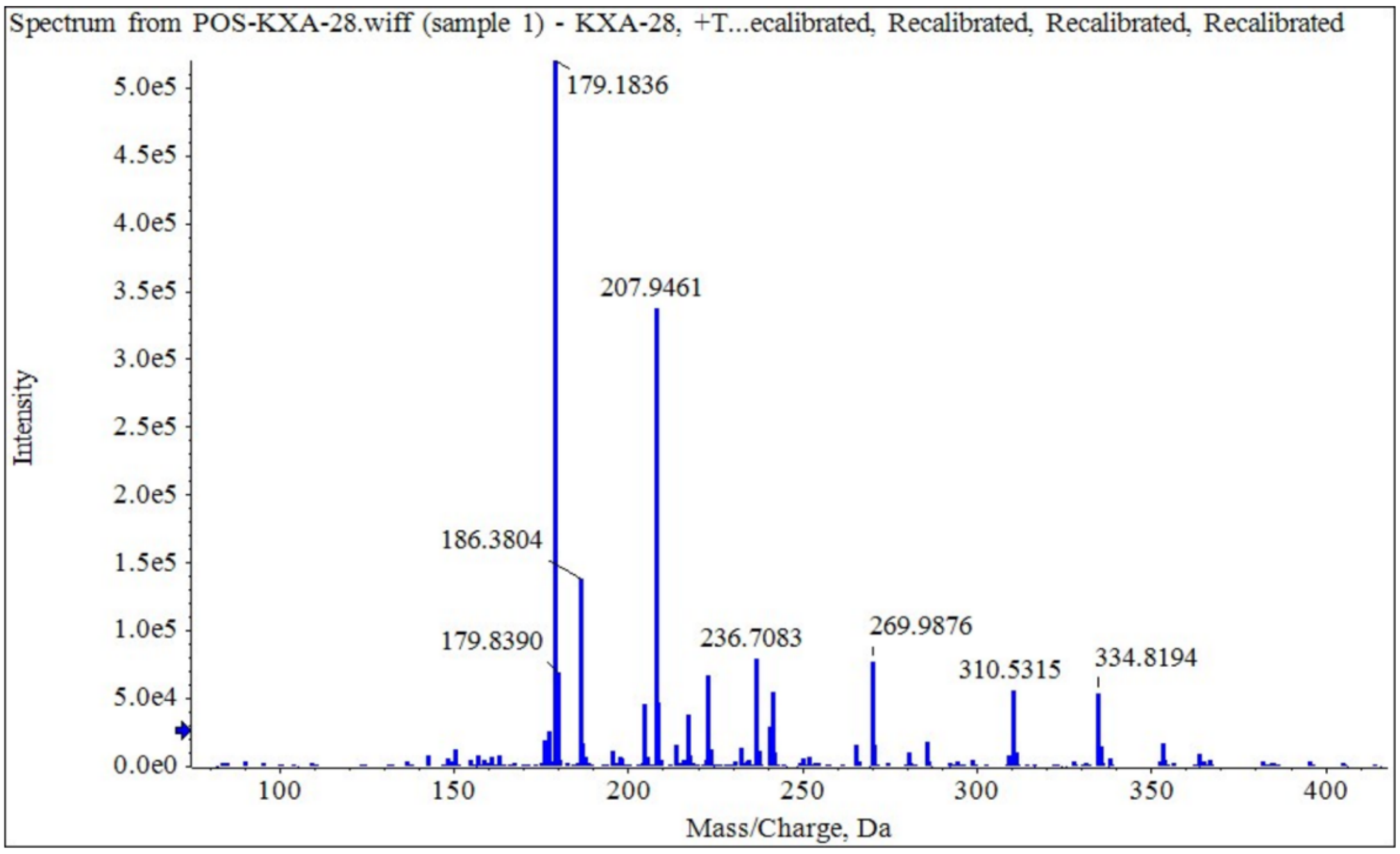

Figure S77. The positive HR-ESI-MS spectrum of compound **24**.

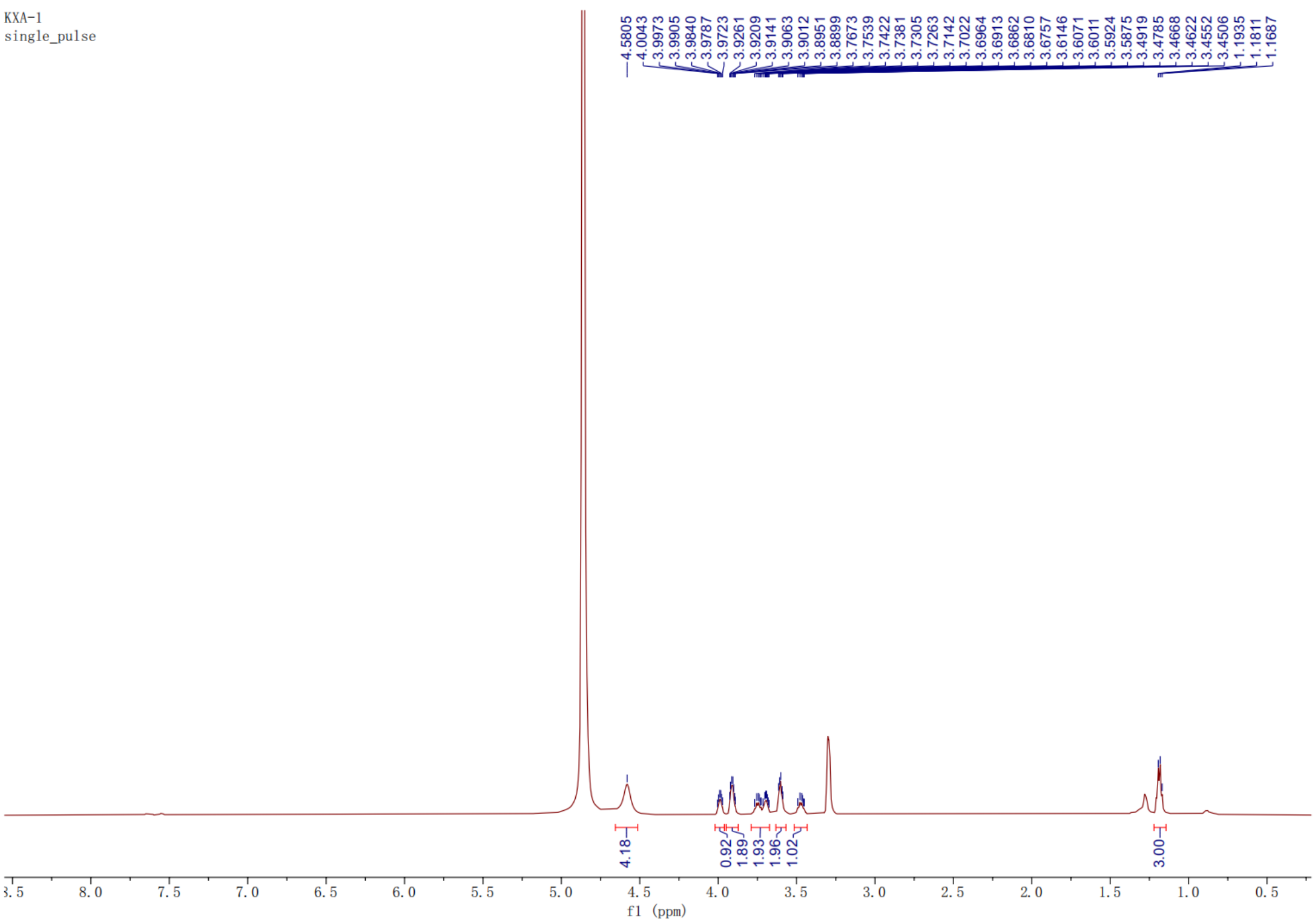

Figure S78. ^{1}H NMR (600 MHz) spectrum of compound **25** in CD_3OD.

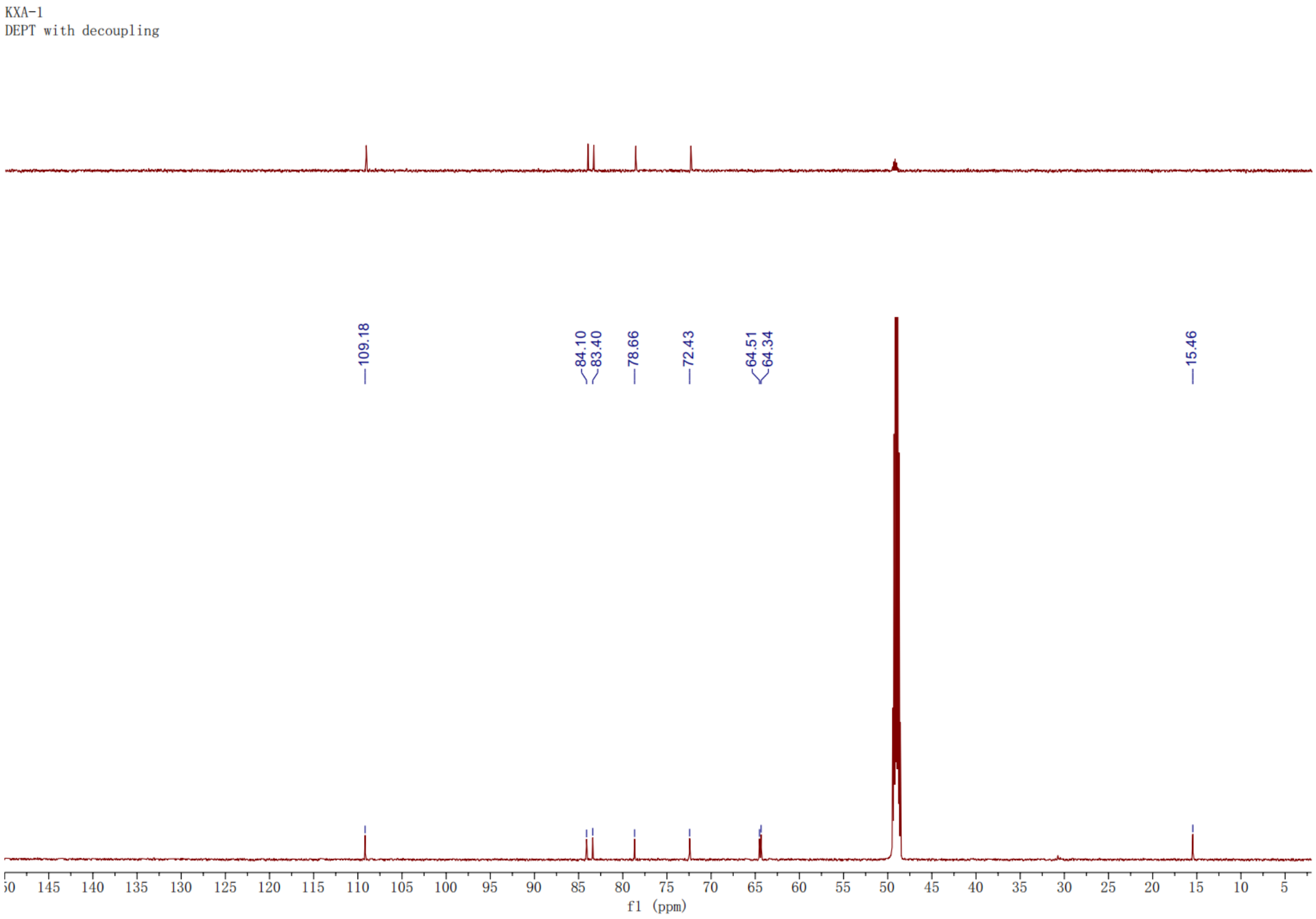

Figure S79. ^{13}C NMR (151 MHz) spectrum of compound **25** in CD_3OD.

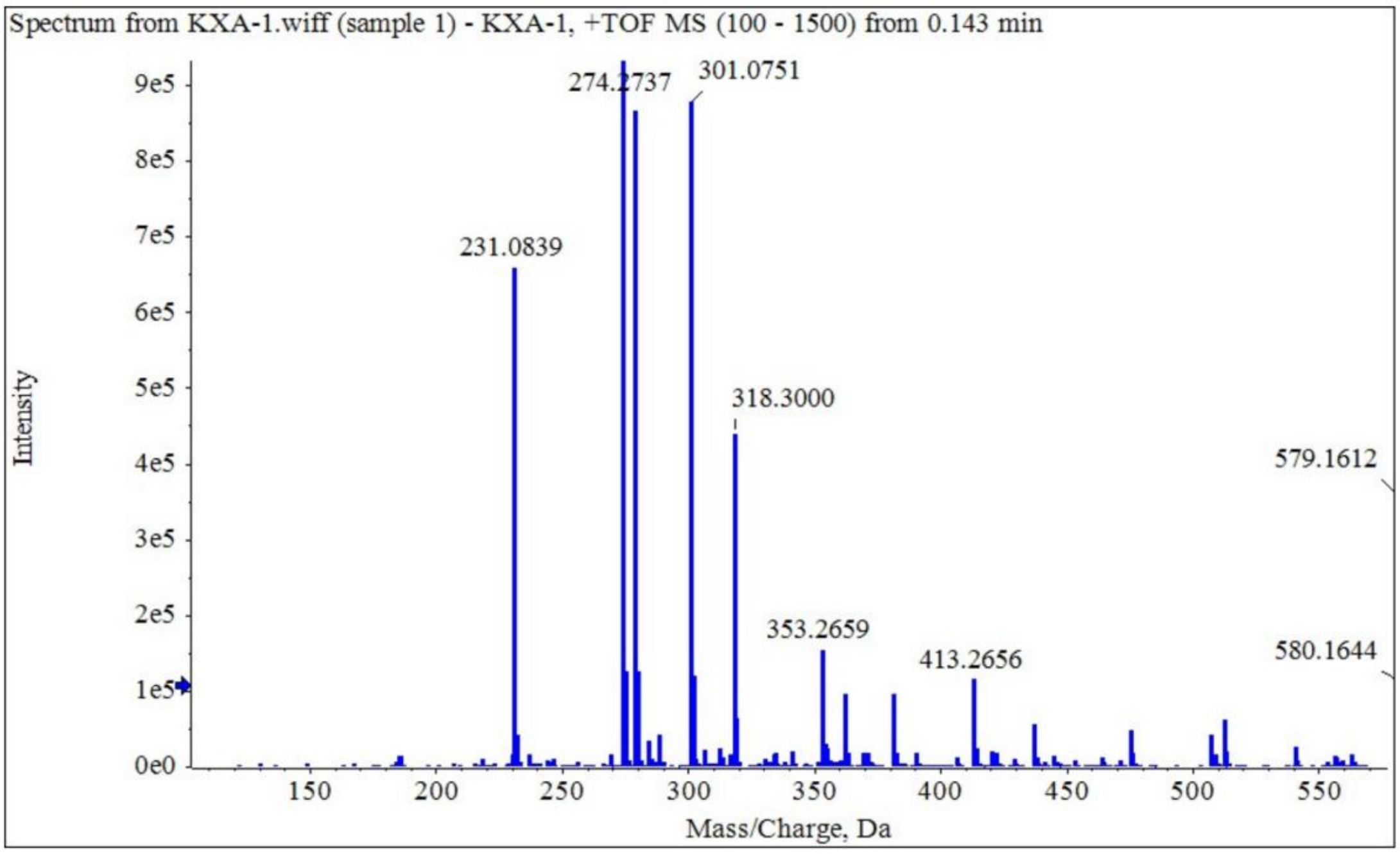

Figure S80. The positive HR-ESI-MS spectrum of compound **25**.

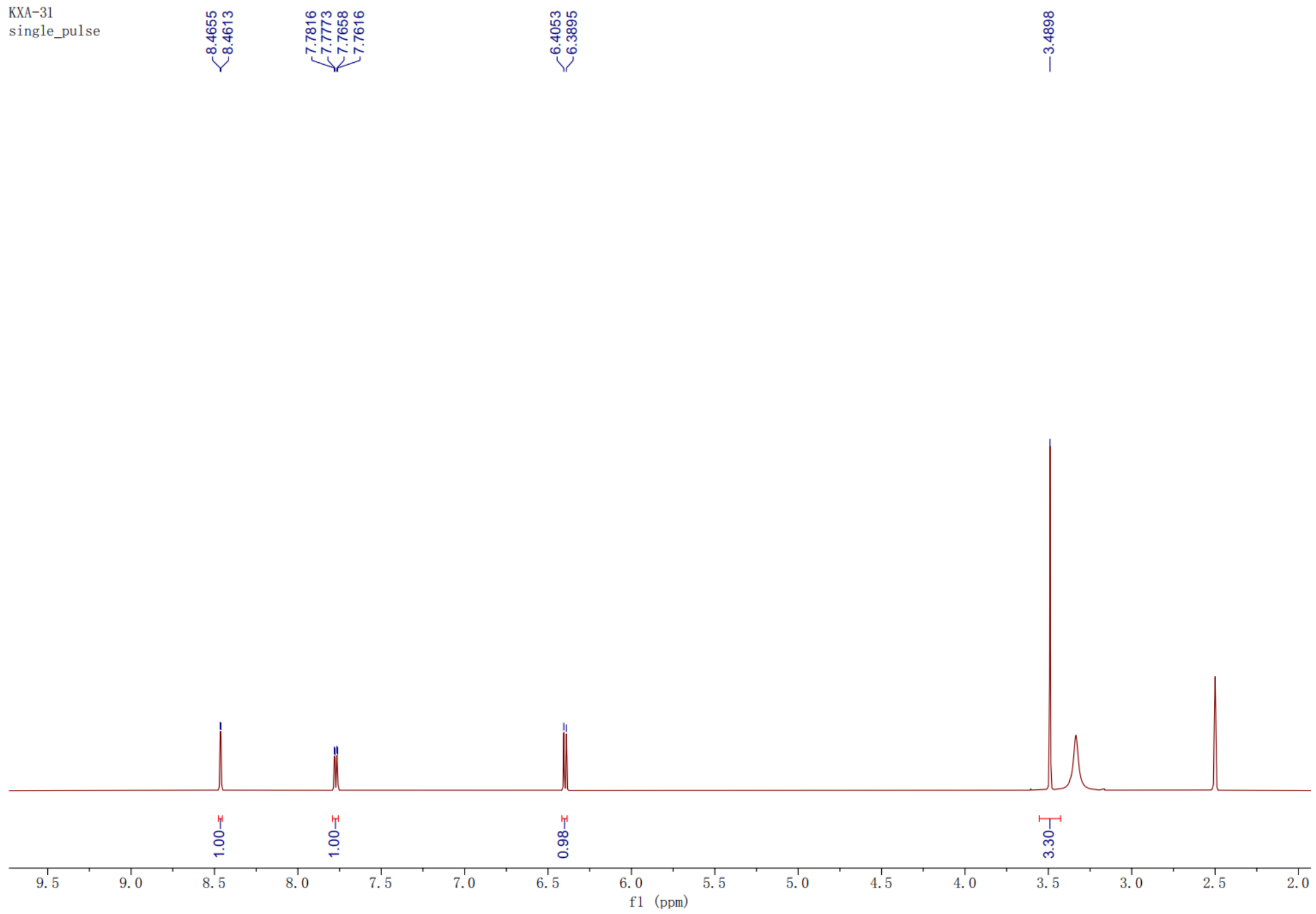

Figure S81. ^{1}H NMR (600 MHz) spectrum of compound **26** in DMSO-*d*6.

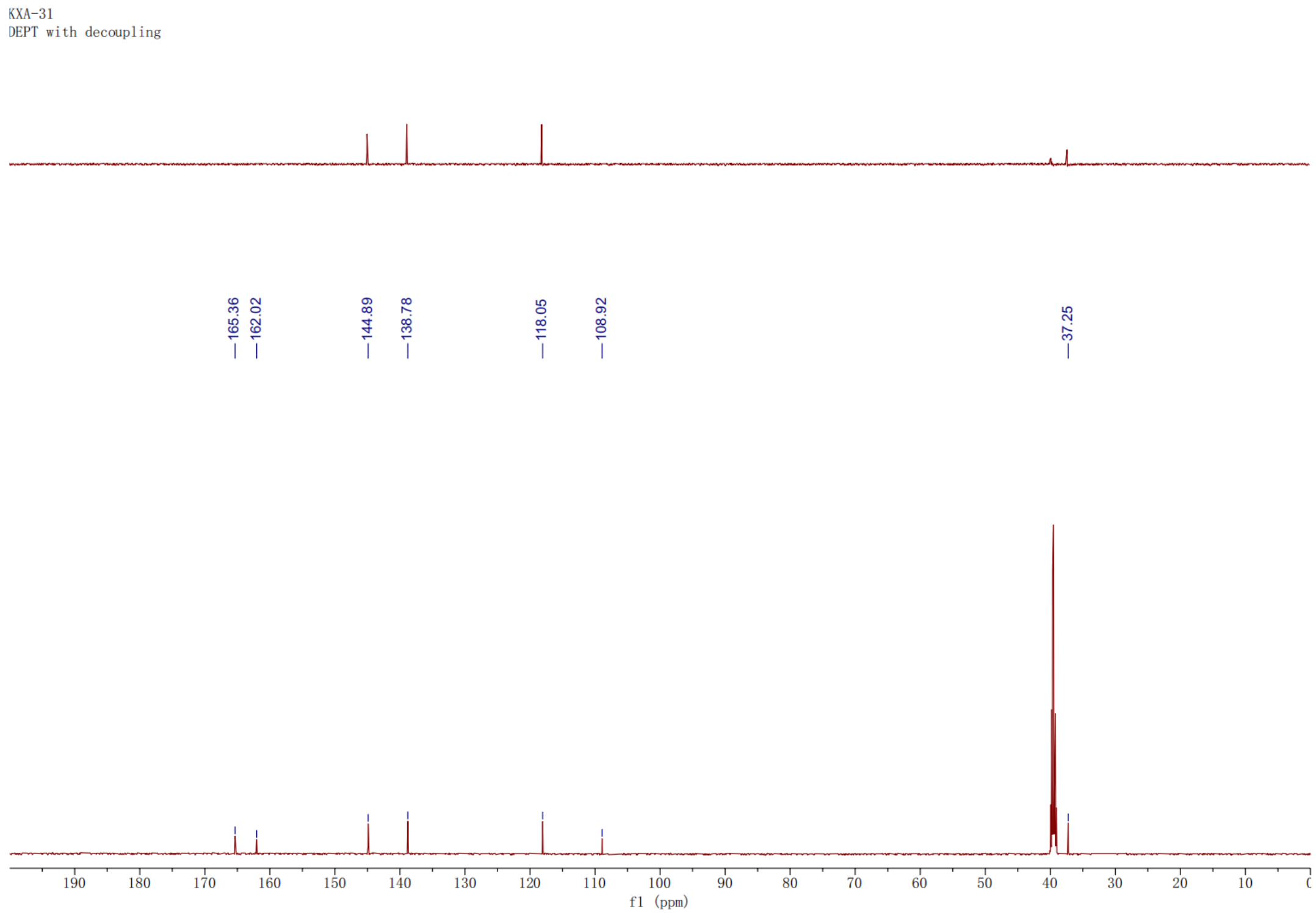

Figure S82. ^{13}C NMR (151 MHz) spectrum of compound **26** in DMSO-*d*6.

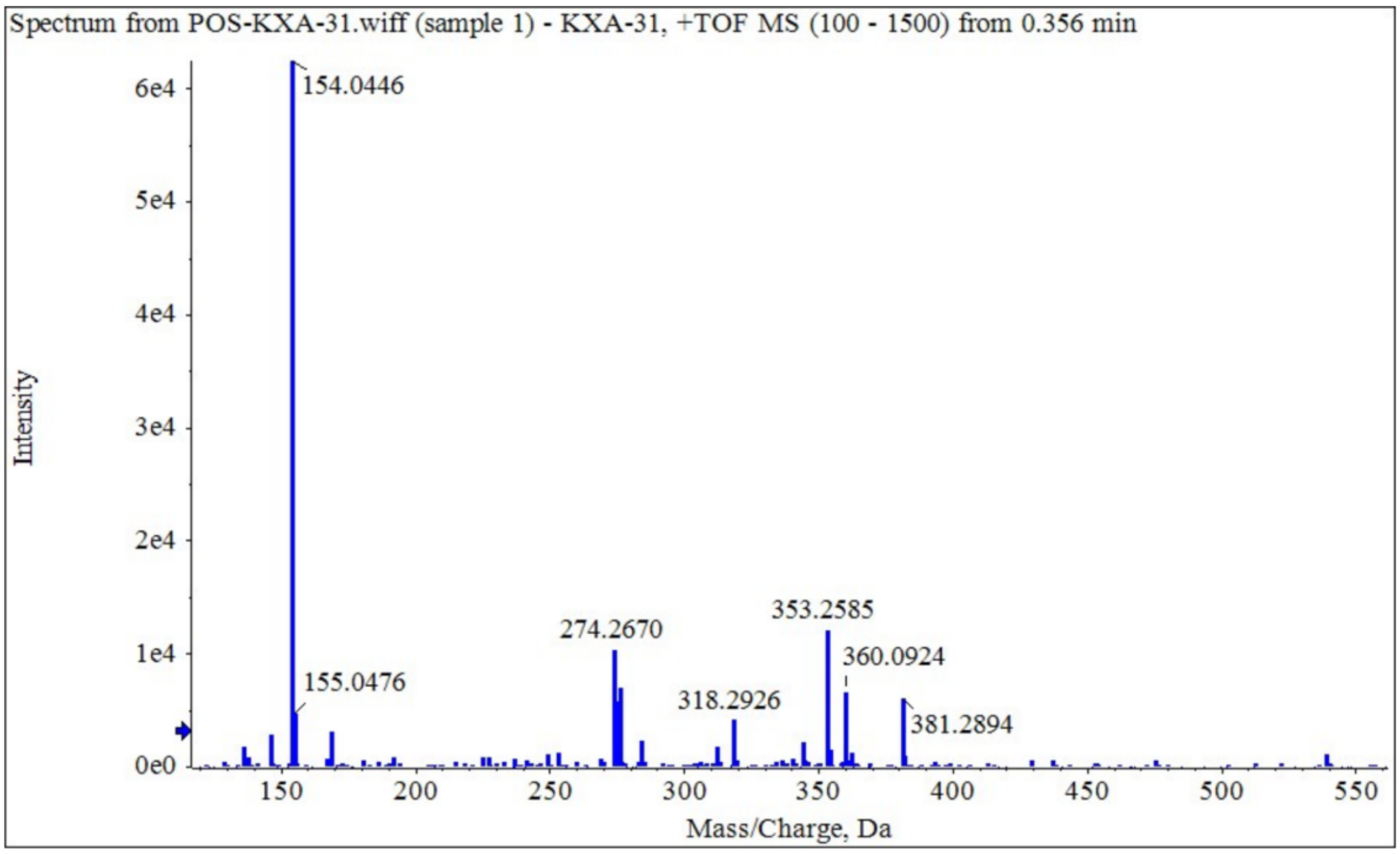

Figure S83. The positive HR-ESI-MS spectrum of compound **26**.

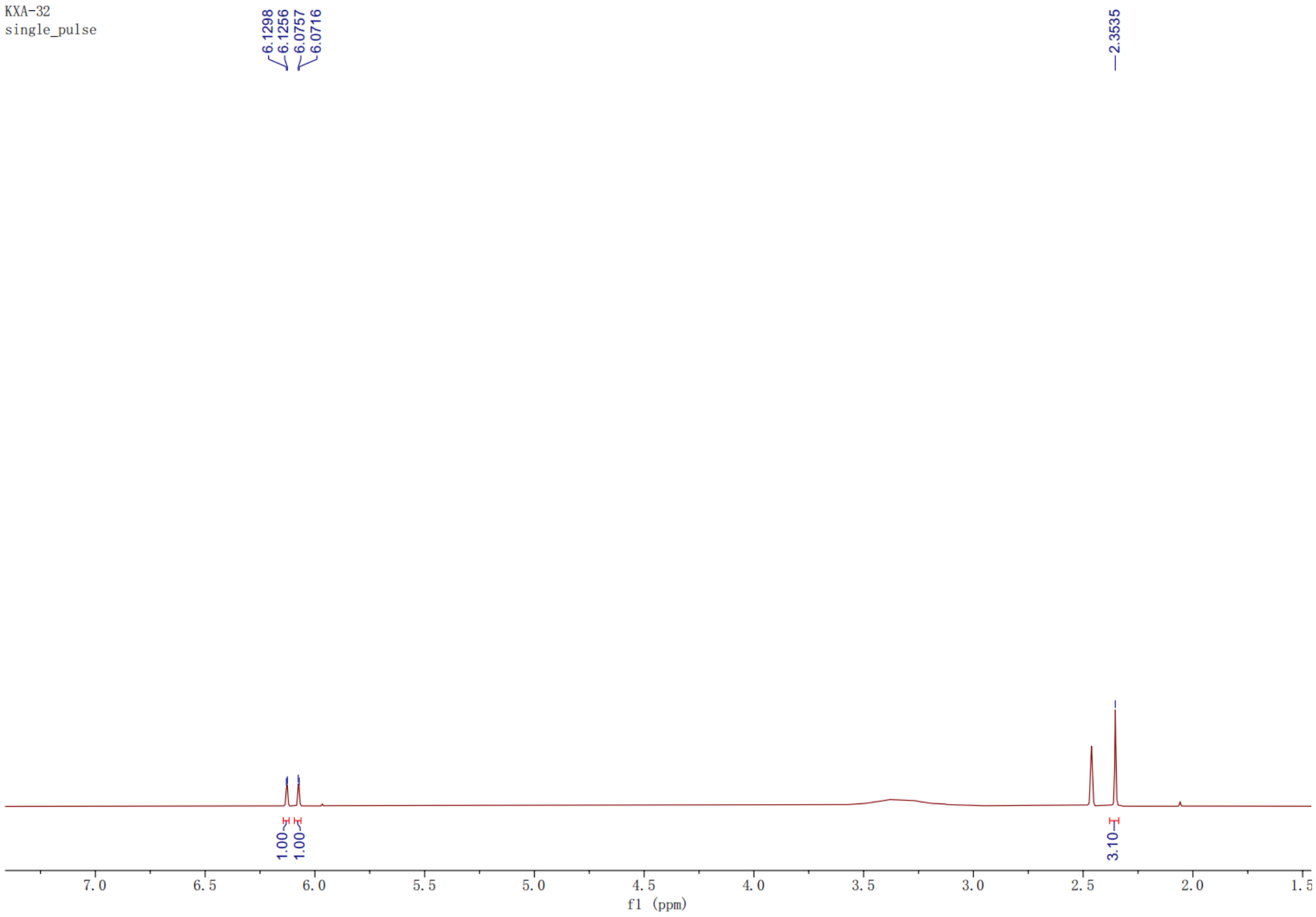

Figure S84. ^{1}H NMR (600 MHz) spectrum of compound **27** in DMSO-d_6.

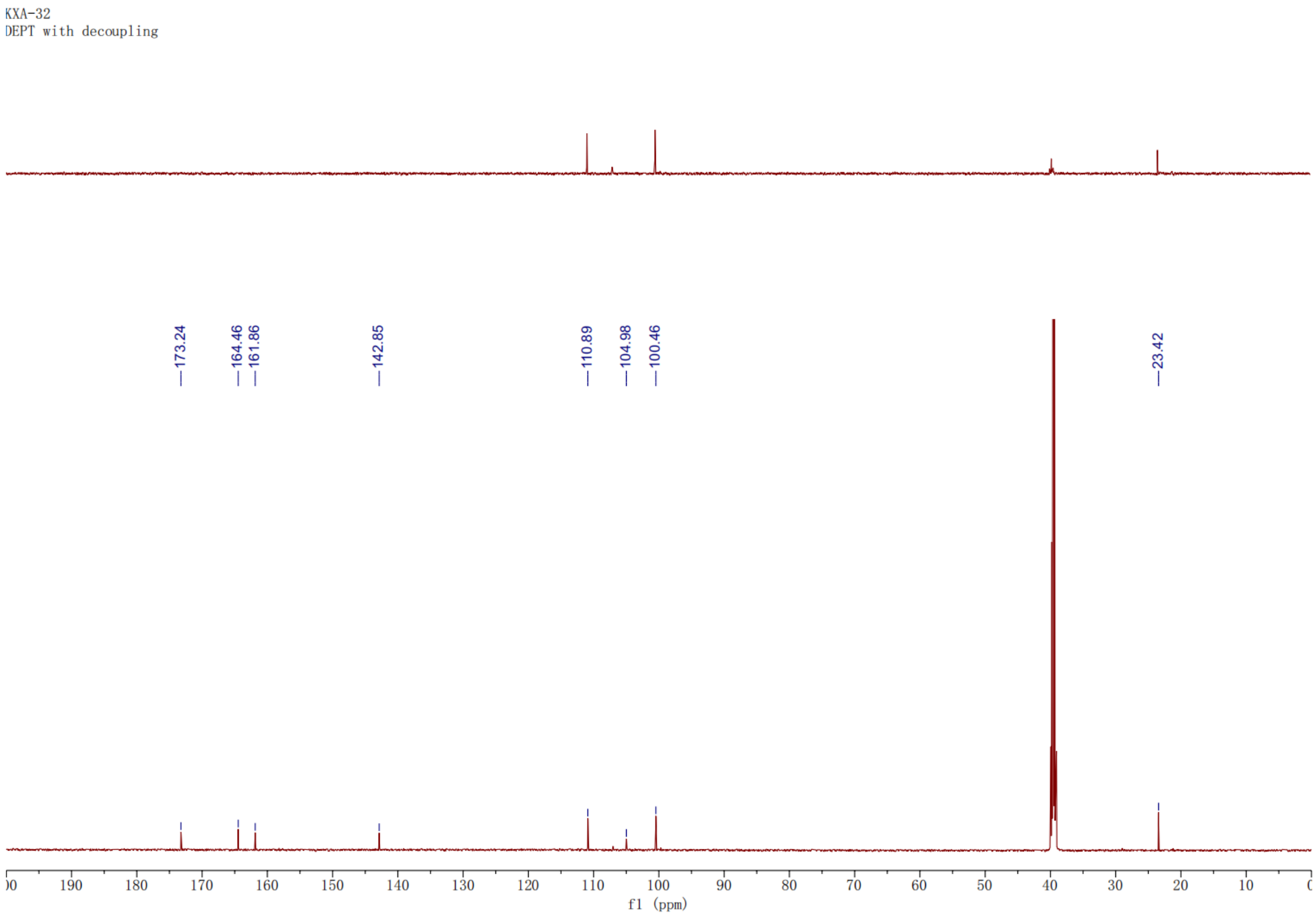

Figure S85. ^{13}C NMR (151 MHz) spectrum of compound **27** in DMSO-d_6.

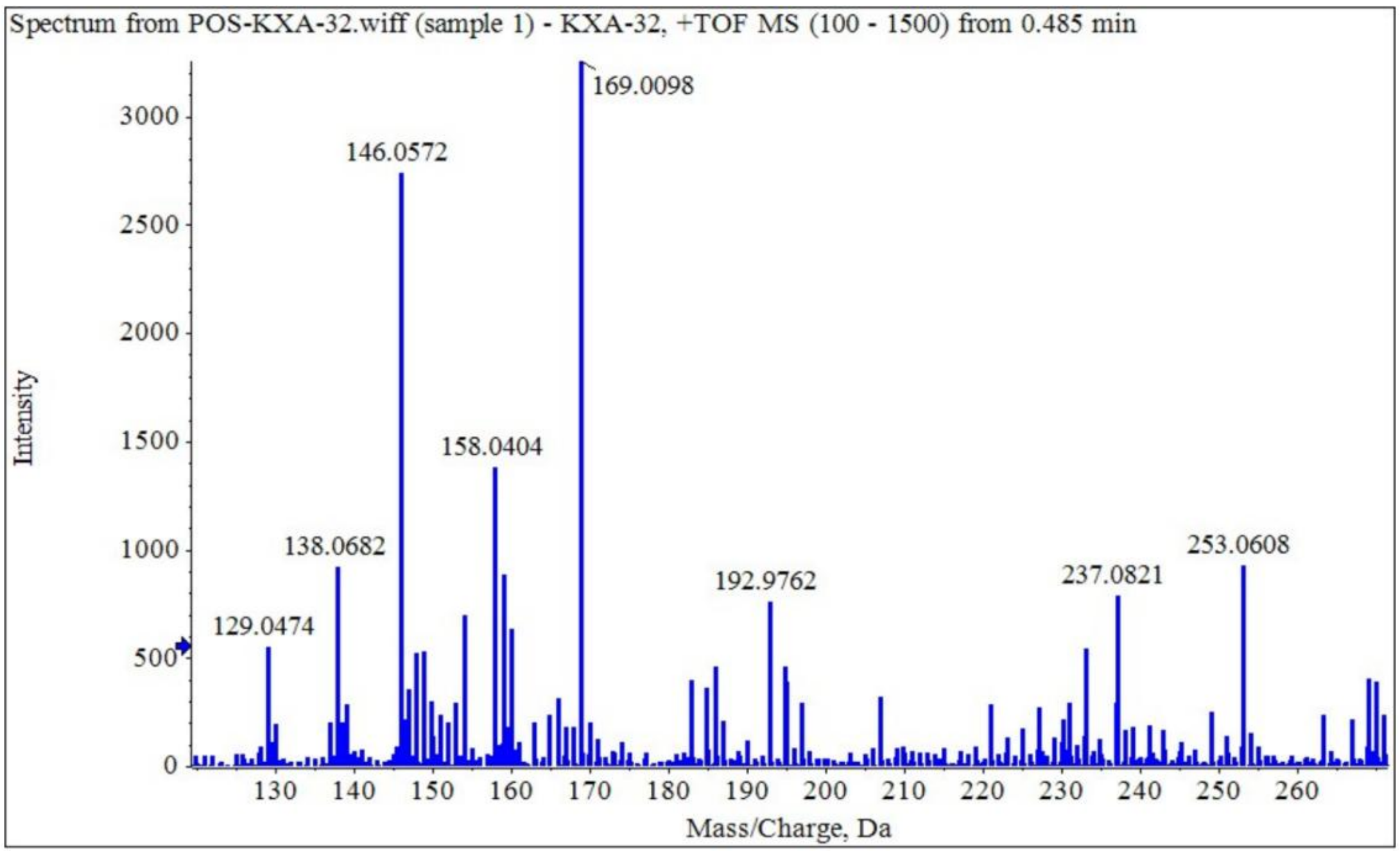

Figure S86. The positive HR-ESI-MS spectrum of compound **27**.

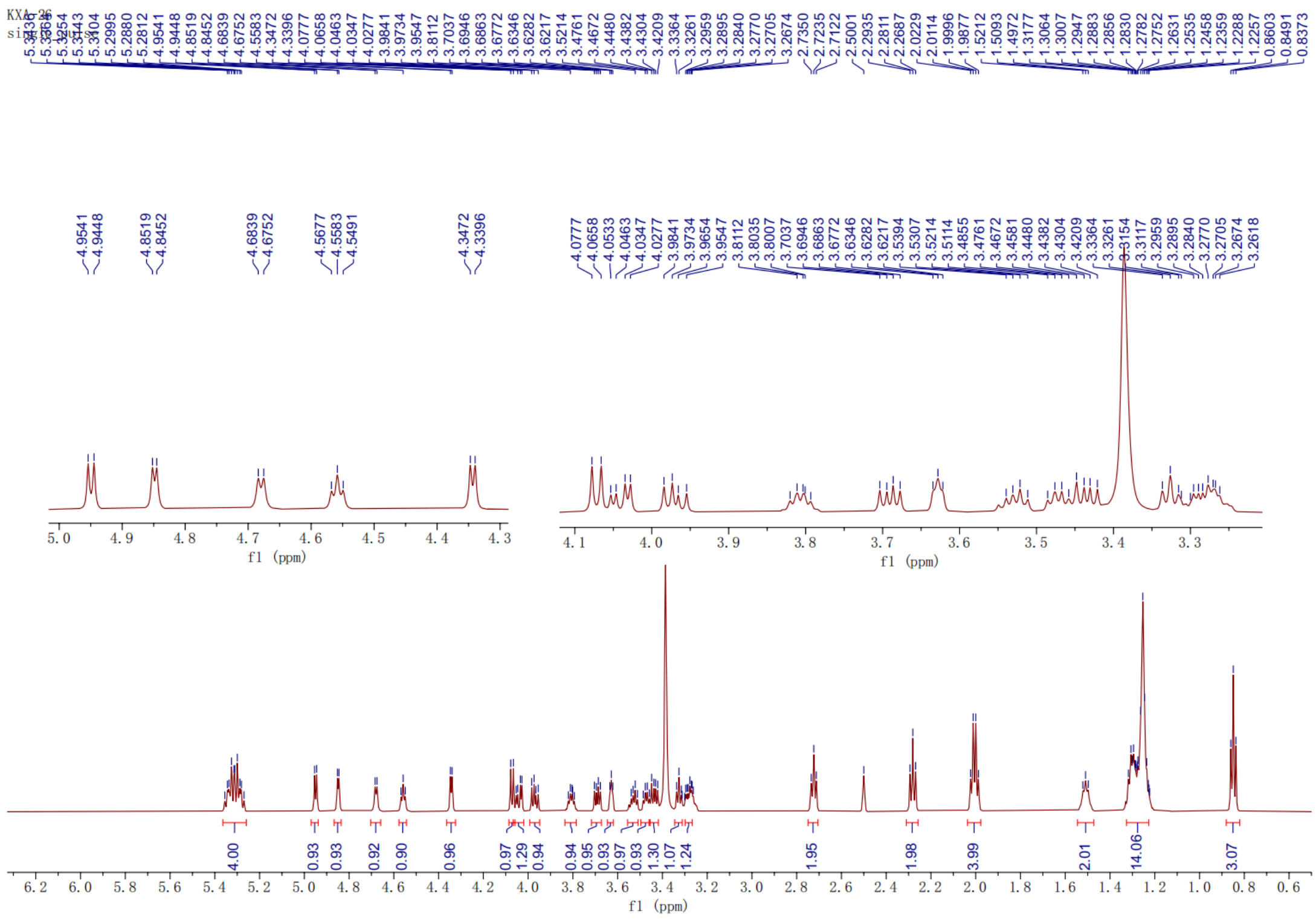

Figure S87. ^{1}H NMR (600 MHz) spectrum of compound **28** in DMSO-d_6.

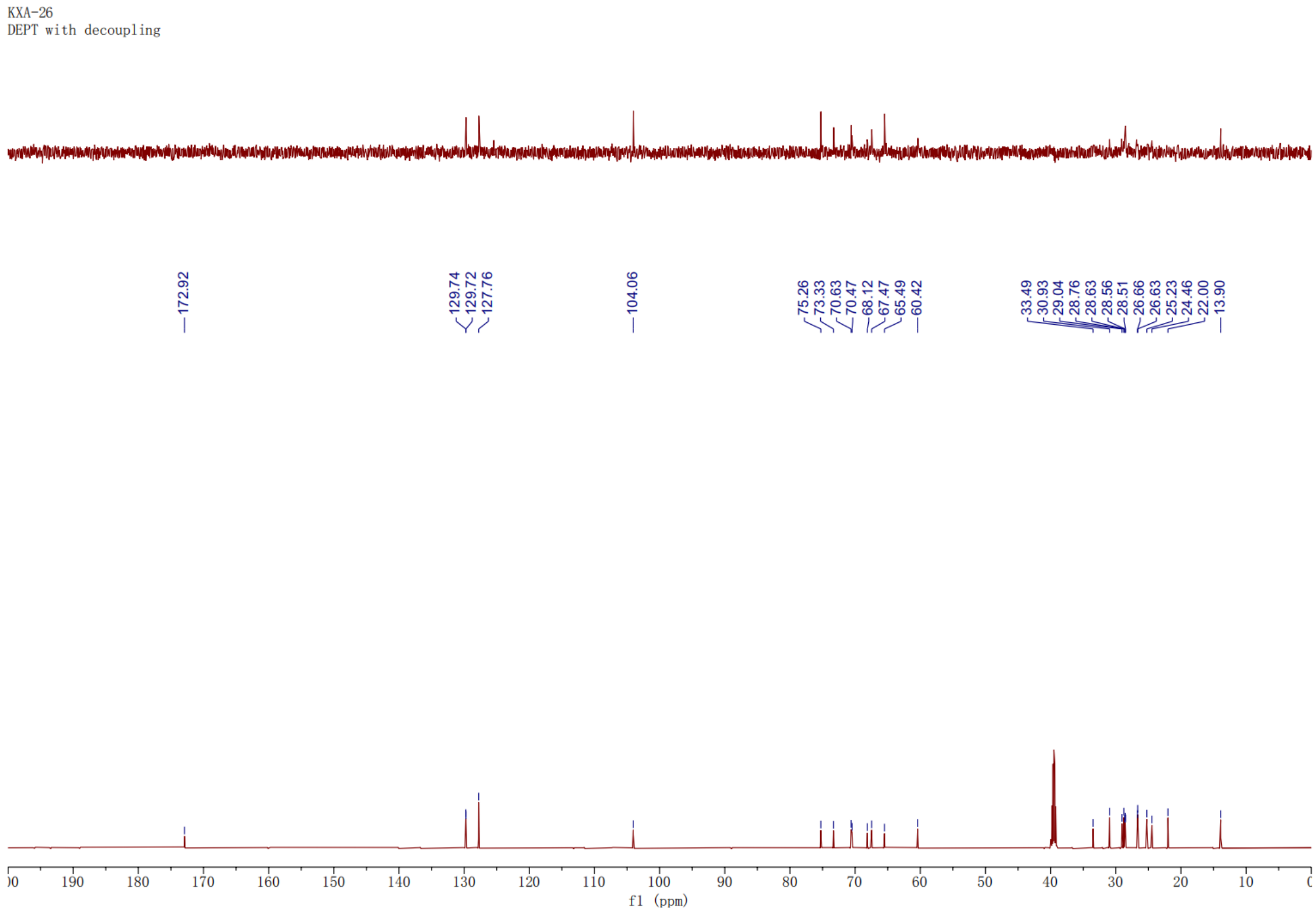

Figure S88. ^{13}C NMR (151 MHz) spectrum of compound **28** in DMSO-d_6.

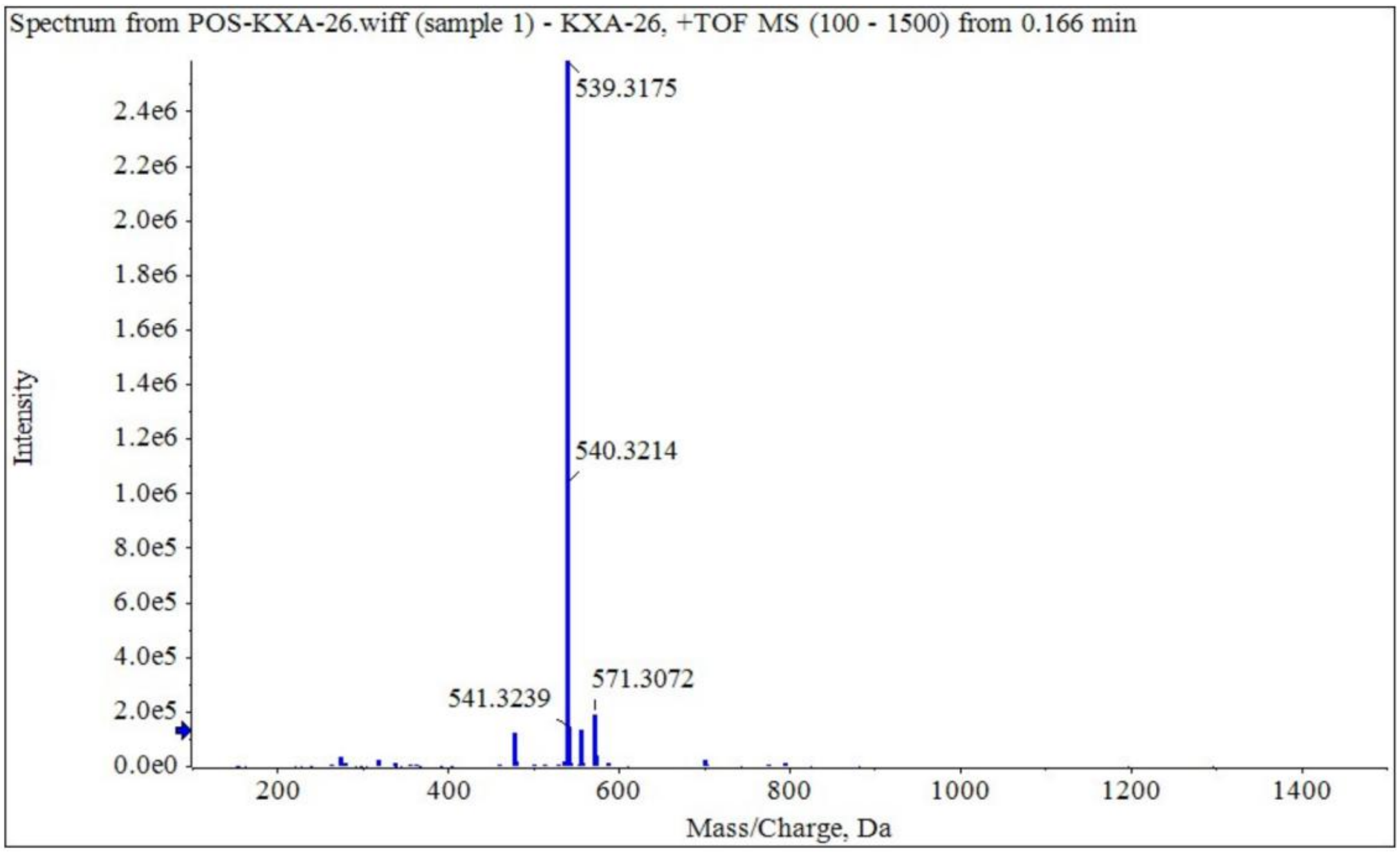

Figure S89. The positive HR-ESI-MS spectrum of compound **28**.

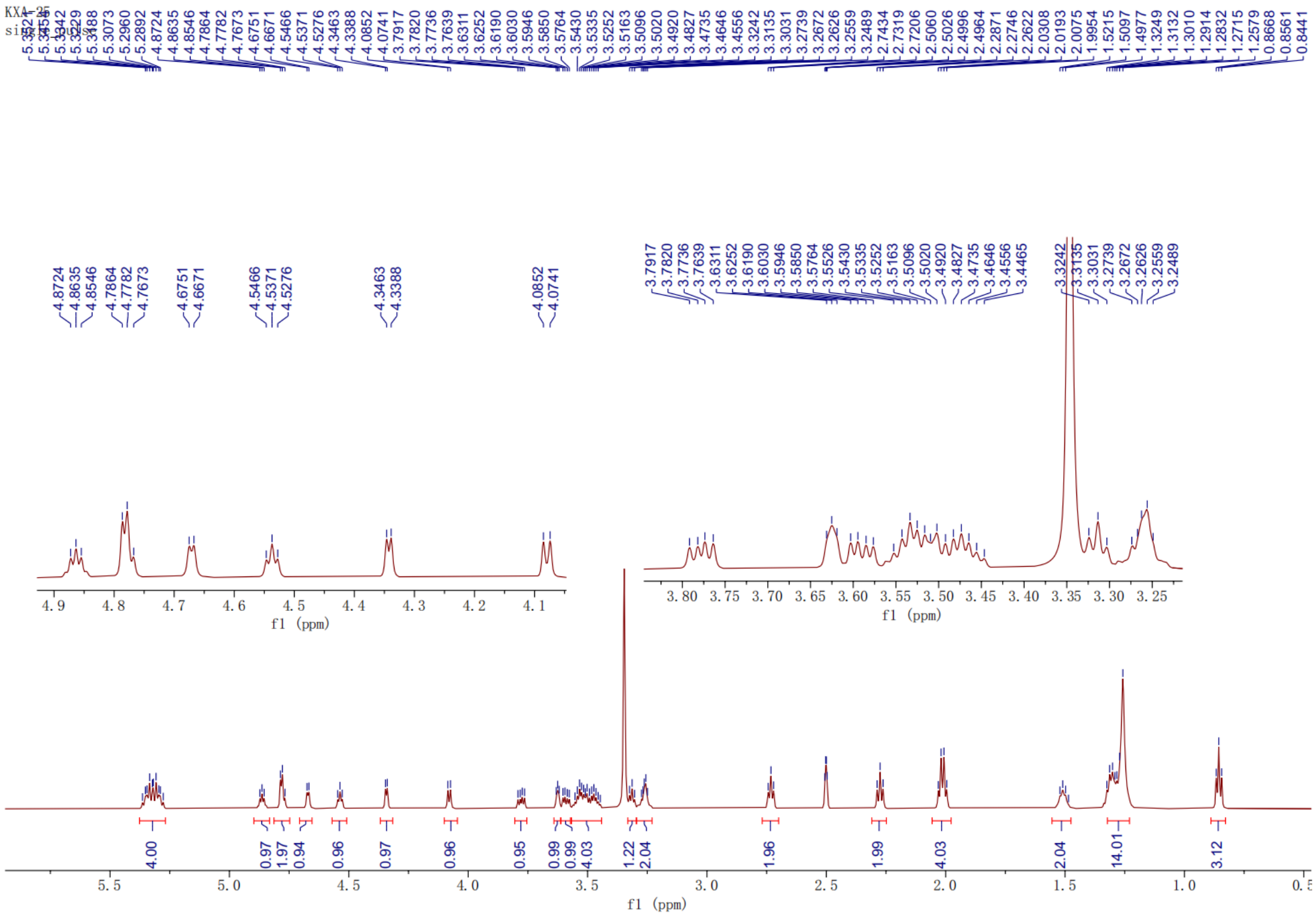

Figure S90. ^{1}H NMR (600 MHz) spectrum of compound **29** in DMSO-d_6.

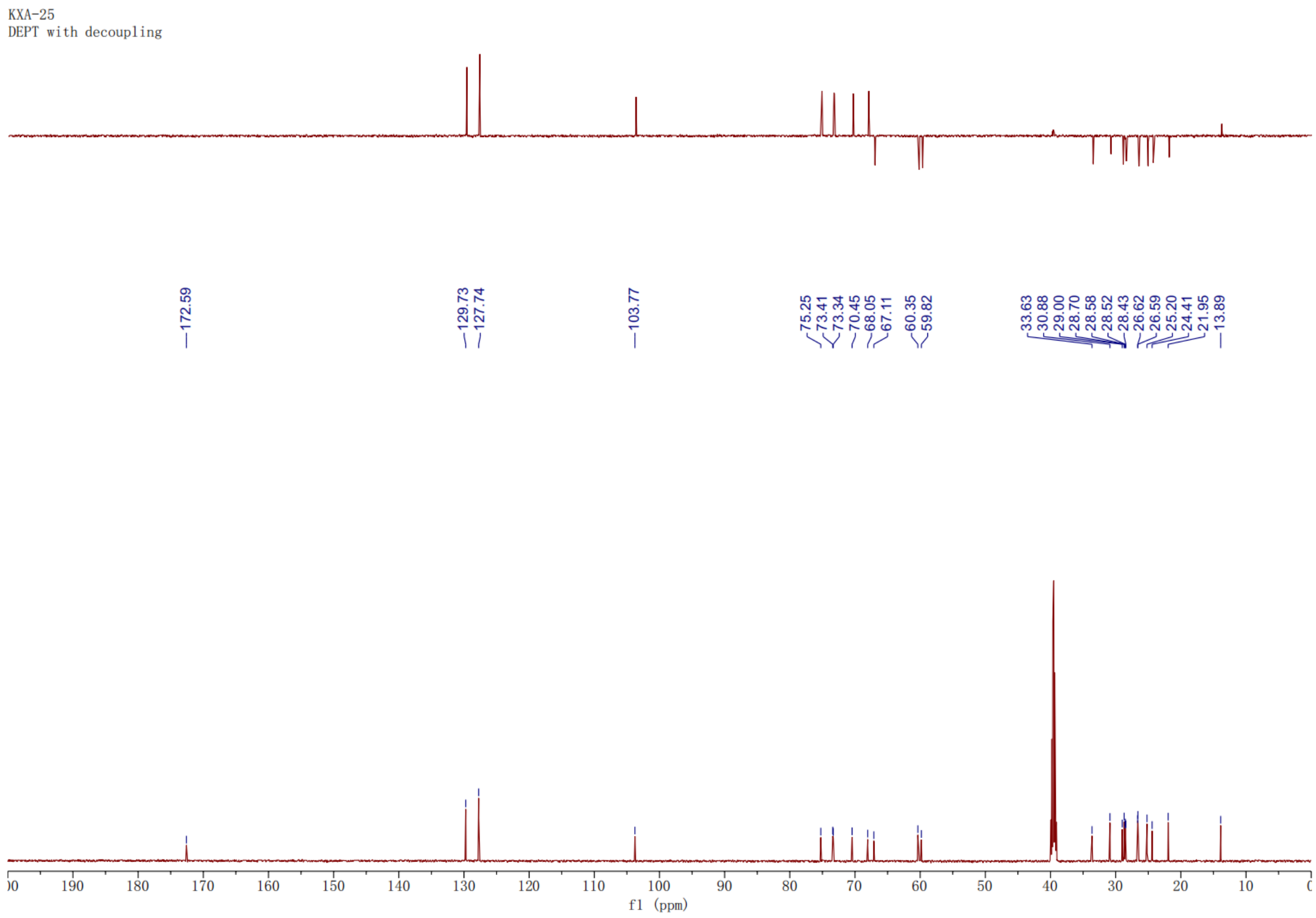

Figure S91. ^{13}C NMR (151 MHz) spectrum of compound **29** in DMSO-d_6.

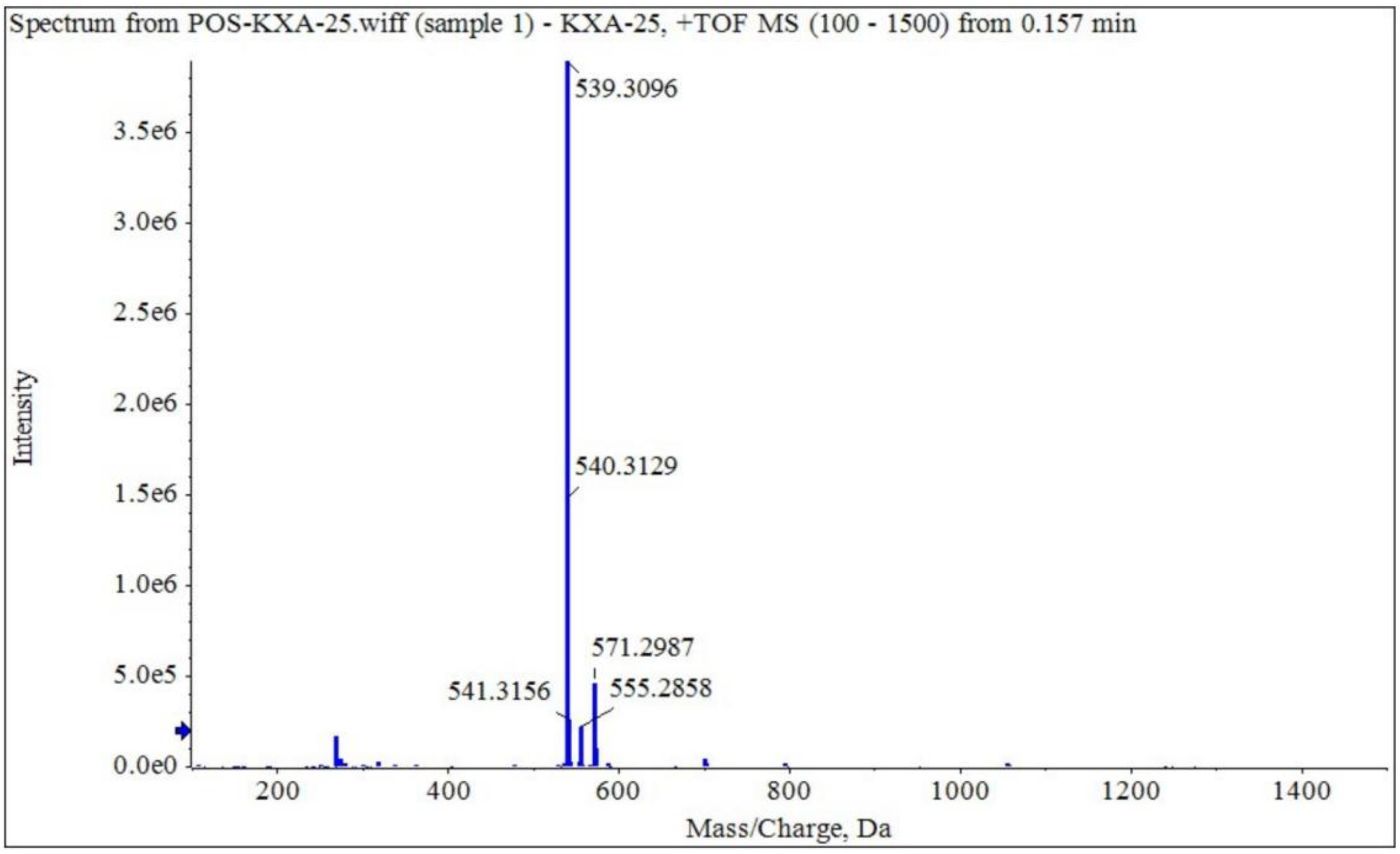

Figure S92. The positive HR-ESI-MS spectrum of compound **29**.

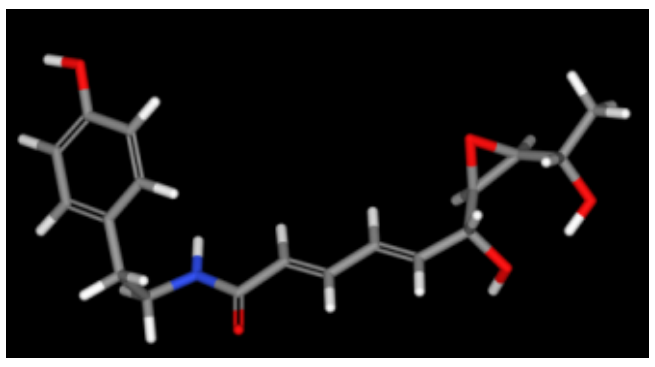

(6*R*,7*R*,8*S*,9*S*)-**1A**

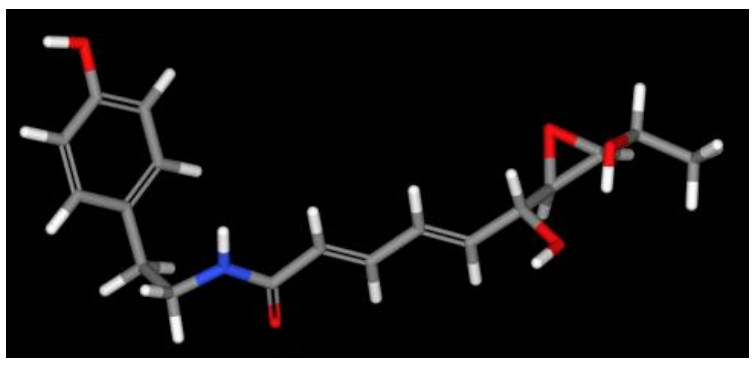

(6*R*,7*R*,8*S*,9*S*)-**1B**

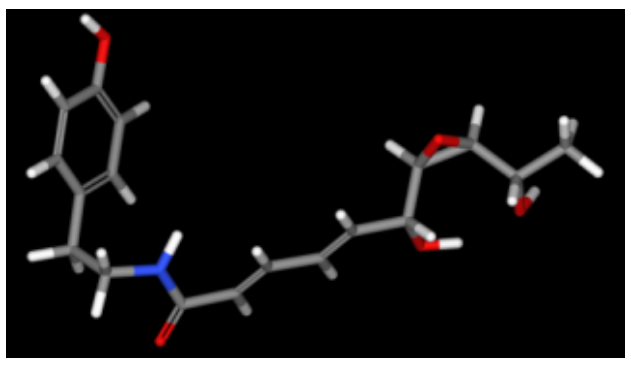

(6*R*,7*R*,8*S*,9*S*)-**1C**

Conformers	Energy (Hartree)	Energy (kcal/mol)	Population (%)	Theory level
(6*R*,7*R*,8*S*,9*S*)-**1A**	-1130.90218100	-709651.83	62.1 %	B3LYP/6-311G(d,p)
(6*R*,7*R*,8*S*,9*S*)-**1B**	-1130.90141340	-709651.35	27.6 %	B3LYP/6-311G(d,p)
(6*R*,7*R*,8*S*,9*S*)-**1C**	-1130.90041390	-709650.72	9.5%	B3LYP/6-311G(d,p)

Figure S93. Gibbs free energy and equilibrium populations of low-energy conformers of **1** in ECD calculations.
